# Discovery of Novel Polycyclic Polyprenylated Acylphloroglucinols from the Fruits of *Garcinia xanthochymus* as Antitumor Agents by Suppressing the STAT3 Signaling

**DOI:** 10.3390/ijms221910365

**Published:** 2021-09-26

**Authors:** Shan Jin, Wen Wang, Fei Gan, Wenli Xie, Jing Xu, Yu Chen, Zhinan Mei, Guangzhong Yang

**Affiliations:** 1 School of Pharmaceutical Sciences, South-Central University for Nationalities, Wuhan 430074, China; setking@mail.scuec.edu.cn (S.J.); y201804@mail.scuec.edu.cn (W.W.); 2020110478@mail.scuec.edu.cn (W.X.); xuj@mail.scuec.edu.cn (J.X.); 2 College of Chemistry and Material Sciences, South-Central University for Nationalities, Wuhan 430074, China; 2019110439@mail.scuec.edu.cn (F.G.); chenyu@mail.scuec.edu.cn (Y.C.)

**Keywords:** polycyclic polyprenylated acylphloroglucinols, *Garcinia xanthochymus*, anti-proliferative, STAT3, apoptosis

## Abstract

Pharmacologic studies have revealed that polycyclic polyprenylated acylphloroglucinols (PPAPs) collectively exhibit a broad range of biological activities, including antineoplastic potential. Here, six new PPAPs, named garcixanthochymones F–K (**3**, **5**, **7**, **8**, **11,** and **15**), together with nine known analogues were isolated from the fruits of *Garcinia xanthochymus*. Their structures were elucidated based on the spectroscopic data, including UV, HRESIMS, and NMR, and quantum chemical calculations. All the isolated PPAPs were tested for anti-proliferative activity against four human tumor cell lines, including SGC7901, A549, HepG2, and MCF-7. Most of the PPAPs possessed high anti-proliferative activity with IC_50_ values in the range of 0.89 to 36.98 μM, and significant apoptosis was observed in MCF-7 cells exposed to compounds **2** and **5**. Besides, docking results showed that compounds **2** and **5** could strongly combine with the Src homology 2 (SH2) domain of STAT3 via hydrogen bond and hydrophobic interaction, which is one of the key oncogenes and crucial therapeutic targets. Furthermore, compounds **2** and **5** efficiently downregulated the expression of p-STAT3^Tyr705^ and pivotal effector proteins involved in oncogenic signaling pathways of MCF-7 cells.

## 1. Introduction

Cancer is set to become a major cause of morbidity and mortality in the coming decades in every region of the world. By 2030, the number of cancer cases is projected to increase to 24.6 million, and the number of cancer deaths to 13 million [1]. Due to the inevitable defects of surgery, radiotherapy, and chemotherapy for cancer treatment, insights into the role of molecular targeted therapy have made natural products an attractive tool for cancer therapies which interfere with specific molecules [2].

The signal transducer and activator of transcription (STAT) family of proteins are cytoplasmic proteins with Src homology 2 (SH2) domains that function as transcription factors, responding to cytokines and growth factors [3]. Among the members of the STAT family, STAT3 has received the greatest attention since it is involved in various oncogenic signaling pathways. Once the SH2 domain is phosphorylated upon Tyr705 by Janus-like kinase (JAKs) and c-Src kinase, STAT3 forms homodimers that translocate to the nucleus and trigger the transcription of target genes involved in anti-apoptosis, angiogenesis, and invasion/migration [4,5].

Emerging evidence has found that excessive activation of STAT3 is a hallmark of many malignancies [6]. Many tumors such as breast carcinoma, lung cancer, esophagus cancer, and leukemia harbor aberrant STAT3 activity, which drives multiple pro-oncogenic functions [7,8,9].Tissue microarray showed that the expression of p-STAT3 in breast carcinoma is far above that of normal tissue and contributes to the high levels of downstream effector molecules, including apoptosis inhibitors (Survivin, Mcl-1, Bcl-XL, HSP27), cell-cycle regulators (c-Fos, MEK5, c-Myc), and inducers of tumor angiogenesis (VEGF, COX-2, MMP-2, MMP-10, MMP-1) [10]. Therefore, intensive efforts have been devoted to developing potent STAT3 inhibitors, and several small-molecule STAT3 inhibitors are currently undergoing clinical trials but have yet to be FDA approved as therapeutics. Though they have attracted less attention compared to their small-molecule counterparts, the natural product STAT3 inhibitors could provide a solid ground for new inhibitors’ design according to their biological profile and structural features [11].

*Garcinia xanthochymus* Hook. f. is an 8–20 m macrophanerophyte mainly distributed in the moist forests of valleys and hills in South-East Asia [12,13]. As a traditional Dai medicine in China, *G. xanthochymus* has been widely used for treating ardent fever, food poisoning, and diarrhea, and even used as a unique method for driving leeches out of the nasal cavity [13]. Fruits of *G. xanthochymus* have also been consumed by the indigenous people as a kind of nutritional food [12,13,14]. Pharmacological research on the extracts of *G. xanthochymus* has provided substantial evidence to support its diverse health care properties, such as antioxidant, anticancer, and anti-bacteria activity, which are closely linked to xanthones, bioflavonoids, and characteristic polycyclic polyprenylated acylphloroglucinols (PPAPs) [15,16,17]. In particular, due to their highly oxygenated and densely substituted structure and significant biological activity, PPAPs have aroused intense interest from researchers [18,19].

In our previous studies, series of PPAPs and xanthones isolated from the barks and fruits of *G. xanthochymus* exhibited good antitumor activities [20,21,22]. To explore more functional chemical constituents as novel tools for cancer therapy, we isolated and identified six new PPAPs together with nine known compounds from the edible fruits of *G. xanthochymus*. As reported previously, two pairs of the regioisomeric mixtures of PPAPs had prominent anti-tumorigenic effect on human hepatocellular carcinoma through the inactivation of STAT3 [20]; we tested the anti-proliferative activity of these PPAPs in vitro using four human tumor cell lines (SGC7901, A549, HepG2, and MCF-7), as well as the antitumor mechanism of compounds **2** and **5** in MCF-7 cells.

## 2. Results

### 2.1. Structural Elucidation of Isolated Compounds

**Figure 1:**
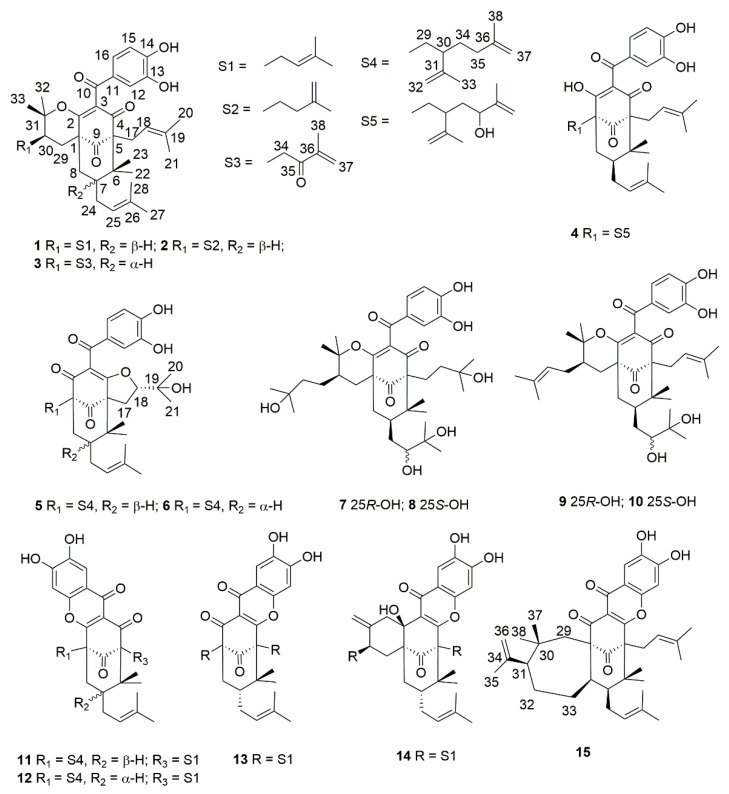
Chemical structures of PPAPs isolated from the fruits of *G. xanthochymus*.

To explore novel natural products harboring antitumor activity, we isolated fifteen compounds from the fruits of *Garcinia xanthochymus*, including six new PPAPs, named garcixanthochymones F–K (**3**, **5**, **7**, **8**, **11** and **15**), as well as nine known analogues. The molecular structure was elucidated via spectroscopic methods and quantum chemical calculations (Figure 1).

**Figure 2:**
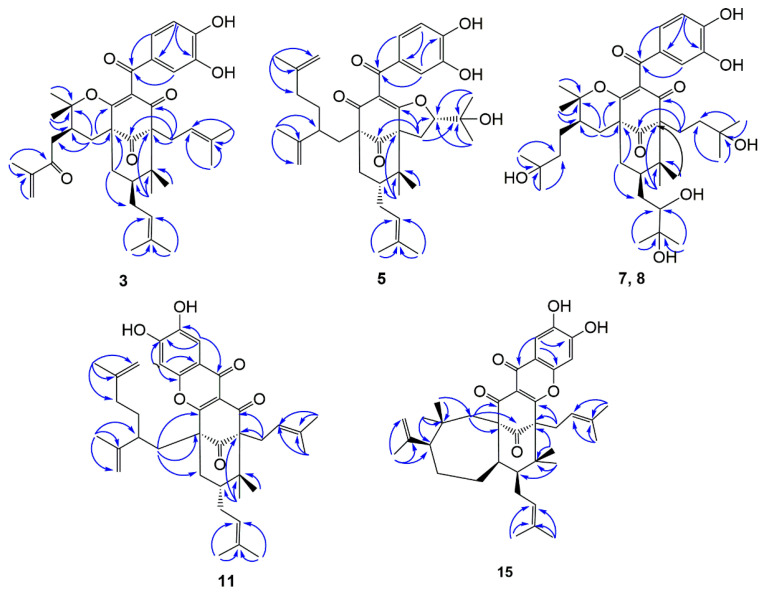
Key HMBC correlations of compounds **3**, **5**, **7**–**8**, **11**, and **15**.

**Figure 3:**
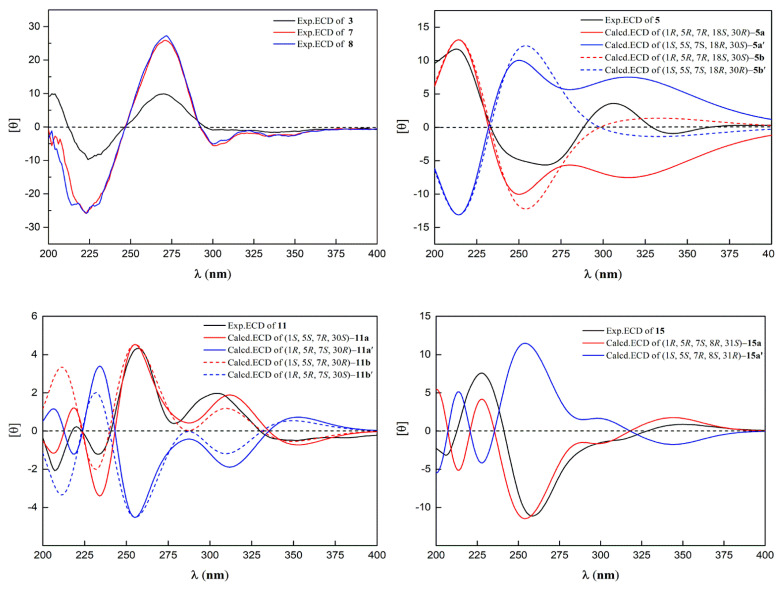
Calculated and experimental ECD spectra of compounds **3**, **5**, **7**–**8**, **11**, and **15**.

Compound **3** was isolated as a yellow powder with the molecular formula determined to be C_38_H_48_O_7_ from the molecular ion peak [M]^+^ at *m*/*z* 616.3386 (calculated for C_38_H_48_O_7_^+^, 616.3400, Figure S7). Described analysis of ^13^C-NMR, DEPT, and HSQC spectrum (Figures S1–S6) pointed to the presence of the typical and expected peaks of bicyclo[3.3.1]nonane skeleton, including a methylene at *δ*_C_ 39.9 (C-8); a methine at *δ*_C_ 47.0 (C-7); three *sp*^3^ quaternary carbons at *δ_C_* 51.4 (C-1), 69.0 (C-5), and 46.7 (C-6); a nonconjugated carbonyl carbon at *δ*_C_ 206.9 (C-9); and an enolized 1,3-diketo group at *δ*_C_ 170.9 (C-2), 126.3 (C-3), and 194.3 (C-4). The chemical shift of C-5 at *δ*_C_ 69.0 and HMBC correlations (Figure 2) from Me-22 and Me-23 to C-6 and C-5 indicated that **3** might belong to Type B bicyclic polyprenylated acylphloroglucinols (BPAPs). Comparison of the NMR data of **3** with those of the known compound cycloxanthochymol indicated that the two compounds were closely related, except for the presence of one more conjugated carbonyl carbon at *δ*_C_ 199.8 (C-35) in **3**, instead of a methylene at *δ*_C_ 35.2 (C-35) in the latter [23]. These findings suggested **3** was 35-oxo-derivative of cycloxanthochymol, which was further confirmed by the HMBC correlations from CH_3_-38 to C-35 (*δ*_C_ 199.8) and C-36 (*δ*_C_ 145.5). The relative configuration of **3** was determined by the analysis of ^13^C-NMR data. The chemical shift of C-7 and C-22 at *δ*_C_ 46.7 and 27.0 ppm, respectively, showed that the prenyl group at C-7 was in the axial position and **3** belonged to *endo*-BPAPs [24]. To further confirm the relative configuration of C-30, NMR calculations with DP4+ analysis (Tables S3 and S6) for two possible isomers (1R*, 5S*, 7S*, 30S*)-**3a** and (1R*, 5S*, 7S*, 30R*)-**3b** (Figure S55) were carried out. As a result, (1R*, 5S*, 7S*, 30S*)-**3a** showed DP4+ probability of 95.25% (Figure S60), suggesting that the relative configuration of **3** was the same as that of cycloxanthochymol. The value of [α]_D_ of **3** was positive, and the CD spectrum of **3** showed diagnostic negative and positive Cotton effects (CEs) around 220 and 270 nm (Figure 3, Figures S8 and S9), suggesting the absolute configurations of **3** were the same as those of isoxanthochymol [25]. Taken together, compound **3** was established as depicted in Figure 1, and named garcixanthochymone F.

Compound **5** was isolated as a yellow powder with the molecular formula determined to be C_38_H_50_O_7_ from the *pseudo*-molecular ion peak [M + Na]^+^ at *m*/*z* 641.3452 (calculated for C_38_H_50_O_7_Na^+^, 641.3454, Figure S16). The ^1^H and ^13^C NMR data (Table 1 and Table 2, Figures S10–S14) of **5** were close to those of nujiangefolin C [26], except for obvious differences in the chemical shifts at C-7 and C-22. The upfield shifts of C-7 and C-22 (−5.0 ppm and −11.1 ppm) in **5** indicated that the prenyl group at C-7 was in an equatorial position and **5** belonged to *exo*-BPAPs [24], suggesting **5** was the 7-epimer of nujiangefolin C. ROESY correlation (Figure S15) between CH_3_-23 and H-18 confirmed this deduction. To further determine the absolute configuration of **5**, the ECD calculations for (1*R*, 5*R*, 7*R*, 18*S*, 30*R*)-**5a**, (1*R*, 5*R*, 7*R*, 18*S*, 30*S*)-**5b** (Figure S56), and their enantiomers were performed using the TDDFT/ECD method at the B3LYP/6-31+G(d) level (Tables S1 and S2). The calculation results showed that the calculated ECD curves of **5a** and **5b** were consistent with the experimental ECD spectrum (Figure 3, Figures S17, S18 and S63), so it was impossible to distinguish 30*R* and 30*S* isomers by this method. Therefore, the absolute configuration of **5,** except C-30, was established as depicted in Figure 1, and named garcixanthochymone G.

Compounds **7** and **8** were isolated as yellow powders with the same molecular formula of C_38_H_56_O_10_ as revealed by the *pseudo*-molecular ion peak [M + Na]^+^ at *m*/*z* 695.3766 in **7** and 695.3773 in **8** (calculated for C_38_H_56_O_10_Na^+^, 695.3711, Figures S25 and S34). Analyses of their NMR data (Figures S19–S24 and S28–S33) suggested that they also possessed identical planar structures and were structurally related to coccinones D and E [27]. Comparing the NMR data of **7** and **8** with the NMR data of coccinones D and E, it was found that they are different from coccinones D and E in C-1 and C-5 substituents. Two prenyl groups at C-1 and C-5 in coccinones D and E were replaced by two 3-hydroxy-3-methylbutyl groups in **7** and **8**. HMBC correlations from CH_3_-20, 21 to C-18 and C-19, CH_3_-37, 38 to C-35 and C-36, and H_2_-17 to C-4 and C-9 confirmed these findings. Detailed analyses of the NMR data of **7** and **8** indicated that the difference between **7** and **8** was the absolute configuration of C-25, which was the same as those of coccinones D and E. Unfortunately, the absolute configurations of C-25 of occinones D and E were not determined by Mosher method, owing to an insufficient amount of both compounds. It was found that hypersampsones V and W contained 2, 3-dihydroxy-3-methylbutyl group by consulting the literature [28]. The absolute configurations of the 1, 2-diol moiety in hypersampsones V and W were determined by in situ dimolybdenum CD method. In the case of 2*R*, 3-dihydroxy-3-methylbutyl group, the chemical shifts of C-25 were located at about *δ*_C_ 77 ppm. On the contrary, with 1, 2-diol moiety adopted as S configuration, the chemical shift of C-25 was located at about *δ*_C_ 80 ppm. Based on these rules, the absolute configurations of C-25 of **7** and **8** were determined as *R* and *S,* respectively. To further confirm the relative configuration of C-30, DP4+ analysis (Tables S4 and S6) for two possible isomers, (1R*, 5S*, 7R*, 25R*, 30R*)-**7a** and (1R*, 5S*, 7R*, 25R*, 30S*)-**7b** (Figures S57 and S61), suggested that the relative configuration of C-30 was the same as that of isoxanthochymol. The absolute configurations of **7** and **8** were established by the comparison of their experimental ECD curves with that of isoxanthochymol (Figure 3, Figures S26, S27, S35 and S36). Therefore, compounds **7** and **8** were established as depicted in Figure 1, and named garcixanthochymones H and I, respectively.

Compound **11** was isolated as a yellow powder with the molecular formula determined to be C_38_H_48_O_6_ from the *pseudo*-molecular ion peak [M + H]^+^ at *m*/*z* 601.3528 (calculated for C_38_H_49_O_6_^+^, 601.3529, Figure S43). Compared to the NMR data (Figures S37–S42) of the above four compounds, it was found that there was no signal of 3,4-dihydroxybenzoyl in the ^1^H-NMR of **11**, but there were two isolated aromatic proton signals at *δ*_H_ 8.11 (1H, s) and 7.26 (1H, s). Moreover, the carbonyl signal of 3,4-dihydroxybenzoyl was shifted upfield from about *δ*_C_ 195 to *δ*_C_ 172.4 in **11**. Therefore, compound **11** belonged to tetracyclic xanthones derived from the oxidation product of the corresponding BPAPs [29]. Type B BPAPs that possess a catechol moiety are prone to be oxidized at C-16 and further cyclized with 2-hydroxy or 4-hydroxy of the enolized diketone to form a fused tetracyclic xanthone [30]. In the case of the ether ring closure between C-16 and C-2, Δ*δ*_C_ between C-5 and C-1 was more than 10 ppm. On the contrary, the ether linkage formed between C-16 and C-4 and the chemical shifts of C-1 and C-5 were almost the same [29]. Compound **11** was established as an ether linkage formed between C-16 and C-2 based on the value of 16.5 ppm of Δ*δ*_C5-C1_. Comparing the NMR data of **11** with those NMR data of nujiangefolin B indicated that they distinguished from nujiangefolin B at the relative configuration of C-7 [26]. The chemical shifts of C-7 and C-22 in **11** were located at *δ*_C_ 42.9 and 16.4, suggesting that the prenyl group at C-7 was in an equatorial position. Like compound **5**, the absolute configuration of compound **11,** except C-30, might be determined as (1*S*, 5*S*, 7*R*) by ECD calculation method (Figure 3, Figures S44, S45, S58 and S64). Therefore, compound **11** was established as 7-epi-nujiangefolin B, and named garcixanthochymone J.

Compound **15** was isolated as a yellow powder with the molecular formula determined to be C_38_H_48_O_6_ from the *pseudo*-molecular ion peak [M + Na]^+^ at *m*/*z* 623.3345 (calculated for C_38_H_48_O_6_Na^+^, 623.3349, Figure S52). The NMR data of **15** (Figures S46–S51) was similar to that of guttiferone H [31], expect for the presence of a 1,2,4,5-tetrasubstituted aromatic ring at *δ*_H_ 8.10 (1H, s) and 7.45 (1H, s), instead of the presence of a 3,4-dihydroxybenzoyl in guttiferone H. These findings implied that **15** was the oxidation product of guttiferone H. The chemical shifts of C-1 and C-5 were located at *δ*_C_ 66.7 and 64.6, suggesting that the ether linkage was formed between C-16 and C-4. This deduction was further confirmed by HMBC correlations. In the same way, compound **15** was determined as *endo*-BPAPs by ^13^C-NMR analysis. Unfortunately, the stereochemistry of guttiferone H has not been determined in the literature. At present, except for the determination of the relative configurations of C-1, C-5, and C-7 of **15**, the relative configurations of C-8 and C-31 could not be determined by ROESY spectrum due to the lack of useful ROESY correlations. Therefore, there might be four possible isomers for **15**: (1R*, 5R*, 7S*, 8R*, 31S*)-**15a**; (1R*, 5R*, 7S*, 8R*, 31R*)-**15b**; (1R*, 5R*, 7S*, 8S*, 31S*)-**15c;** and (1R*, 5R*, 7S*, 8S*, 31R*)-**15d** (Figure S59). NMR calculations with DP4+ analysis (Figure S62, Tables S5 and S6) for four possible isomers were performed. As a result, (1R*, 5R*, 7S*, 8R*, 31S*)-**15a** was the most likely structure based on DP4+ probability with 100%. By comparing the experimental ECD spectrum with the calculated ECD spectrum (Figure 3, Figures S53, S54 and S65), the absolute configuration of **15** could be defined as (1*R*, 5*R*, 7*S*, 8*R*, 31*S*). Therefore, compound **15** was established as depicted in Figure 1, and named garcixanthochymone K.

These nine known compounds were identified as 7-epi-isoxanthochymol (**1**) [32], 7-epi-cycloxanthochymol (**2**) [32], garcimultiforone E (**4**) [33], nujianggefolin C (**6**) [26], coccinone D (**9**) [27], coccinone E (**10**) [27], nujiangefolin B (**12**) [26], garcimultiforone I (**13**) [34], and symphonone I (**14**) [35] by comparison of the spectroscopic data (Tables S7–S13) with those reported in the literature.

### 2.2. Anti-Proliferative Activity of the Isolated PPAPs on Human Tumor Cell Lines

All isolated PPAPs may be categorized into two types: BPAPs (**1**–**10**) and the oxidation product of the corresponding BPAPs, named as oxy-BPAPs (**11**–**15**). The anti-proliferative activity of the fifteen BPAPs was evaluated by MTT assay, as shown in Table 3. Except for compounds **7** and **8**, all BPAPs showed good anti-proliferative activity against the four human tumor cell lines (SGC7901, A549, HepG2, and MCF-7). These findings implied that the 3,4-dihydroxybenzoyl group, enolic β-diketone system, and double bonds of the prenyl groups in BPAPs were crucial for their anti-proliferative activities [24,30]. Notably, it is intriguing to find that the anti-proliferative activity is related to the fine changes in the structure of oxy-BPAPs. For example, although compounds **11** and **12** are a pair of epimers, the anti-proliferative activity of compound **12** is stronger than that of compound **11**. Except for compound **12**, the anti-proliferative activity of BPAPs is stronger than that of oxy-BPAPs. All the results above suggested that most of these PPAPs from the fruit of *G. xanthochymus* could inhibit the growth of cancer cells, so PPAPs may be an effective ingredient of *G. xanthochymus* contributing to cancer treatment.

### 2.3. Compounds ***2*** and ***5*** Induced Cellular Apoptosis of MCF-7

**Figure 4:**
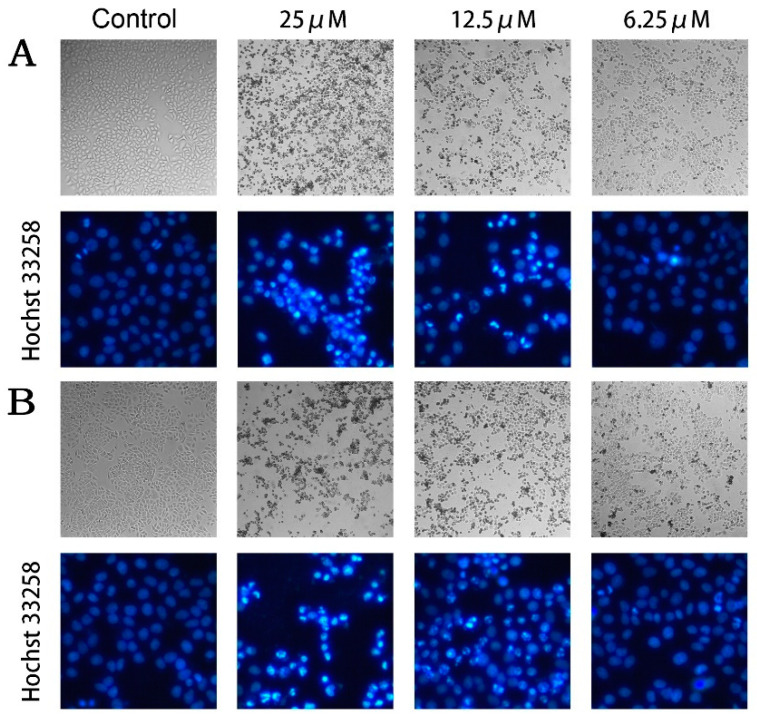
Compounds **2** and **5** induced apoptosis of MCF-7 cells. Morphological changes of cells exposed to various doses of compounds **2** (**A**) and **5** (**B**) for 24 h analyzed by microscopy observation (upper panels) in 100× and Hoechst 33258 fluorescent staining (below panels) in 400×.

Among these PPAPs harboring good anti-proliferative activity against tumor cells, compounds **2** and **5** were selected for further study of antitumor mechanism in consideration of cytotoxicity and sample volume. To explore the pro-apoptosis effect of PPAPs on tumorigenesis, we observed the cellular morphological changes through microscope, and found that extensive cell shrinkage and blebbing occurred in MCF-7 cells treated with compounds **2** and **5** for 24 h (Figure 4A,B). Besides, Hoechst 33258 staining was performed for further observation of nuclear changes in morphology. The results demonstrated that compounds **2** and **5** induced significant nuclear condensation in a dose-dependent manner (Figure 4A,B), which is believed to be a hallmark of apoptosis. These data indicated that compounds **2** and **5** may suppress the excessive proliferation of tumor cells by activating the apoptosis signaling pathway.

### 2.4. Docking Analysis of Compounds ***2*** and ***5*** with STAT3

**Figure 5:**
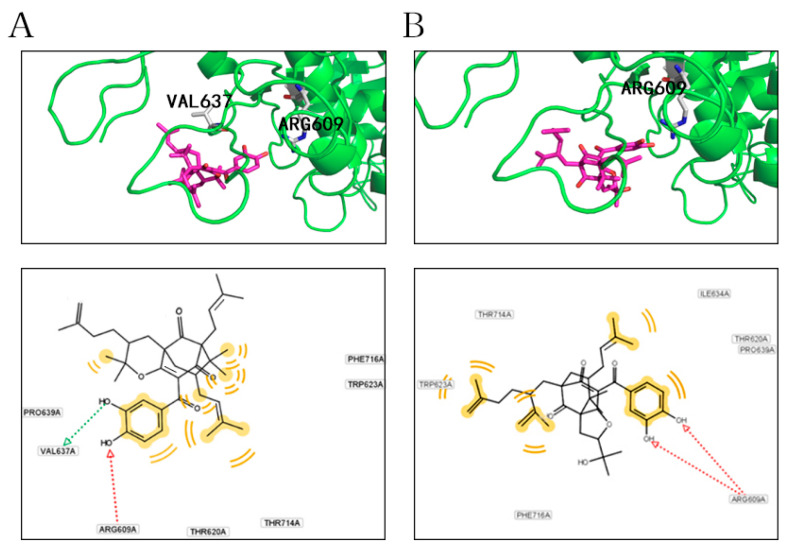
Virtual molecular docking pattern of compounds **2** (**A**) and **5** (**B**) with the SH2 domain of STAT3 crystal (1BG1). Green cartoon refers to protein structure, blue sticks refer to compound molecules, and yellow dashed lines refer to hydrogen bonds, with the number on the dashed line representing distance (Å).

The molecular modeling of compounds **2** and **5** with the SH2 domain (580–670 aa) of the STAT3 crystal (1BG1) were performed to depict the binding profile. As shown in Figure 5, compound **2** could interact with ARG609 and VAL637 through hydrogen bonds and form hydrophobic interactions with THR714, PHE716, TRP623, PRO639, and THR620, respectively (Figure 5A). Compound **5** could interact with ARG609 through hydrogen bonds and form hydrophobic interactions with ILE634, THR620, PRO639, PHE716, THR714, and TRP623, respectively (Figure 5B). Accordingly, both compounds **2** and **5** could interact with the SH2 domain of STAT3 via hydrogen bonds and hydrophobic interaction, which are the main driving force for receptor–ligand combination, suggesting that they may play a role in STAT3 signaling.

### 2.5. Compounds ***2*** and ***5*** Suppressed the Phosphorylation of STAT3 in MCF-7

**Figure 6:**
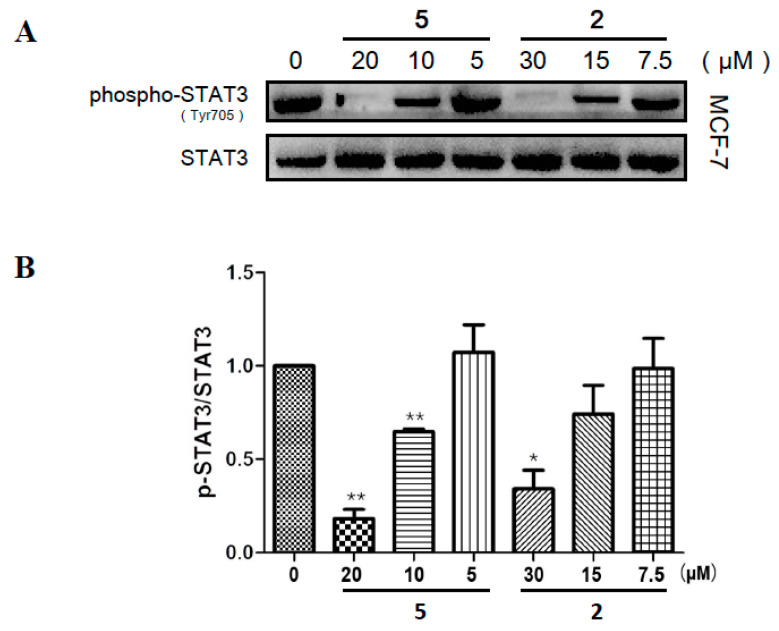
Inhibitory effects of compounds **2** and **5** on the phosphorylation of STAT3 on Tyr705 in MCF-7 cells. The cells were incubated with compound **2** or **5** for 6 h with the indicated concentrations. (**A**) The protein levels of pSTAT3^Tyr705^ and total STAT3 were detected by western blotting. (**B**) Fold changes of western blotting (*n* = 3). Data represent mean ± SD. * *p* < 0.05, ** *p* < 0.01 compared with the control group.

To elucidate whether the anti-tumor activity of compounds **2** and **5** in vitro was related to STAT3 signaling, the level of p-STAT3^Tyr705^ was measured in MCF-7 cells by Western blotting. The results in Figure 6A proved that treatment of compounds **2** and **5** decreased the phosphorylation of STAT3 on Tyr705 in a dose-dependent manner. As presented in Figure 6B, the relative ratio of p-STAT3^Tyr705^/STAT3 were efficiently suppressed separately by 20 μM (*p* < 0.01) compound **5**, 10 μM (*p* <0.01) compound **5,** and 30 μM (*p* < 0.05) compound **2**. Consistent with the results from docking analysis, compound **5** had a superior inhibitory effect on STAT3 phosphorylation than compound **2**. Thus, we preliminarily attributed the favorable antitumor effect of *G.* *xanthochymus* to the suppressive activity of PPAPs on STAT3 activation.

### 2.6. Compounds ***2*** and ***5*** Downregulated the Expression of Effector Genes Downstream of STAT3 Signaling

**Figure 7:**
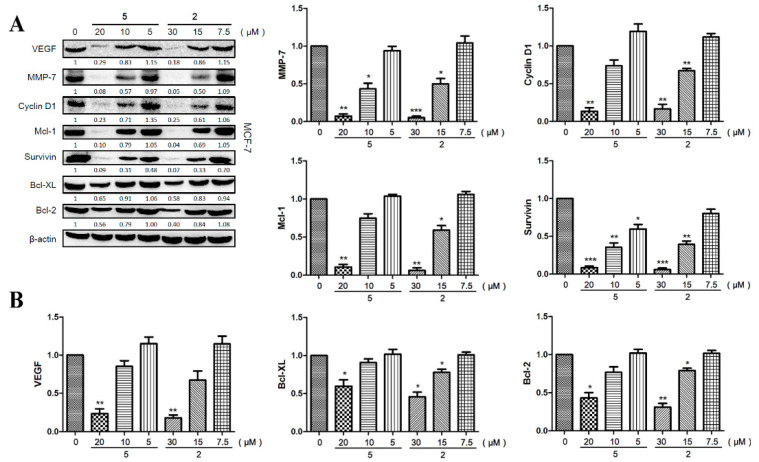
Suppressive effects of compounds **2** and **5** on the expression of downstream effector proteins of STAT3 in MCF-7cells. The cells were incubated with compound **2** or **5** for 6 h with the indicated concentrations. (**A**) The protein levels of VEGF, MMP-7, Cyclin D1, Mcl-1, Survivin, Bcl-XL, Bcl-2, and β-actin were detected by western blotting. (**B**) Fold changes of Western blotting (*n* = 3). Data represent mean ± SD. * *p* < 0.05, ** *p* < 0.01, *** *p* < 0.001 compared with the control group.

Activated STAT3 can regulate the expression of various genes involved in cancer pathogenesis, including angiogenesis, tumor migration, and cell survival [4,6]. Thus, we used MCF-7 cells to evaluate the effects of compound **2** and **5** on the expression of VEGF, MMP-7, Cyclin D1, Mcl-1, Survivin, Bcl-XL, and Bcl-2 by Western blotting. The results demonstrated that both compounds **2** and **5** could reduce the protein levels of these genes dose-dependently (Figure 7A,B). Specifically, a high dose of compound **2** (30 μM) and compound **5** (20 μM) significantly decreased the expression of VEGF (*p* < 0.01/*p* < 0.01), MMP-7 (*p* < 0.001/*p* < 0.01), Cyclin D1 (*p* < 0.01/*p* < 0.01), Mcl-1 (*p* < 0.01/*p* < 0.01), Survivin (*p* < 0.001/*p* < 0.001), Bcl-XL (*p* < 0.05/*p* < 0.05), and Bcl-2 (*p* < 0.01/*p* < 0.05). Therefore, compounds **2** and **5** played an anti-tumor role via inhibiting the expression of pro-oncogenic genes induced by activated STAT3.

## 3. Discussion

*Garciniaxanthochymus*, commonly known as an edible fruit and medicine, is native to Polynesia, South-East Asia, Africa, Australia, north Thailand, Myanmar, and Yunnan of China [13]. As a traditional ethnomedicine of Dai in China, it is widely used for treating diarrhea and food poisoning [13]. Several studies proved that *Garciniaxanthochymus* possesses cytotoxic activity against various types of cancer [12]. Here, six new natural products named garcixanthochymones F–K (**3**, **5**, **7**, **8**, **11,** and **15**) were isolated from the fruits of *Garcinia* *xanthochymus* and identified as polycyclic polyprenylated acylphloroglucinols (PPAPs) for the first time via spectroscopic methods and quantum chemical calculations.

PPAPs are a class of characteristic components in the genus *Garcinia* sharing considerable structure and bioactivity diversity [18]. All of the PPAP profiles are generated via three major biosynthetic pathways and may be divided into three groups (I−III) according to their different scaffolds [36].The bicyclic polyprenylated acylphloroglucinols (BPAPs) with major bicyclo[3.3.1]nonane-2,4,9-trione core and related *seco*-BPAPs are classified as group I and comprise approximate 60% of PPAPs [30].Most of the type B BPAPs are obtained from the genus *Garcinia*, and the majority of them share a characteristic hydroxylated benzoyl group [30]. In recent studies, emerging evidence has indicated that several BPAPs are antitumor agents [37,38,39]. For example, garcinol, obtained from *Garcinia indica* and several other *Garcinia* plants, exhibits excellent cytotoxicity and anti-tumorigenesis activity by inhibiting various key signaling pathways involved in cell survival and proliferation of cancer cells, such as NF-κB and STAT3 [40]. In this study, results of MTT assay using four human cell lines (HepG2, A549, SGC7901, and MCF-7) revealed that most of the PPAPs we isolated from *Garcinia* *xanthochymus* exhibited good anti-proliferative activity *in vitro*. Microscopic observation of cellular morphological changes and Hoechst 33258 staining furtherly confirmed that compounds **2** and **5** may inhibit tumorigenesis by activating the apoptosis signaling pathway in MCF-7 cells.

The transcription factor signal transducer and activator of transcription STAT3 is activated downstream of cytokines, growth factors, and oncogenes to mediate their functions under both physiological and pathological conditions [41]. Excessive activation of STAT3 is detected in a wide variety of tumors, and the specificity in signaling of STAT3 is mediated by the SH2 domain, which mediates its interaction with the phosphopeptide docking sites displayed by receptors and JAKs, dimerization, and subsequent DNA binding [42].Docking analysis indicated that compounds **2** and **5** could form hydrogen bonds and hydrophobic interactions with the STAT3 crystal (1BG1), leading to the phosphorylation on Y705 (YP) of SH2, which made active STAT3 dimers concentrate in the nucleus to regulate the expression of target genes. Western blotting results demonstrated that both compounds **2** and **5** dose-dependently reduced the expression level of p-STAT3^Tyr705^ in MCF-7 cells, suggesting that they suppressed STAT3 signaling by interrupting the phosphorylation of the SH2 domain on Y705.

As reported, STAT3 downstream target genes are crucial to the dysregulated biological processes promoted by aberrantly active STAT3 [41]. Several anti-apoptotic proteins, such as Survivin and members of the Bcl family (Bcl-XL, Bcl-2, and Mcl-1), which are essential for tumor cell survival, are direct target genes of STAT3 and downregulated as a consequence of STAT3 inhibition [43]. Besides, VEGF and several members of the MMP family downstream of STAT3 have been proven to contribute to tumor invasion, angiogenesis, and metastasis in various cancer cells. For example, STAT3 has been found to be involved in colorectal cancer cell growth, survival, invasion, and migration through regulation of gene expression, such as Bcl-2, p21waf1/cip1, p27kip1, E-cadherin, VEGF, and MMPs [42,43]. As described in Figure 5, compounds **2** and **5** effectively decreased the protein levels of anti-apoptotic (Bcl-XL, Bcl-2, Mcl-1, and Survivin) and proliferative (cyclin D1) genes in MCF-7 cells. For a future study, we plan to focus on the antitumor activity of compounds **2** and **5** in vivo via mouse xenograft experiments if enough of compounds **2** and **5** can be obtained. Suspended MCF-7 cells are to be subcutaneously injected into each flank of 4–6-week-old BALB/c nu/nu female mice and/or injected into the lateral tail veins of nude female mice to analyze the antiproliferative and antimetastatic effects of these two PPAPs. As there has been no STAT3-targeted drug approved for clinical application, natural products which inhibit STAT3 to achieve the cytotoxic activity in breast cancer studies could be selected as a positive control, such as JSI124 (cucurbitacin I). Besides the anti-tumor efficacy, the unwanted side effects as well as drug costs would also be taken into account for a comparative study. On the other hand, the significant downregulation of VEGF and MMP-7 suggested that compounds **2** and **5** also exhibited good effects in suppressing tumor invasion, angiogenesis, and metastasis, which need to be further verified via functional experiments in vitro and in vivo.

## 4. Materials and Methods

### 4.1. General

Optical rotations were measured in MeOH on a P-1020 polarimeter (JASCO, Tokyo, Japan). UV spectra were obtained on a 2401PC spectrophotometer (Shimadzu Co., Tokyo, Japan), while 1D and 2D NMR spectra were recorded on a Bruker AVANCEIII-500 MHz spectrometer (Bruker, Ettlingen, Germany) in acetone-**d*_6_*, CD_3_OD, and C_5_D_5_N using tetramethylsilane (TMS) as an internal reference standard. Chemical shifts were expressed as δ in ppm and the coupling constants (*J*) were given in Hz. High-resolution electrospray mass spectroscopy was performed on an Agilent G6230 TOF mass spectrometer (HR-ESI-MS) (Agilent Technologies, California, CA, USA) and a Waters Autospec Premier 776 mass spectrometer (HR-EI-MS) (Waters Technologies, Massachusetts, MA, USA). High-performance liquid chromatography (HPLC) was conducted on an Ultimate 3000 HPLC system (Dionex Co., Sunnyvale, CA, USA) equipped with an Ultimate 3000 pump and variable wavelength detector, as well as a semi-preparative YMC-Pack ODS-A column (250 × 10 mm, 5 µm, YMC, Kyoto, Japan). Column chromatography (CC) was conducted over silica gel (300–400 mesh, Qingdao Haiyang Co. Ltd., Qingdao, China). HPLC grade methanol and acetonitrile were purchased from Tedia Co. Inc, (Fairfield, OH, USA).

### 4.2. Plant Material

The fruits of *G. xanthochymus* were purchased from Xishuangbanna Prefecture, Yunnan Province, China and identified by Prof. Ying-hong Zhao, Xishuangbanna Prefecture National Medicine Research Institute. The voucher specimen was deposited in the herbarium of the School of Pharmacy, South-Central University for Nationalities.

### 4.3. Regents

MTT, Trizma^®^ base, glycine, SDS, acrylamide, N, N-Methylene bisacrylamide, and DMSO were purchased from Sigma-Aldrich (St. Louis, MO, USA). Dulbecco’s Modified Eagle Medium (DMEM), phosphate buffer saline (PBS), and penicillin-streptomycin solution were from Hyclone (Logan, UT, USA). Fetal bovine serum (FBS) was purchased from Gibco (Grand Island, NY, USA). Doxorubicin hydrochloride was purchased from Aladdin (Shanghai, China). Rabbit polyclonal antibodies against STAT3, phospho-STAT3 (Tyr705), VEGF, MMP-7, Cyclin D1, Mcl-1, Survivin, Bcl-XL, Bcl-2, β-actin, and HRP-conjugated goat anti-rabbit antibody were purchased from ABclonal (Woburn, MA, USA). WesternBright™ Sirius chemiluminescent detection reagent was from Advansta (Menlo Park, CA, USA). Stock solutions of compounds (50 mM) were prepared in DMSO and stored at 4 °C. The stock solutions were diluted with DMEM to working concentrations as working solutions.

### 4.4. Extraction and Isolation

The dried fruits of *G. xanthochymus* (6.18 kg) were powdered and extracted with 95% EtOH at room temperature three times (each time for 24 h) to obtain 2.94 kg of EtOH extract, and then successively partitioned with petroleum ether (P. E.), EtOAc, and *n*-BuOH to get petroleum ether extract 267 g, EtOAc extract 711 g, and. *n*-BuOH extract 460 g. The EtOAc extract (332 g) was chromatographed on a silica gel column (200–300 mesh) and eluted successively with petroleum ether/EtOAc gradient (19:1, 9:1, 7:3, 6:4, 1:1, 4:6, 3:7, 0:1) to obtain 10 fractions (Fr. 1–Fr. 10). Fr. 3 was subjected to octadecylsilane CC eluting with H_2_O-MeOH (7:3, 6:4, 1:1, 4:6, 3:7, 2:8, 0:1) to obtain 10 major fractions (Fr. 3.1–Fr. 3.10). Fr. 3.6 was subjected to the repeated silica gel column, ODS CC, and semi-preparative HPLC to give compound **3** (2.1 mg, *t_R_* = 48.0 min, 64% CH_3_CN). Fr. 3.7 was subjected to the repeated silica gel column, ODS CC, and semi-preparative HPLC to give **1** (13.1 mg, *t_R_* = 26.5 min, 78.5% CH_3_CN); **2** (32.8 mg, *t_R_* = 41.8 min, 80% CH_3_CN); **4** (4.7 mg, *t_R_* = 31.0 min, 79% CH_3_CN); and **13** (19.6 mg, *t_R_* = 33.1 min, 75% CH_3_CN). Fr. 3.9 was subjected to the repeated silica gel column and semi-preparative HPLC to produce **5** (12.9 mg, *t_R_* 23.9 min, 73% CH_3_CN); **6** (13.7 mg, *t_R_* = 25.5 min, 73% CH_3_CN); **11** (7.6 mg, *t_R_* 31.0 min, 83% CH_3_CN); **12** (9.3 mg; *t_R_* = 14.0 min 90% CH_3_CN); **14** (1.8 mg, *t_R_* = 45.2 min, 85% CH_3_CN); and **15** (3.1 mg, *t_R_* = 40.0 min, 83% CH_3_CN). Fr. 7 was subjected to a silica gel column (200–300 mesh) and eluted successively with CHCl_3_/MeOH gradient (500:1, 100:1, 20:1, 10:1, 7:3, 0:1) to obtain 14 fractions (Fr. 7.1–Fr. 7.14). Fr. 7.4 was subjected to the repeated octadecylsilane CC and semi-preparative HPLC to produce **9** (10.9 mg *t_R_* = 42.8 min, 50% CH_3_CN) and **10** (10.8 mg *t_R_* = 49.3 min, 50% CH_3_CN). Fr. 7.5 was subjected to repeated octadecylsilane CC and semi-preparative HPLC to produce **7** (2.7 mg, *t_R_* = 20.1 min, 30% CH_3_CN) and **8** (3.1 mg, *t_R_* = 20.5 min 30% CH_3_CN).

Garcixanthochymone F (**3**): yellow powder. ${\left[\alpha \right]}_{D}^{20}$ = +55.3° (*c* = 0.21, MeOH); UV (MeOH) λ_max_ nm (logε): 204 (4.12), 260 (3.94), 360 (3.70); ECD (c 3.4 × 10^−4^ M, MeOH) λ (θ): 224 (−9.75), 270 (+9.89); ^1^H- and ^13^C-NMR: see Table 1 and Table 2; HREIMS *m/z*: 616.3386 [M]^+^ (calculated for C_38_H_48_O_7_^+^, 616.3400).

Garcixanthochymone G (**5**): yellow powder. ${\left[\alpha \right]}_{D}^{20}$ = +28.5° (*c* = 0.87, MeOH); UV (MeOH) λ_max_ nm (logε): 202 (4.40), 262 (4.23), 308 (3.81), 370 (3.98); ECD (c 2.1 × 10^−4^ M, MeOH) λ (θ): 214 (+13.10), 266 (−5.90), 304 (+3.90); ^1^H- and ^13^C-NMR: see Table 1 and Table 2; HR-ESI-MS *m*/*z*: 641.3452 [M + Na]^+^ (calculated for C_38_H_50_O_7_Na^+^, 641.3454).

Garcixanthochymone H (**7**): yellow powder. ${\left[\alpha \right]}_{D}^{20}$ = +31.6° (*c* = 0.25, MeOH); UV (MeOH) λ_max_ nm (logε): 206 (4.17), 230 sh (4.01), 276 (4.09), 354 sh (3.67); ECD (c 6.0 × 10^−4^ M, MeOH) λ (θ): 223 (−25.89), 271 (+25.89); ^1^H- and ^13^C-NMR: see Table 1 and Table 2; HRESIMS *m/z*: 695.3766 [M + Na]^+^ (calculated for C_38_H_56_O_10_Na^+^, 695.3711).

Garcixanthochymone I (**8**): yellow powder. ${\left[\alpha \right]}_{D}^{20}$ = +17.8° (*c* = 0.33, MeOH); UV (MeOH) λ_max_ nm (logε): 204 (4.36), 265 (4.17), 361 (3.83); ECD (c 4.9 × 10^−4^ M, MeOH) λ (θ): 223 (−25.84), 272 (+27.25); ^1^H- and ^13^C-NMR: see Table 1 and Table 2; HRESIMS *m/z*: 695.3773 [M + Na]^+^ (calculated for C_38_H_56_O_10_Na^+^, 695.3771).

Garcixanthochymone J (**11**): yellow powder. ${\left[\alpha \right]}_{D}^{20}$ = +53.2° (*c* = 0.73, MeOH); UV (MeOH) λ_max_ nm (logε): 203 (4.58), 263 (4.29), 292 (4.24), 372 (4.09); ECD (c 4.9 × 10^−4^ M, MeOH) λ (θ): 206 (−2.71), 234 (−1.51), 259 (+4.52), 305 (+2.06); ^1^H- and ^13^C-NMR: see Table 1 and Table 2; HRESIMS *m/z*: 601.3528 [M + H]^+^ (calculated for C_38_H_49_O_6_^+^, 601.3529).

Garcixanthochymone K (**15**): yellow powder. ${\left[\alpha \right]}_{D}^{20}$ = −37.9° (*c* = 0.20, MeOH); UV (MeOH) λ_max_ nm (logε): 203 (4.48), 258 (4.23), 290 (4.17), 371 (4.03); ECD (c 1.6 × 10^−4^ M, MeOH) λ (θ): 204 (−4.13), 225 (+7.79), 259 (−11.46); ^1^H- and ^13^C-NMR: see Table 1 and Table 2; HRESIMS *m/z*: 623.3345 [M + Na]^+^ (calculated for C_38_H_48_O_6_Na^+^, 623.3349).

### 4.5. Cell Culture and Treatment

HepG2 (human hepatocellular carcinoma), SGC7901 (human gastric adenocarcinoma), A549 (human lung adenocarcinoma), and MCF-7 (human breast adenocarcinoma) cells were purchased from the cell bank of the Chinese Academy of Sciences (Shanghai, China). All cell lines were cultured in Dulbecco’s modified Eagle’s medium (DMEM) with 10% fetal bovine serum and 1% penicillin-streptomycin solution, grown at 37 °C in an atmosphere of 5% CO_2_, and subcultured every 2–3 days to maintain exponential growth.

### 4.6. MTT Assay

The anti-proliferative activity against four human tumor cell lines (SGC7901, A549, HepG2, and MCF-7) of isolated compounds was measured by MTT assay. The cells were cultured in 96-well plates at a concentration of 1 × 10^4^ cells per well in DMEM containing 10% FBS and 1% penicillin-streptomycin at 37 °C in 5% CO_2_ incubator. After 24 h, we washed the cells three times with PBS, then added 200 μL sample or doxorubicin solution in each well at a gradient of 50 μM, 25 μM, 12.5 μM, 6.25 μM, and 3.125 μM. After another 24 h, we removed the cell culture medium and washed the cells three times with PBS, then added 100 μL MTT (0.5 mg/mL) solution in each well and incubated for 4 h, then removed the supernatant and added 100 μL DMSO in each well, and read the absorbance at a wavelength of 492 nm using a Multiskan GO microplate reader (Thermo Fisher Scientific Inc. Waltham, MA, USA). The inhibitory rate of the compound (%) was calculated with the formula [A]test/[A]control × 100% ([A]test is the absorbance of the test group, with the cells incubated with sample solution; [A]control is the absorbance of the control group, with the cells incubated only with cell culture medium). The IC_50_ values was calculated according to the inhibition rate by using GraphPad Prism 6.

### 4.7. Docking Analysis

The 2.25 Å Mus musculus transcription factor STAT3B/DNA complex (Uniport: P42227) was downloaded from the RCSB Protein Data Bank (PDB id: 1BG1). The DNA chain was removed from the complex before docking analysis. The SH2 domain (580–670) were chosen as the docking sites according to Sethi et al. [44].

The protein was prepared by using Schrödinger software using Glide, Epik, OPLS3 force-field, Glide, LigPrep, and the Protein Preparation Wizard. The polar hydrogen and electric charges were added to the molecule and then the water molecules were removed from it, and finally, the protein structure was optimized. Relevant compounds were prepared via a ligand preparation workflow (Epik to generate possible protonation states at target pH 7.0 ± 2.0) and generating tautomers. The two compounds from the fruit of *G. xanthochymus* (**2** and **5**) as the ligands were prepared in Schrödinger Ligprep (Epik mode). The residues are 15 Å around the GLU638 as the docking pocket.

### 4.8. Western Blotting Assays

The whole cell extracts treated with compounds **2** and **5** were lysed in RIPA lysis buffer with 1 mM PMSF for 15 min. Next, the lysates were spun at 15,000 rpm for 10 min to remove insoluble material. We quantified the concentrations of the proteins and mixed with 5 × SDS loading buffer, then added this to a 10% SDS-PAGE gel. After electrophoresis, the proteins were electrotransferred to a 0.45 μm PVDF membrane (Millipore) then blocked with skim milk, and probed with various antibodies (1:1000) overnight at 4 °C. The membranes were washed 3 times, then exposed to HRP-conjugated secondary antibodies for 1 h, and finally examined by a chemilumiescence detector (Omega Lum G, Aplegen, CA, USA). The gray scanning analysis of blots was done by using Image J software, and the results were expressed as fold change relative to the control.

## 5. Conclusions

To uncover the natural compounds harboring anti-tumor activity in the fruits of *G. xanthochymus*, we isolated and identified six new PPAPs, named garcixanthochymones F–K (**3**, **5**, **7**, **8**, **11** and **15**), and nine known analogues, most of which possessed different degrees of anti-proliferative activity in tumor cells. Docking analysis indicated that compounds **2** and **5** could interact with the SH2 domain of STAT3 through hydrogen bonds and hydrophobic force. Morphological observation and Hoechst 33258 staining revealed that compounds **2** and **5** induced significant apoptosis in MCF-7 cells. Moreover, compounds **2** and **5** inhibited the phosphorylation of pSTAT3^Tyr705^ and the expression of various down-stream genes (Bcl-XL, Bcl-2, Mcl-1, Survivin, Cyclin D1, VEGF and MMP-7) of STAT3. Thus, the antitumor effects of compounds **2** and **5** were attributed to the suppression of STAT3 signaling, and we provided scientific evidence that compounds **2** and **5** could serve as potential agents for cancer therapy.

## Figures and Tables

**Table 1:** ^13^H NMR (500 MHz) data of compounds **3**, **5**, **7**–**8**, **11**, and **15**.

Position	3 ^a^	5 ^b^	7 ^c^	8 ^c^	11 ^b^	15 ^b^
7	1.52 m	2.40 m	1.82 m	1.83 m	1.91 m	1.56 m
8	2.33 d (14.5) 2.02 m	2.33 m 1.63 m	2.28 d (14.5) 2.20 m	2.44 d (14.0) 2.35 dd (14.5, 6.5)	2.42 dd (13.5, 4.0) 1.74 m	2.11 m
12	7.39 d (2.0)	8.18 d (2.0)	7.22 d (2.0)	7.23 d (2.0)	8.11 s	8.10 s
15	6.84 d (8.0)	7.29 d (8.0)	6.76 d (8.0)	6.77 d (8.0)	7.26 s	7.45 s
16	7.14 dd (8.0, 2.0)	7.88 dd (8.0, 2.0)	7.10 dd (8.0, 2.0)	7.11 dd (8.0, 2.0)		
17	2.63 dd (13.6, 8.0) 2.43 m	3.21 dd (14.0, 8.0) 2.48 dd (14.0, 7.5)	1.89 m	1.88 m	2.97 dd (13.5, 6.5) 2.86 dd (13.5, 6.5)	3.02 m
18	5.01 m	4.82 t (8.0)	1.43 m 1.08 m	1.44 m 1.10 m	5.20 br s	5.04 br s
20	1.57 s	1.34 s	1.16 s	1.17 s	1.85 s	1.37 s
21	1.56 s	1.35 s	1.17 s	1.18 s	1.55 s	1.78 s
22	0.97 s	0.95 s	1.02 s	1.02 s	0.85 s	1.15 s
23	1.14 s	1.18 s	1.14 s	1.13 s	1.21 s	1.27 s
24	2.75 m 1.29 m	2.21 m 1.75 m	2.16 m 1.44 m	2.05 dd (14.5, 5.5) 1.49 m	2.08 m 1.79 m	2.09 m 1.97 m
25	5.01 m	5.18 t (6.5)	3.18 d (10.5)	3.12 m	4.92 br s	5.01 br s
26						
27	1.68 s	1.58 s	1.21 s	1.23 s	1.51 s	1.64 s
28	1.69 s	1.62 s	1.22 s	1.20 s	1.51 s	1.51 s
29	3.01 dd (14.0, 4.0) 1.19 dd (14.0, 9.5)	2.32 m 2.03 m	3.13 dd (14.0, 3.5) 1.09 m	3.12 m 1.09 m	2.36 dd (14.0, 9.5) 2.15 dd (14.0, 2.5)	2.39 m 2.13 m
30	2.10 m	2.96 m	1.27 m	1.26 m	2.65 m	
31						2.48 m
32	1.30 s	4.92 s 4.88 s	1.29 s	1.30 s	4.44 s 4.38 s	2.23 m 1.91 m
33	0.91 s	1.67 s	0.95 s	0.95 s	1.63 s	2.29 m 2.21 m
34	2.80 m 2.73 m	1.59 m	1.52 m 1.09 m	1.52 m 1.12 m	1.73 m 1.60 m	
35		1.94 m	1.80 m 1.42 m	1.80 m 1.42 m	1.91 m	4.80 br s 4.90 br s
36						1.65 s
37	6.09 s 5.88 s	4.82 s 4.76 s	1.22 s	1.20 s	4.77 s	1.01 s
38	1.89 s	1.70 s	1.22 s	1.22 s	1.71 s	1.20 s

Assignments based on DEPT, HSQC, and HMBC experiments. Chemical shifts in ppm, *J* in Hz. ^a^ Recorded in acetone-*d*_6_; ^b^ recorded in pyridine-*d*_5_; ^c^ recorded in CD_3_OD.

**Table 2:** ^13^C NMR (125 MHz) data of compounds **3**, **5**, **7**–**8**, **11**, and **15**.

Position	3 ^a^	5 ^b^	7 ^c^	8 ^c^	11 ^b^	15 ^b^
1	51.4	62.5	53.0	53.3	57.9	66.3
2	170.9	195.2	174.0	174.4	176.8	193.1
3	126.3	119.9	127.1	127.3	119.8	119.2
4	194.3	174.5	196.8	197.1	191.9	177.0
5	69.0	70.2	69.6	69.8	74.4	63.6
6	46.7	47.4	47.7	48.8	49.2	48.8
7	47.0	42.0	43.5	44.7	42.9	47.3
8	39.9	44.2	40.3	46.1	44.3	38.2
9	206.9	207.6	208.2	208.3	207.9	207.7
10	192.3	191.7	194.8	194.8	172.4	172.8
11	131.3	131.0	131.0	131.2	118.7	118.0
12	115.9	117.1	116.7	116.7	110.0	109.6
13	146.0	147.9	146.8	146.8	149.8	146.9
14	151.3	153.8	153.1	152.9	154.8	155.9
15	115.6	116.2	116.0	116.0	104.5	104.5
16	123.6	125.2	124.4	124.3	147.6	150.6
17	26.4	26.6	22.8	22.5	26.2	27.4
18	121.6	93.7	39.8	39.7	122.0	119.9
19	134.2	71.5	71.9	72.0	133.6	135.3
20	26.4	25.9	29.2	29.2	26.4	26.2
21	18.3	26.1	29.2	29.3	18.8	18.8
22	27.0	16.4	27.2	27.1	16.4	26.9
23	22.8	23.4	23.1	22.8	23.7	22.4
24	29.8	29.4	33.5	37.4	28.7	30.1
25	126.4	123.5	78.6	83.2	123.5	124.6
26	133.3	133.8	74.4	74.5	134.0	135.3
27	26.2	26.3	24.6	24.6	26.1	26.3
28	18.6	18.2	25.9	25.8	18.0	18.2
29	29.3	37.1	29.2	28.9	36.5	40.0
30	38.9	44.0	45.0	45.1	44.4	41.5
31	86.0	148.1	89.2	89.4	147.8	49.4
32	22.2	114.6	21.7	21.7	113.0	29.4
33	29.0	18.3	29.1	29.1	18.2	31.8
34	39.7	32.4	26.6	26.6	32.6	146.9
35	199.8	36.4	42.8	42.8	35.9	109.6
36	145.5	146.6	71.4	71.4	146.1	24.3
37	125.5	110.4	29.3	29.3	111.0	24.7
38	17.9	23.1	29.7	29.7	22.9111.0	25.7

^a^ Recorded in acetone-*d*_6_; ^b^ recorded in pyridine-*d*_5_; ^c^ recorded in CD_3_OD.

**Table 3:** Anti-proliferative activity of isolated compounds ^b^.

Compound	IC_50_ in μmol·L^−1^			
	SGC7901	A549	Hep G2	MCF-7
Doxorubicin ^a^	7.54 ± 1.11	14.03 ± 0.21	6.52 ± 0.13	4.40 ± 1.17
**1**	13.96 ± 2.88	6.06 ± 0.04	9.60 ± 0.39	27.33 ± 0.22
**2**	11.41 ± 1.22	7.06 ± 0.20	9.55 ± 0.63	29.41 ± 0.85
**3**	11.00 ± 1.96	10.66 ± 1.85	14.45 ± 1.06	25.85 ± 4.07
**4**	23.57 ± 1.06	11.95 ± 0.01	17.84 ± 0.06	25.27 ± 1.53
**5**	14.22 ± 0.47	8.29 ± 0.31	9.22 ± 0.81	21.77 ± 0.46
**6**	15.26 ± 2.26	4.55 ± 0.34	8.62 ± 0.32	16.93 ± 0.41
**7**	>50	>50	>50	>50
**8**	>50	>50	>50	>50
**9**	19.08 ± 3.71	21.44 ± 2.12	19.09 ± 1.97	22.29 ± 0.12
**10**	17.58 ± 0.92	19.28 ± 3.58	18.23 ± 4.18	25.93 ± 0.76
**11**	>50	>50	>50	>50
**12**	2.83 ± 0.66	0.89 ± 0.26	4.91 ± 1.61	3.28 ± 2.17
**13**	18.53 ± 6.46	9.17 ± 2.10	>50	>50
**14**	36.98 ± 9.72	32.76 ± 0.41	>50	>50
**15**	>50	>50	>50	>50

^a^ Doxorubicin was used as positive control. ^b^ The results are shown as IC_50_ ± SD in µmol·L^−1^ of 3 independent experiments.

## Data Availability

The data presented in this study are available on request from the corresponding authors.

## Supplementary Materials

The following are available online at https://www.mdpi.com/article/10.3390/ijms221910365/s1.

## References

[1] Bray F., Jemal A., Grey N., Ferlay J., Forman D. (2012). Global cancer transitions according to the Human Development Index (2008–2030): A population-based study. Lancet Oncol..

[2] Lee Y.T., Tan Y.J., Oon C.E. (2018). Molecular targeted therapy: Treating cancer with specificity. Eur. J. Pharm..

[3] Darnell J.E. (1997). STATs and gene regulation. Science.

[4] Bowman T., Garcia R., Turkson J., Jove R. (2000). STATs in oncogenesis. Oncogene.

[5] Sriuranpong V., Park J.I., Amornphimoltham P., Patel V., Nelkin B.D., Gutkind J.S. (2003). Epidermal growth factor receptor-independent constitutive activation of STAT3 in head and neck squamous cell carcinoma is mediated by the autocrine/paracrine stimulation of the interleukin 6/gp130 cytokine system. Cancer Res.

[6] Bromberg J.F., Wrzeszczynska M.H., Devgan G., Zhao Y., Pestell R.G., Albanese C., Darnell J.E. (1999). Stat3 as an oncogene. Cell.

[7] Zhang X., Yue P., Fletcher S., Zhao W., Gunning P.T., Turkson J. (2010). A novel small-molecule disrupts Stat3 SH2 domain-phosphotyrosine interactions and Stat3-dependent tumor processes. Biochem. Pharm..

[8] You Z., Xu D., Ji J., Guo W., Zhu W., He J. (2012). JAK/STAT signal pathway activation promotes progression and survival of human oesophageal squamous cell carcinoma. Clin. Transl. Oncol..

[9] Koskela H.L., Eldfors S., Ellonen P., van Adrichem A.J., Kuusanmäki H., Andersson E.I., Lagström S., Clemente M.J., Olson T., Jalkanen S.E. (2012). Somatic STAT3 mutations in large granular lymphocytic leukemia. N. Engl. J. Med..

[10] Hsieh F.C., Cheng G., Lin J. (2005). Evaluation of potential Stat3-regulated genes in human breast cancer. Biochem. Biophys. Res. Commun..

[11] Wang Z., Hui C., Xie Y. (2021). Natural STAT3 inhibitors: A mini perspective. Bioorg. Chem..

[12] Flora Reipublicae Popularis Sinicae. http://www.iplant.cn/info/Garcinia%20xanthochymus?t=z.

[13] Liu B., Zhang X., Bussmann R.W., Hart R.H., Li P., Bai Y., Long C. (2017). *Garcinia* in Southern China: Ethnobotany, Management, and Niche Modeling. Econ. Bot..

[14] Yapwattanaphun C., Subhadrabandhu S., Sugiura A., Yonemori K., Utsunomiya N. (2002). Utilization of some *garcinia* species in thailand. Acta Hortic..

[15] Wu S.B., Long C., Kennelly E.J. (2014). Structural diversity and bioactivities of natural benzophenones. Nat. Prod. Rep..

[16] Kumar S., Sharma S., Chattopadhyay S.K. (2013). The potential health benefit of polyisoprenylated benzophenones from *Garcinia* and related genera: Ethnobotanical and therapeutic importance. Fitoterapia.

[17] Che Hassan N.K.N., Taher M., Susanti D. (2018). Phytochemical constituents and pharmacological properties of *Garcinia xanthochymus*—A review. Biomed. Pharm..

[18] Ciochina R., Grossman R.B. (2006). Polycyclic polyprenylated acylphloroglucinols. Chem. Rev..

[19] Yang H., Figueroa M., To S., Baggett S., Jiang B., Basile M.J., Weinstein I.B., Kennelly E.J. (2010). Benzophenones and biflavonoids from *Garcinia livingstonei* fruits. J. Agric. Food Chem..

[20] Xu J., Jin S., Gan F., Xiong H., Mei Z., Chen Y., Yang G. (2020). Polycyclic polyprenylated acylphloroglucinols from *Garcinia xanthochymus* fruits exhibit antitumor effects through inhibition of the STAT3 signaling pathway. Food Funct..

[21] Jin S., Shi K., Liu L., Chen Y., Yang G. (2019). Xanthones from the Bark of *Garcinia xanthochymus* and the Mechanism of Induced Apoptosis in Human Hepatocellular Carcinoma HepG2 Cells via the Mitochondrial Pathway. Int. J. Mol. Sci..

[22] Chen Y., Gan F., Jin S., Liu H., Wu S., Yang W., Yang G. (2017). Adamantyl derivatives and rearranged benzophenones from *Garcinia xanthochymus* fruits. RSC Adv..

[23] Roux D., Hadi H.A., Thoret S., Guénard D., Thoison O., Païs M., Sévenet T. (2000). Structure-activity relationship of polyisoprenyl benzophenones from *Garcinia pyrifera* on the tubulin/microtubule system. J. Nat. Prod..

[24] Phang Y., Wang X., Lu Y., Fu W., Zheng C., Xu H. (2020). Bicyclic polyprenylated acylphloroglucinols and their derivatives: Structural modification, structure-activity relationship, biological activity and mechanism of action. Eur. J. Med. Chem..

[25] Socolsky C., Plietker B. (2015). Total synthesis and absolute configuration assignment of MRSA active garcinol and isogarcinol. Chemistry.

[26] Xia Z.X., Zhang D.D., Liang S., Lao Y.Z., Zhang H., Tan H.S., Chen S.L., Wang X.H., Xu H.X. (2012). Bioassay-guided isolation of prenylated xanthones and polycyclic acylphloroglucinols from the leaves of *Garcinia nujiangensis*. J. Nat. Prod..

[27] Marti G., Eparvier V., Moretti C., Susplugas S., Prado S., Grellier P., Retailleau P., Gueritte F., Litaudon M. (2009). Antiplasmodial benzophenones from the trunk latex of *Moronobea coccinea* (Clusiaceae). Phytochemistry.

[28] Tian W.-J., Qiu Y.-Q., Jin X.-J., Chen H.-F., Yao X.-J., Dai Y., Yao X.-S. (2016). Hypersampsones S–W, new polycyclic polyprenylated acylphloroglucinols from *Hypericum sampsonii*. RSC Adv..

[29] Masullo M., Bassarello C., Bifulco G., Piacente S. (2010). Polyisoprenylated benzophenone derivatives from the fruits of *Garcinia cambogia* and their absolute configuration by quantum chemical circular dichroism calculations. Tetrahedron.

[30] Yang X.W., Grossman R.B., Xu G. (2018). Research Progress of Polycyclic Polyprenylated Acylphloroglucinols. Chem. Rev..

[31] Baggett S., Protiva P., Mazzola E.P., Yang H., Ressler E.T., Basile M.J., Weinstein I.B., Kennelly E.J. (2005). Bioactive benzophenones from *Garcinia xanthochymus* fruits. J. Nat. Prod..

[32] Nugroho A.E., Nakamura H., Inoue D., Hirasawa Y., Wong C.P., Kaneda T., Hadi A.H.A., Morita H. (2018). Polyisoprenylated Acylphloroglucinols from *Garcinia nervosa*. Nat. Prod. Commun..

[33] Liu X., Yu T., Gao X.M., Zhou Y., Qiao C.F., Peng Y., Chen S.L., Luo K.Q., Xu H.X. (2010). Apoptotic effects of polyprenylated benzoylphloroglucinol derivatives from the twigs of *Garcinia multiflora*. J. Nat. Prod..

[34] Fu W., Wu M., Zhu L., Lao Y., Wang L., Tan H., Yuan Q., Xu H. (2015). Prenylated benzoylphloroglucinols and biphenyl derivatives from the leaves of *Garcinia multiflora* Champ. RSC Adv..

[35] Marti G., Eparvier V., Moretti C., Prado S., Grellier P., Hue N., Thoison O., Delpech B., Gueritte F., Litaudon M. (2010). Antiplasmodial benzophenone derivatives from the root barks of *Symphonia globulifera* (Clusiaceae). Phytochemistry.

[36] Yang X.W., Li M.M., Liu X., Ferreira D., Ding Y., Zhang J.J., Liao Y., Qin H.B., Xu G. (2015). Polycyclic Polyprenylated Acylphloroglucinol Congeners Possessing Diverse Structures from *Hypericum henryi*. J. Nat. Prod..

[37] Behera A.K., Swamy M.M., Natesh N., Kundu T.K. (2016). Garcinol and Its Role in Chronic Diseases. Adv. Exp. Med. Biol..

[38] Parasramka M.A., Gupta S.V. (2012). Synergistic effect of garcinol and curcumin on antiproliferative and apoptotic activity in pancreatic cancer cells. J. Oncol..

[39] Reis F.H., Pardo-Andreu G.L., Nunez-Figueredo Y., Cuesta-Rubio O., Marin-Prida J., Uyemura S.A., Curti C., Alberici L.C. (2014). Clusianone, a naturally occurring nemorosone regioisomer, uncouples rat liver mitochondria and induces HepG2 cell death. Chem. Biol. Interact.

[40] Liu C., Ho P.C., Wong F.C., Sethi G., Wang L.Z., Goh B.C. (2015). Garcinol: Current status of its anti-oxidative, anti-inflammatory and anti-cancer effects. Cancer Lett..

[41] Avalle L., Camporeale A., Camperi A., Poli V. (2017). STAT3 in cancer: A double edged sword. Cytokine.

[42] Huang M., Chen Z., Zhang L., Huang Z., Chen Y., Xu J., Zhang J., Shu X. (2016). Screening and biological evaluation of a novel STAT3 signaling pathway inhibitor against cancer. Bioorg. Med. Chem. Lett..

[43] Siveen K.S., Sikka S., Surana R., Dai X., Zhang J., Kumar A.P., Tan B.K., Sethi G., Bishayee A. (2014). Targeting the STAT3 signaling pathway in cancer: Role of synthetic and natural inhibitors. Biochim. Biophys. Acta.

[44] Sethi G., Chatterjee S., Rajendran P., Li F., Shanmugam M.K., Wong K.F., Kumar A.P., Senapati P., Behera A.K., Hui K.M. (2014). Inhibition of STAT3 dimerization and acetylation by garcinol suppresses the growth of human hepatocellular carcinoma *in vitro* and *in vivo*. Mol. Cancer.

