# Supplementary material for: Discovery of Novel Polycyclic Polyprenylated Acylphloroglucinols from the Fruits of Garcinia xanthochymus as Antitumor Agents by Suppressing the STAT3 Signaling
Source: Int J Mol Sci. 2021 Sep 26;22(19):10365. doi: 10.3390/ijms221910365 (PMC8508737; doi:10.3390/ijms221910365)
Supplement: Supplementary file 1 [file ijms-22-10365-s001.zip › ijms-1382381-supplementary.pdf]

Supplementary Information

Discovery of Novel Polycyclic Polyprenylated Acylphloroglucinols from the Fruits of *Garcinia xanthochymus* as Antitumor Agents by Suppressing the STAT3 signaling

Shan Jin [1,‡], Wen Wang [1,‡], Fei Gan [2], Wenli Xie [1], Jing Xu [1], Yu Chen [2], Zhinan Mei [1,*], Guangzhong Yang [1,*]

[1] School of Pharmaceutical Sciences, South-Central University for Nationalities, Wuhan 430074, P. R. China

[2] College of Chemistry and Material Sciences, South-Central University for Nationalities, Wuhan 430074, P. R. China

*Corresponding authors. Tel./fax: +86 27 6784 1196
E-mail address: meizhinan@163.com (Z. Mei); yanggz888@126.com (G. Yang)

‡These authors contributed equally to this work.

Contents

Figure S1. ^{1}H NMR spectrum of compound **3**......5
Figure S2. ^{13}C NMR spectrum of compound **3**......5
Figure S3. ^{1}H ^{1}HCOSY spectrum of compound **3**......6
Figure S4. HSQC spectrum of compound **3**......6
Figure S5. HMBC spectrum of compound **3**......7
Figure S6. ROESY spectrum of compound **3**......7
Figure S7. HR-EI-MS spectrum of compound **3**......8
Figure S8. CD spectrum of compound **3**......8
Figure S9. UV spectrum of compound **3**......9
Figure S10. ^{1}H NMR spectrum of compound **5**......9
Figure S11. ^{13}C NMR spectrum of compound **5**......10
Figure S12. ^{1}H ^{1}HCOSY spectrum of compound **5**......10
Figure S13. HSQC spectrum of compound **5**......11
Figure S14. HMBC spectrum of compound **5**......11
Figure S15. ROESY spectrum of compound **5**......12
Figure S16. HR-ESI-MS spectrum of compound **5**......12
Figure S17. CD spectrum of compound **5**......13
Figure S18. UV spectrum of compound **5**......13
Figure S19. ^{1}H NMR spectrum of compound **7**......14
Figure S20. ^{13}C NMR spectrum of compound **7**......14
Figure S21. ^{1}H-^{1}HCOSY spectrum of compound **7**......15
Figure S22. HSQC spectrum of compound **7**......15
Figure S23. HMBC spectrum of compound **7**......16
Figure S24. ROESY spectrum of compound **7**......16
Figure S25. HR-ESI-MS spectrum of compound **7**......17
Figure S26. CD spectrum of compound **7**......17
Figure S27. UV spectrum of compound **7**......18
Figure S28. ^{1}H NMR spectrum of compound **8**......18
Figure S29. ^{13}C NMR spectrum of compound **8**......19
Figure S30.^{1}H -^{1}HCOSY spectrum of compound **8**......19
Figure S31. HSQC spectrum of compound **8**......20
Figure S32. HMBC spectrum of compound **8**......20
Figure S33. ROESY spectrum of compound **8**......21
Figure S34. HR-ESI-MS spectrum of compound **8**......21
Figure S35. CD spectrum of compound **8**......22
Figure S36. UV spectrum of compound **8**......22
Figure S37. ^{1}H NMR spectrum of compound **11**......23
Figure S38. ^{13}C NMR spectrum of compound **11**......23
Figure S39. ^{1}H -^{1}H COSY spectrum of compound **11**......24
Figure S40. HSQC spectrum of compound **11**......24
Figure S41. HMBC spectrum of compound **11**......25
Figure S42. ROESY spectrum of compound **11**......25

Figure S43. HR-ESI-MS spectrum of compound **11** 26
Figure S44. CD spectrum of compound **11** 26
Figure S45. UV spectrum of compound **11** 27
Figure S46. ^{1}H NMR spectrum of compound **15** 27
Figure S47. ^{13}C NMR spectrum of compound **15** 28
Figure S48.^{1}H -^{1}HCOSY spectrum of compound **15** 28
Figure S49. HSQC spectrum of compound **15** 29
Figure S50. HMBC spectrum of compound **15** 29
Figure S51. ROESY spectrum of compound **15** 30
Figure S52. HR-ESI-MS spectrum of compound **15** 30
Figure S53. CD spectrum of compound **15** 31
Figure S54. UV spectrum of compound **15** 31
Figure S55. Chemical structure of compound **3**. 32
Figure S56. Chemical structure of compound **5** 33
Figure S57. Chemical structure of compound **7** 33
Figure S58. Chemical structure of compound **11** 34
Figure S59. Chemical structure of compound **15** 35
Figure S60. DP4+ probability for compounds **3a** and **3b** based on its ^{1}H and ^{13}C-NMR date. 144
Figure S61. DP4+ probability for compounds **7a** and **7b** based on its ^{1}H and ^{13}C-NMR date. 146
Figure S62. DP4+ probability for compounds **15a**,**15b**,**15c** and **15d** based on its ^{1}H and ^{13}C-NMR date. 148
Figure S63. Calculated ECD spectra of compound **5** was compared with the experimental. 151
Figure S64. Calculated ECD spectra of compound **11** was compared with the experimental. 151
Figure S65. Calculated ECD spectra of compound **15** was compared with the experimental. 152
Table S1. Energies of configurations **3-15** 38
Table S2. Standard orientations of configurations **3-15** 41
Table S3. Experimental and computed chemical shifts of **3**, **3a** and **3b**. 139
Table S4. Experimental and computed chemical shifts of **7**, **7a** and **7b**. 140
Table S5. Experimental and computed chemical shifts of **15**,**15a**,**15b**,**15c** and **15d**. 141
Table S6. Statistics of Ordinary Least Squares Linear Regression (OLS-LR) of experimental and computed ^{13}C-NMR chemical shifts. 142
Table S7. ^{1}H-NMR and ^{13}C-NMR of compounds **1-2** (in pyridine-d_5) 153
Table S8. ^{1}H-NMR and ^{13}C-NMR of compound **4** (in pyridine-d_5) 154
Table S9. ^{1}H-NMR and ^{13}C-NMR of compound **6** (in pyridine-d_5) 154
Table S10. ^{1}H-NMR and ^{13}C-NMR of compound **9** and **10** (in pyridine-d_5) 154
Table S11. ^{1}H-NMR and ^{13}C-NMR of compound **12** (in pyridine-d_5) 156
Table S12. ^{1}H-NMR and ^{13}C-NMR of compound **13** (in pyridine-d_5) 156
Table S13. ^{1}H-NMR and ^{13}C-NMR of compound **14** (in pyridine-d_5) 157
1. Computational methods 32
1.1 Conformational analysis 32
1.2 NMR calculation 35
1.3 ECD calculation 36
1.4 References 36
2. Energies and Coordinates 38

2.1 Energies at B3LYP theory level..38
2.2 Coordinates at B3LYP theory level 41
3. Experimental and Computed NMR Chemical Shifts 138
3.1 13C- and 1H-NMR chemical shifts..138
3.2 DP4+ analysis 143
4.Experimental and calculated ECD spectra 151

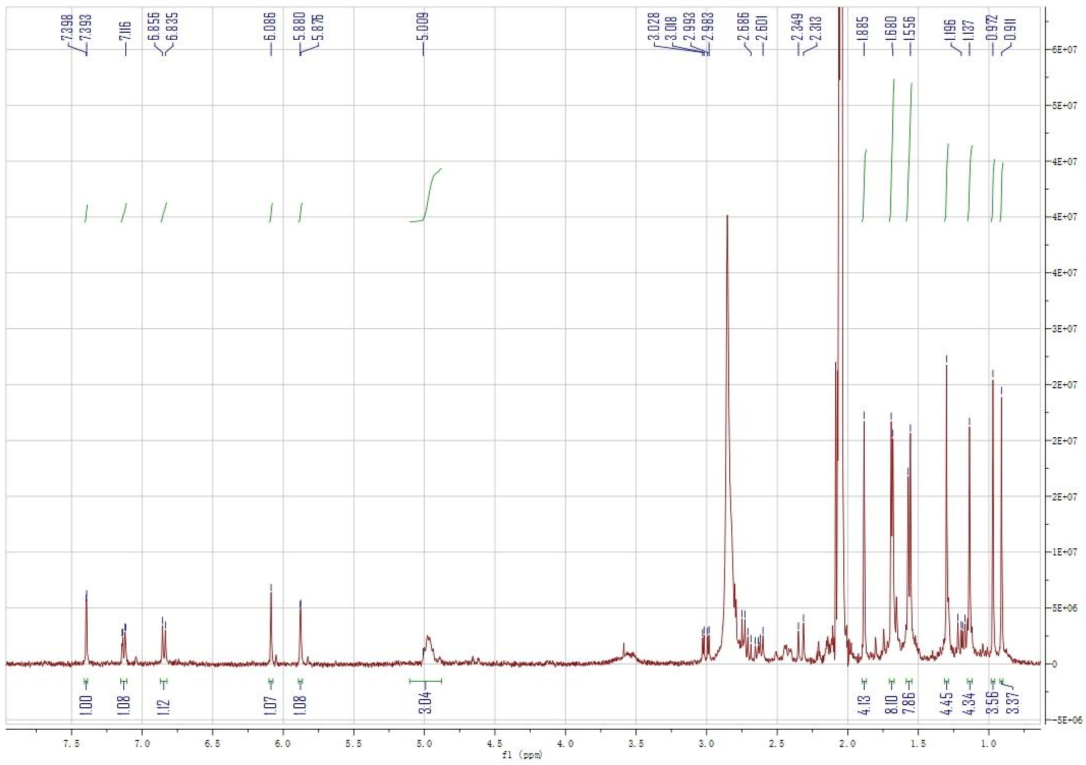

Figure S1. ^{1}H NMR spectrum of compound **3**

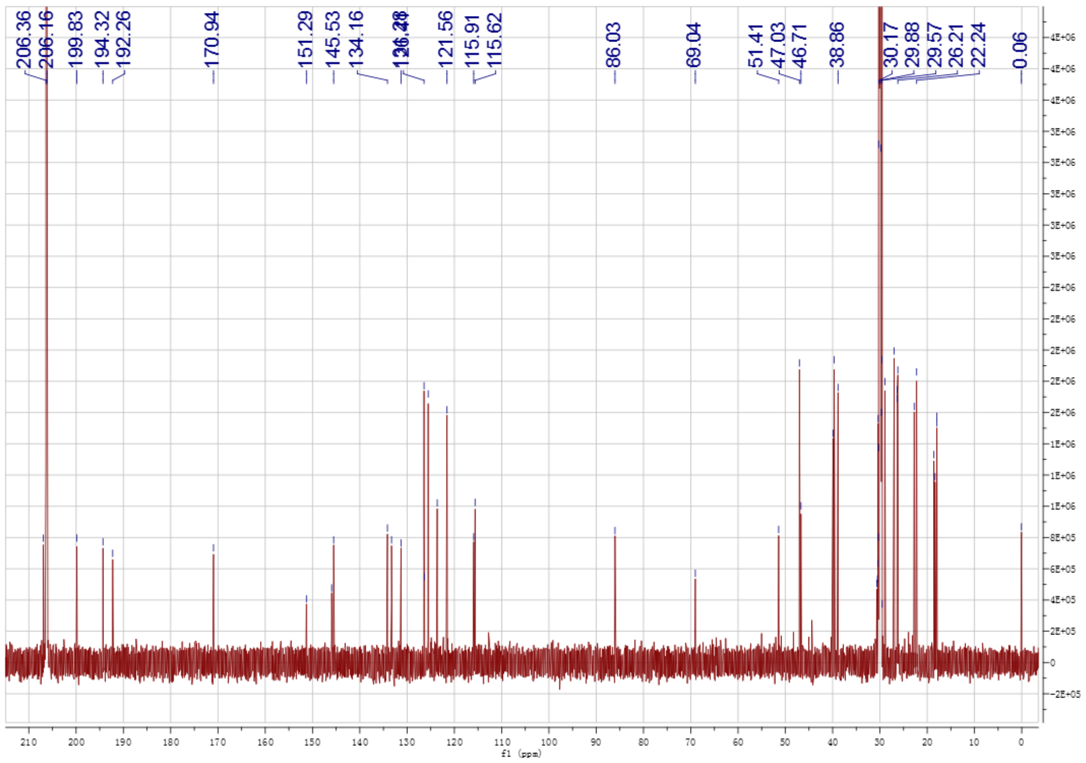

Figure S2. ^{13}C NMR spectrum of compound **3**

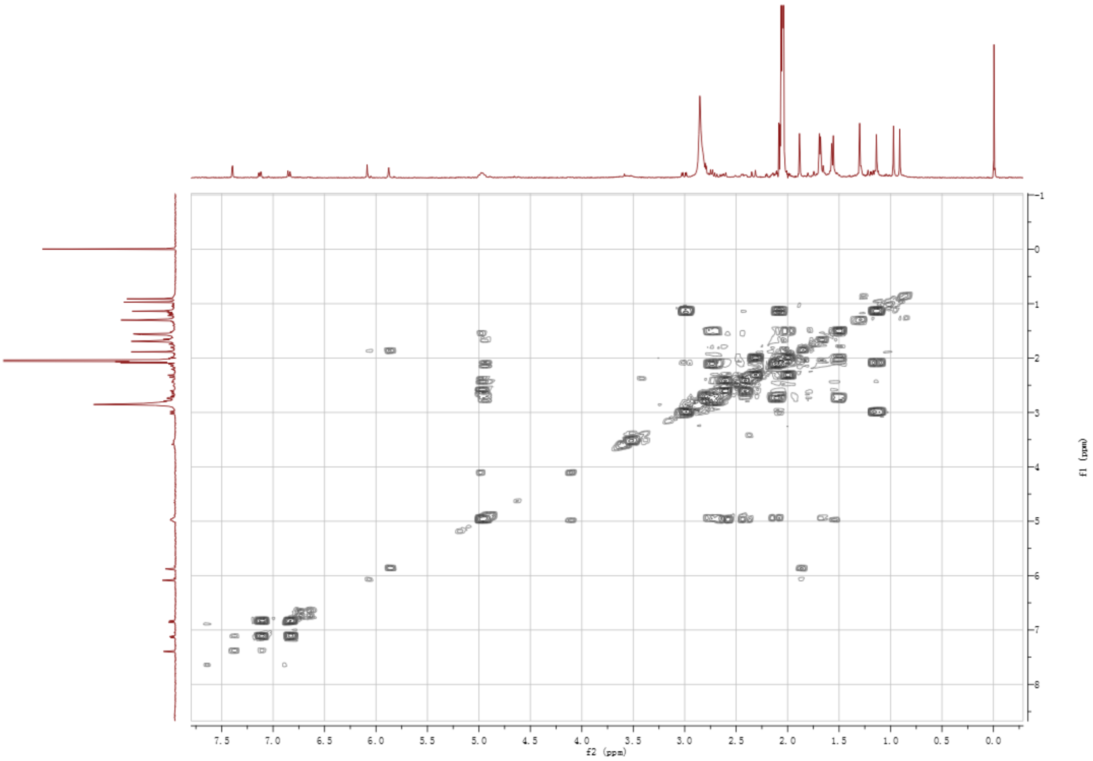

Figure S3. ^{1}H ^{1}HCOSY spectrum of compound **3**

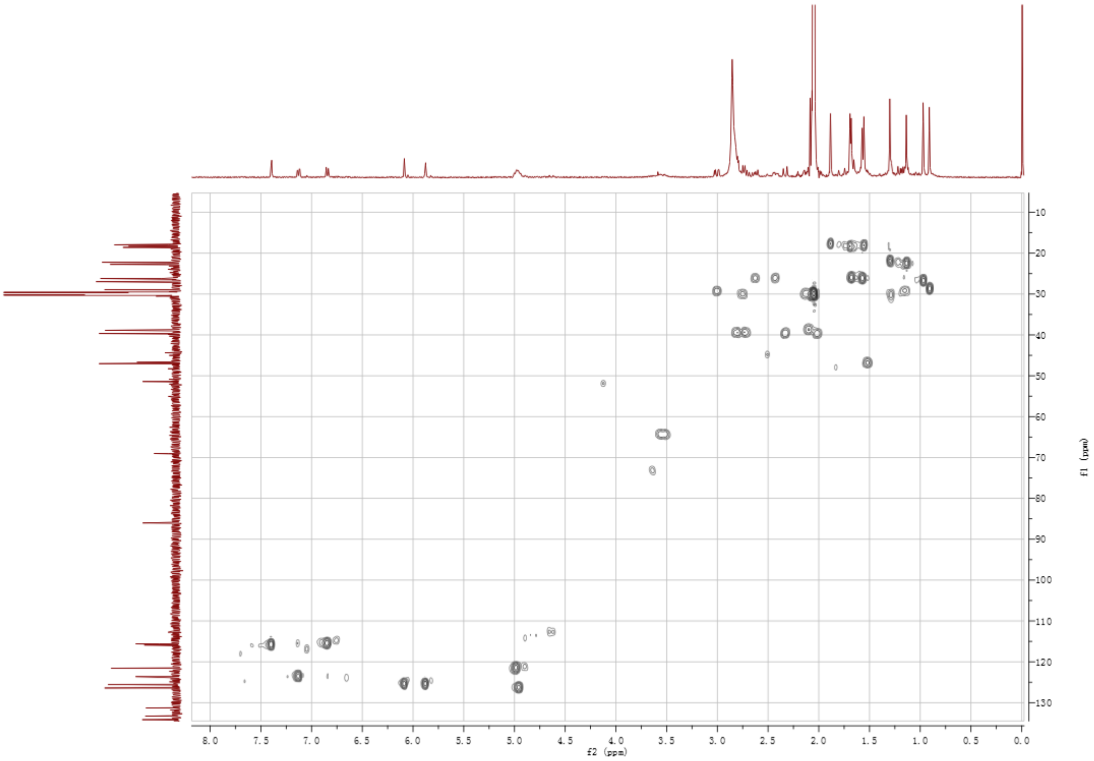

Figure S4. HSQC spectrum of compound **3**

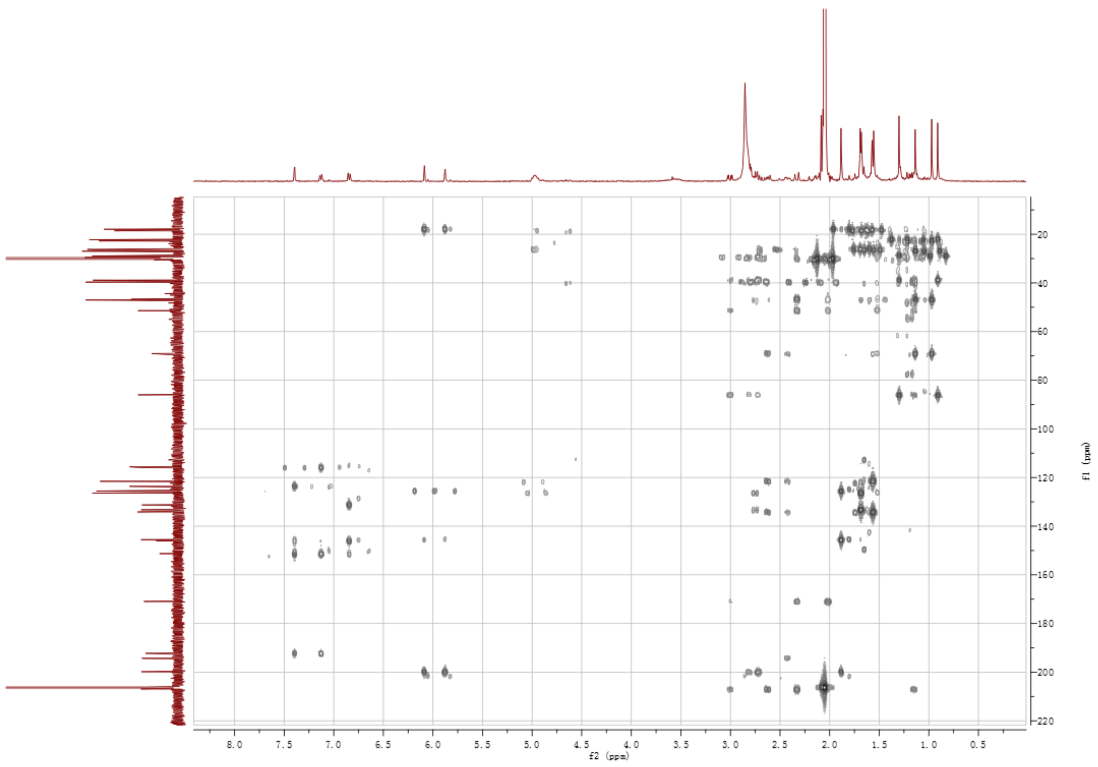

Figure S5. HMBC spectrum of compound **3**

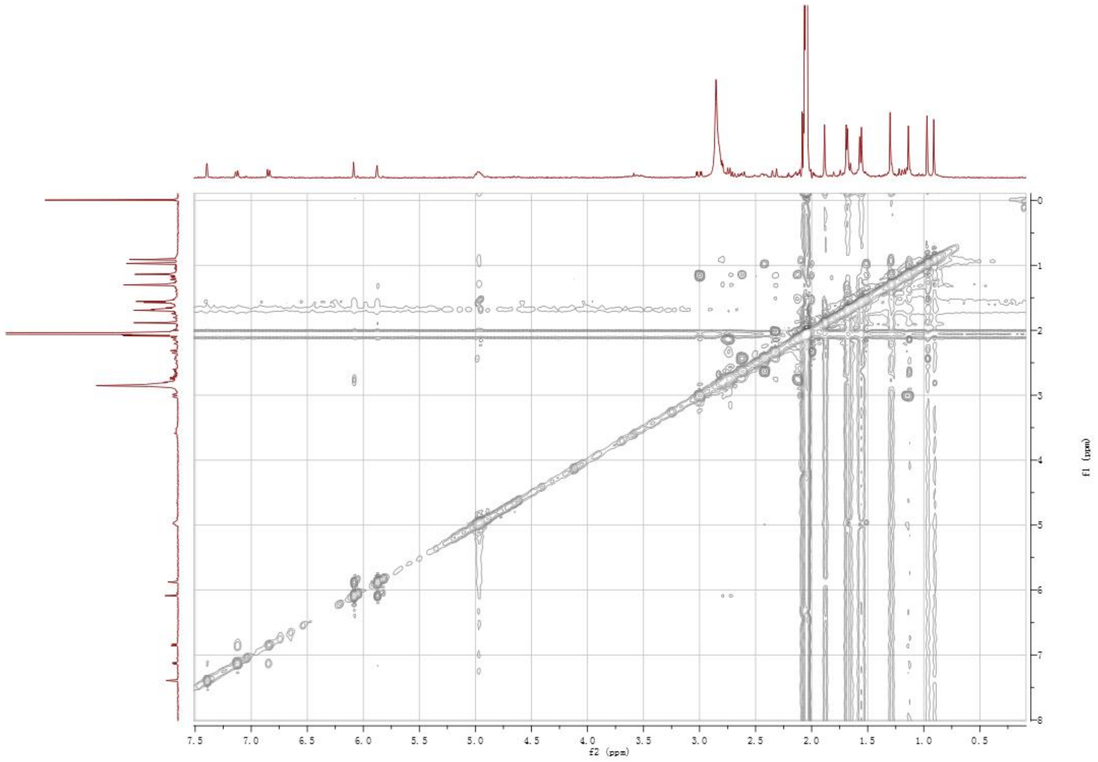

Figure S6. ROESY spectrum of compound **3**

Monoisotopic Mass, Odd and Even Electron Ions
25 formula(e) evaluated with 1 results within limits (up to 51 closest results for each mass)
Elements Used:
C: 0-200   H: 0-400   O: 6-8

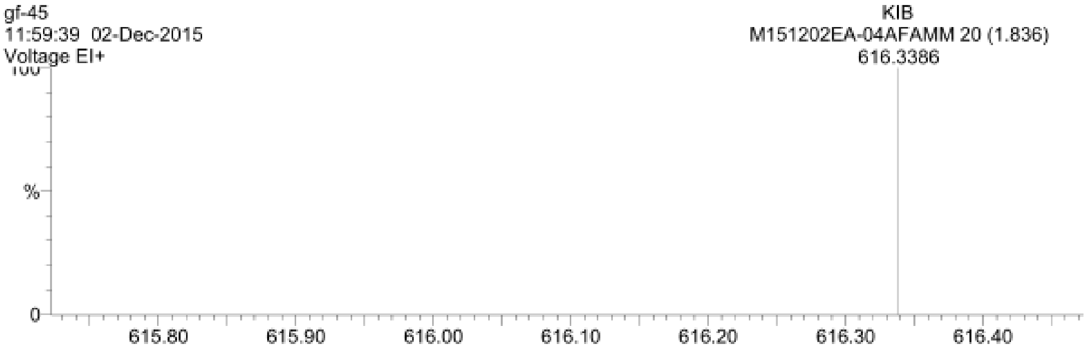

Minimum:				-10.0		
Maximum:		200.0	10.0	120.0		
Mass	Calc. Mass	mDa	PPM	DBE	i-FIT	Formula
616.3386	616.3400	-1.4	-2.3	15.0	5546364.5	C38 H48 O7

Figure S7. HR-EI-MS spectrum of compound **3**

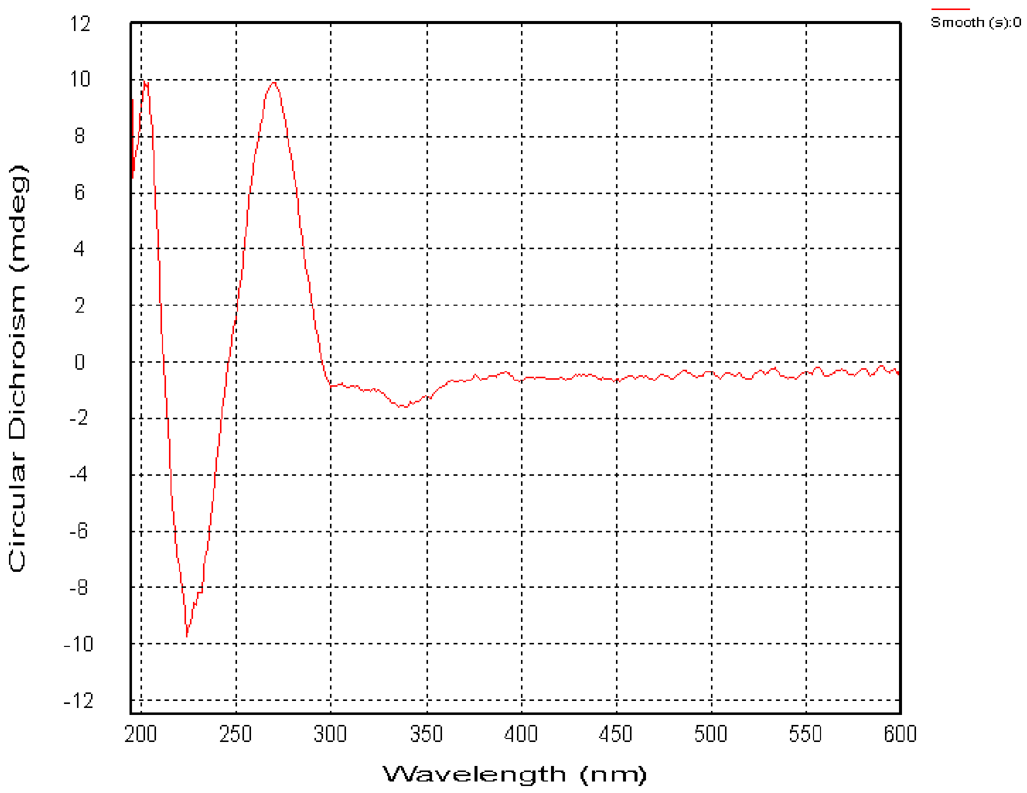

Figure S8. CD spectrum of compound **3**

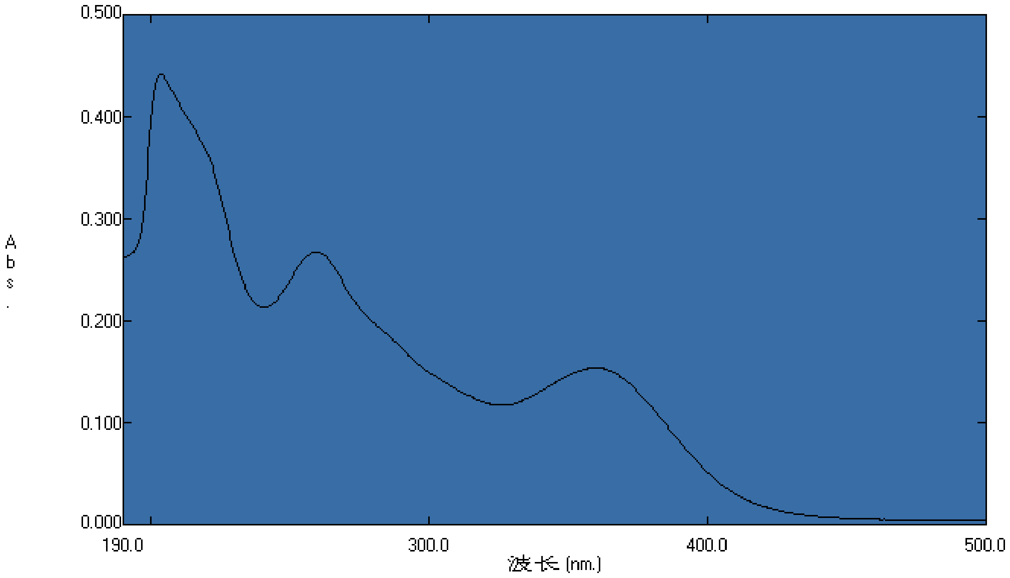

Figure S9. UV spectrum of compound **3**

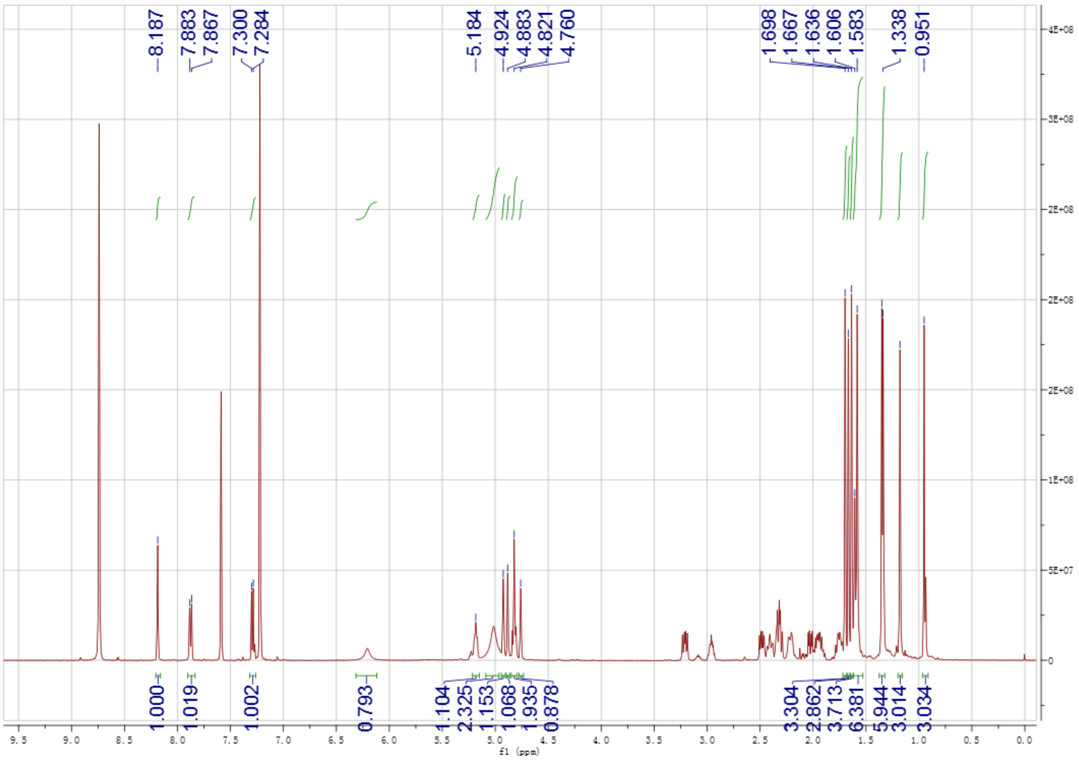

Figure S10. ^{1}H NMR spectrum of compound **5**

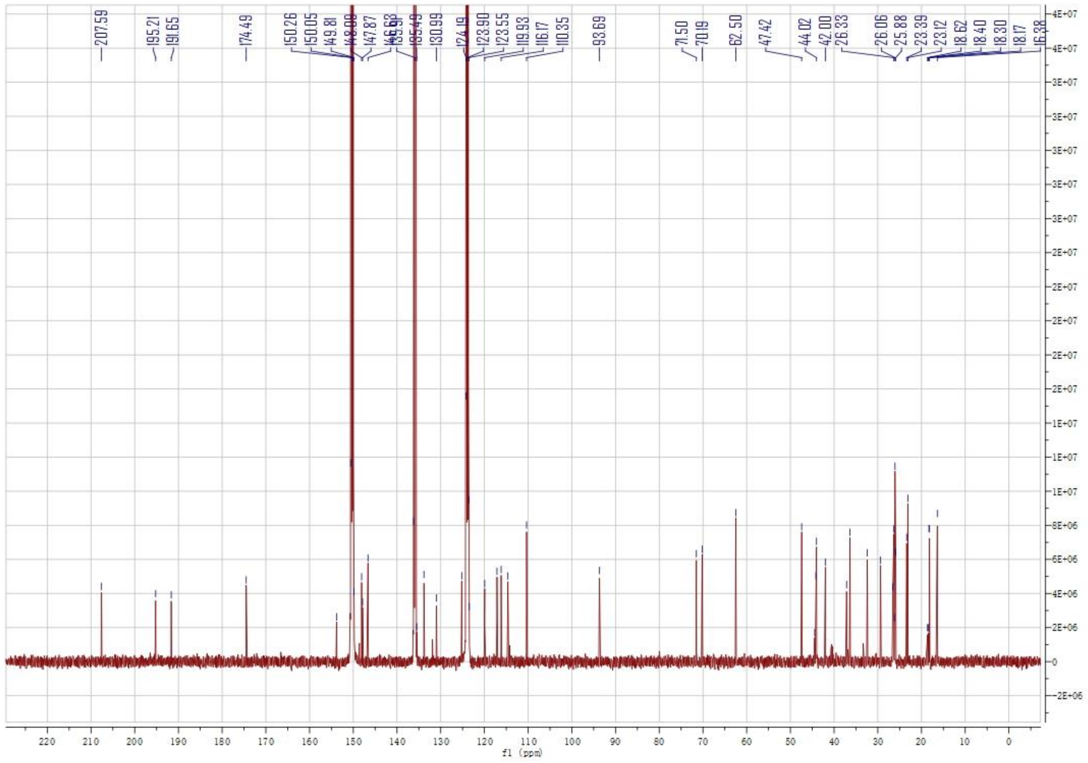

Figure S11. ^{13}C NMR spectrum of compound **5**

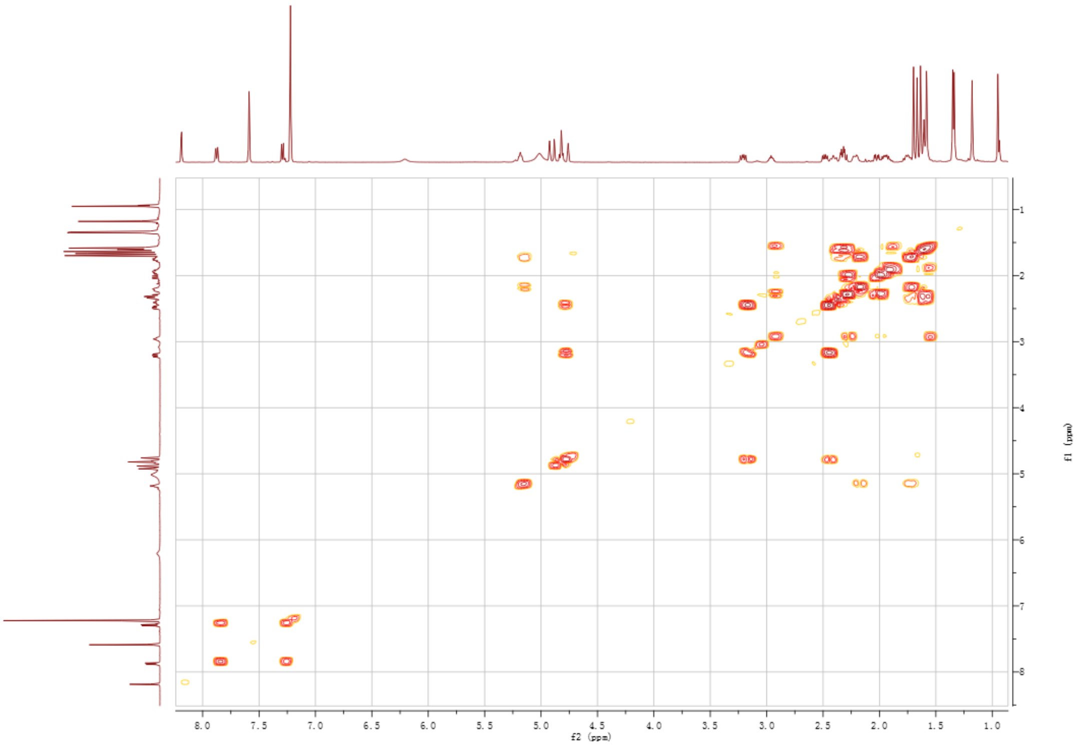

Figure S12. ^{1}H ^{1}HCOSY spectrum of compound **5**

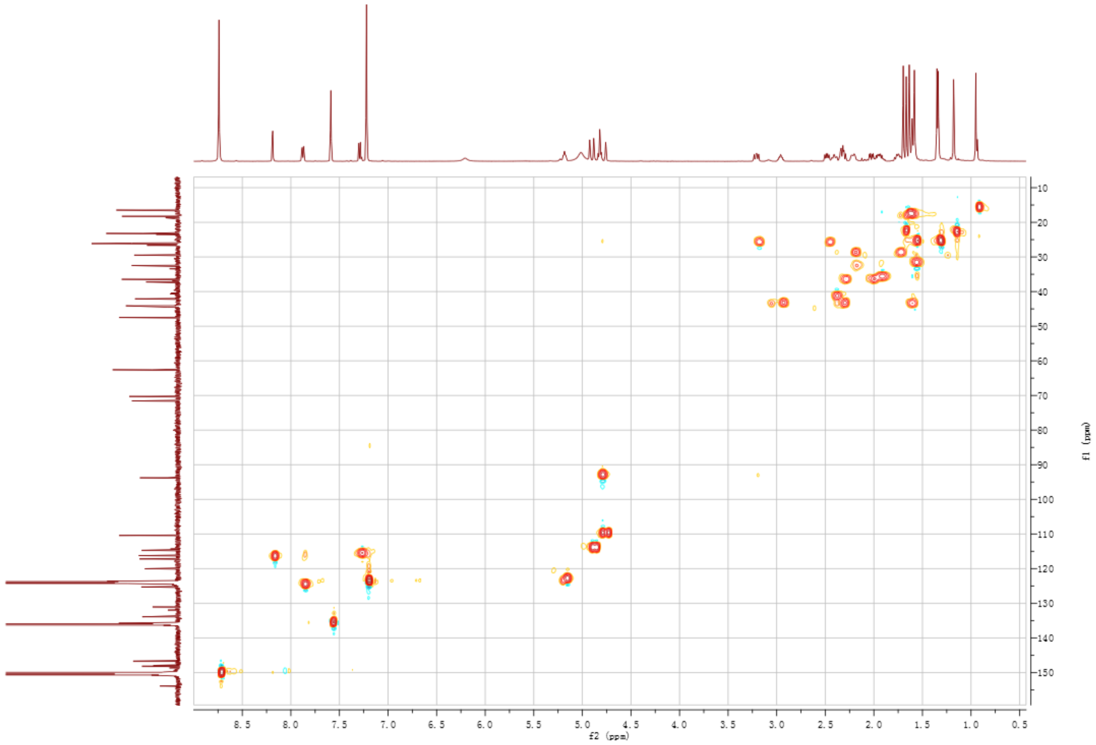

Figure S13. HSQC spectrum of compound **5**

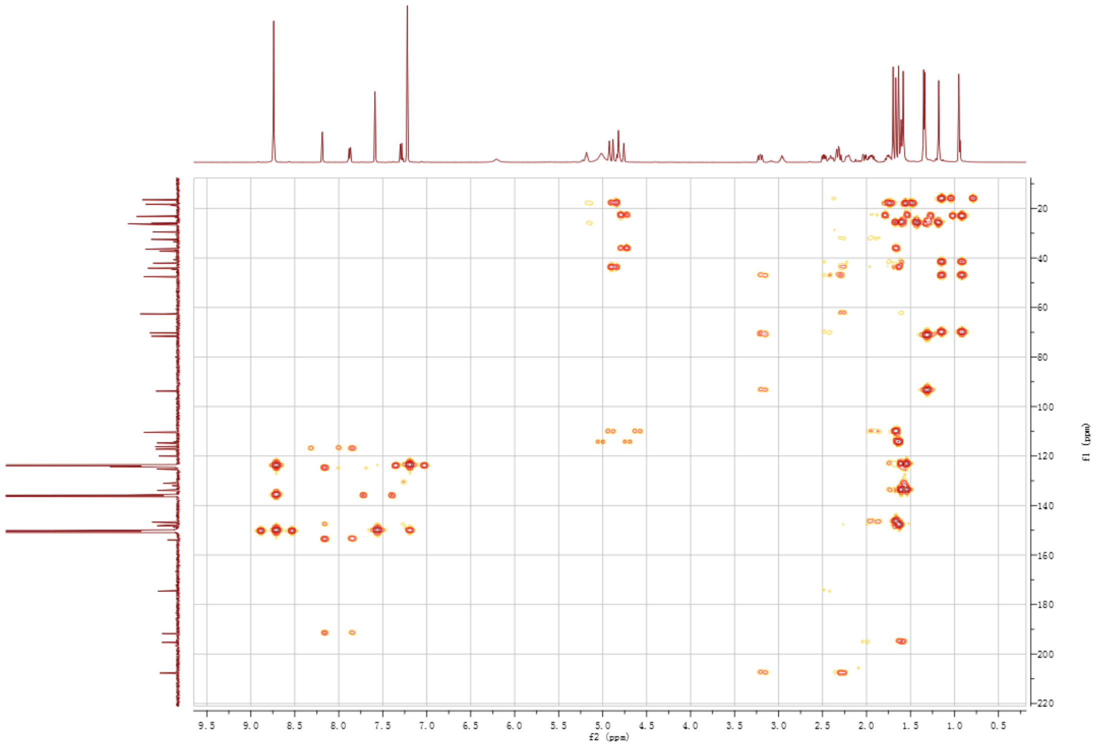

Figure S14. HMBC spectrum of compound **5**

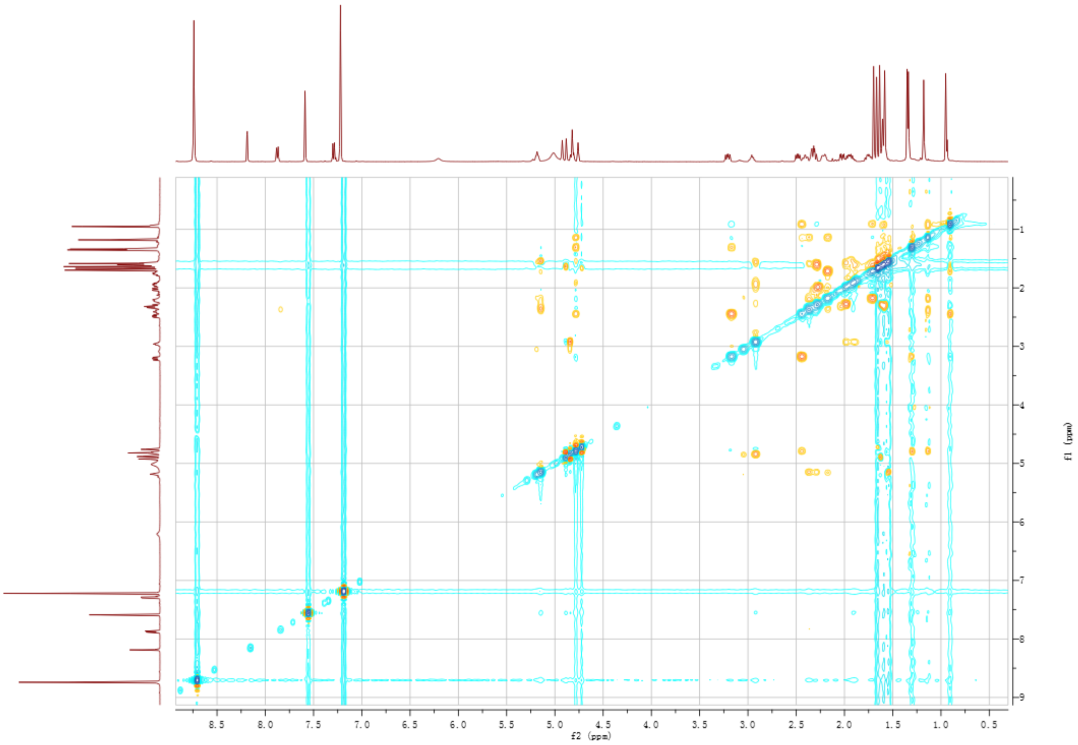

Figure S15. ROESY spectrum of compound **5**

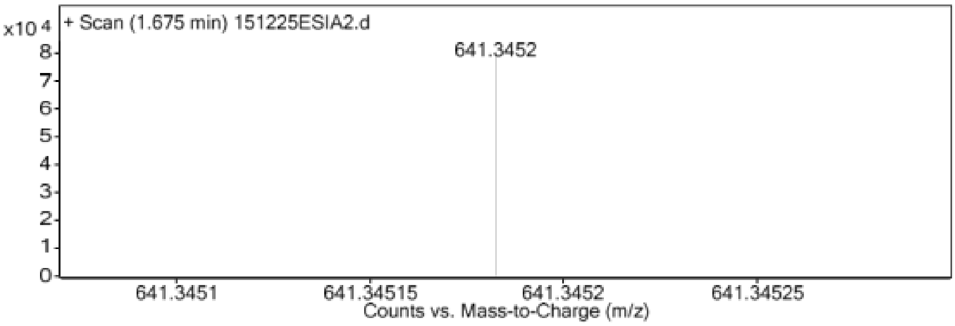

Peak List

m/z	z	Abund	Formula	Ion
121.0509		20996.01		
232.1118	1	19346.32		
619.3627	1	32013.49		
620.3689	1	12487.3		
641.3452	1	80172.41	C38 H50 Na O7	M+
642.3485	1	28720.66	C38 H50 Na O7	M+
922.0098	1	21191.2		
1259.7005	1	69492.96		
1260.7038	1	55731.16		
1261.7065	1	20609.47		

Formula Calculator Element Limits

Element	Min	Max
C	0	200
H	0	400
O	0	9
Na	1	1

Formula Calculator Results

Formula	CalculatedMass	Mz	Diff.(mDa)	Diff. (ppm)	DBE
C38 H50 Na O7	641.3454	641.3452	0.2	0.3	13.5

Figure S16. HR-ESI-MS spectrum of compound **5**

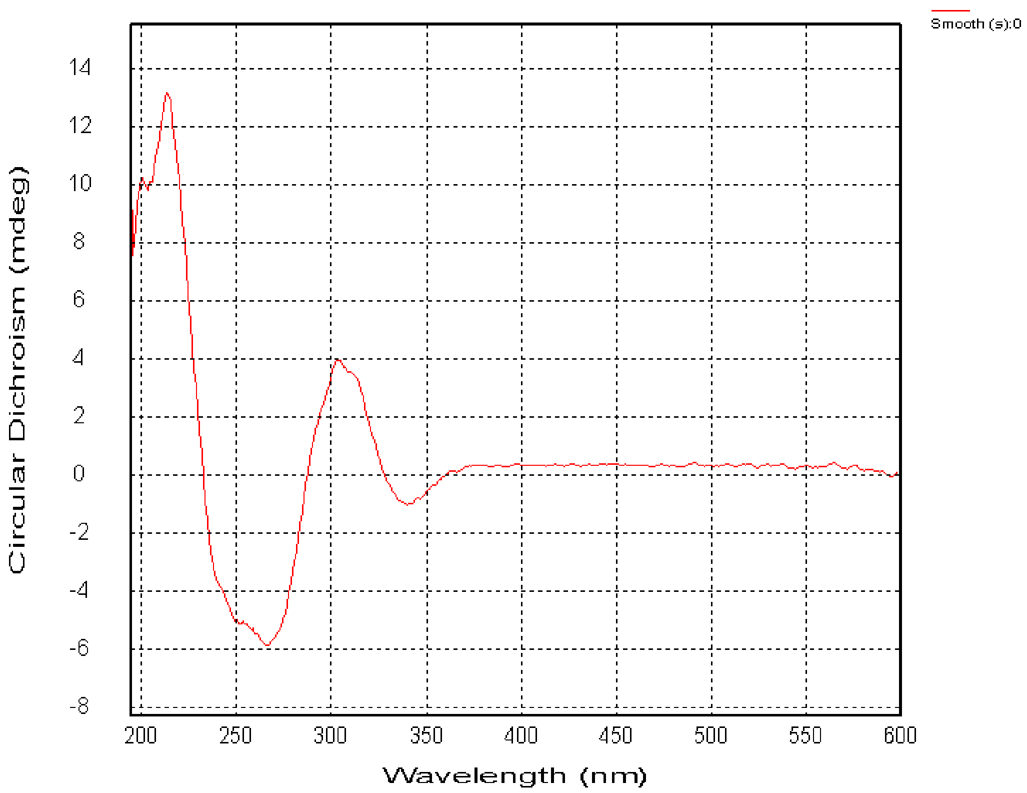

Figure S17. CD spectrum of compound **5**

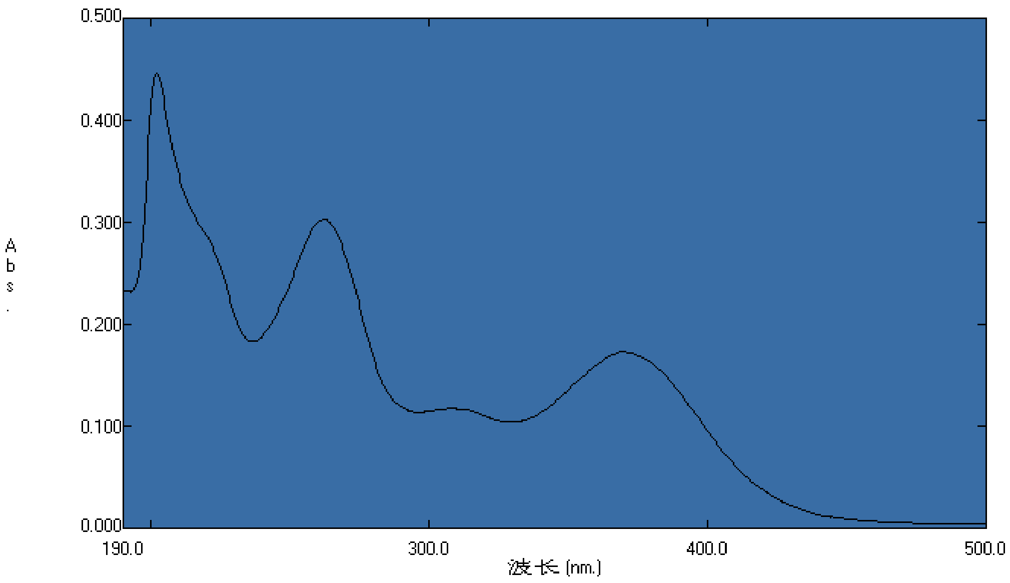

Figure S18. UV spectrum of compound **5**

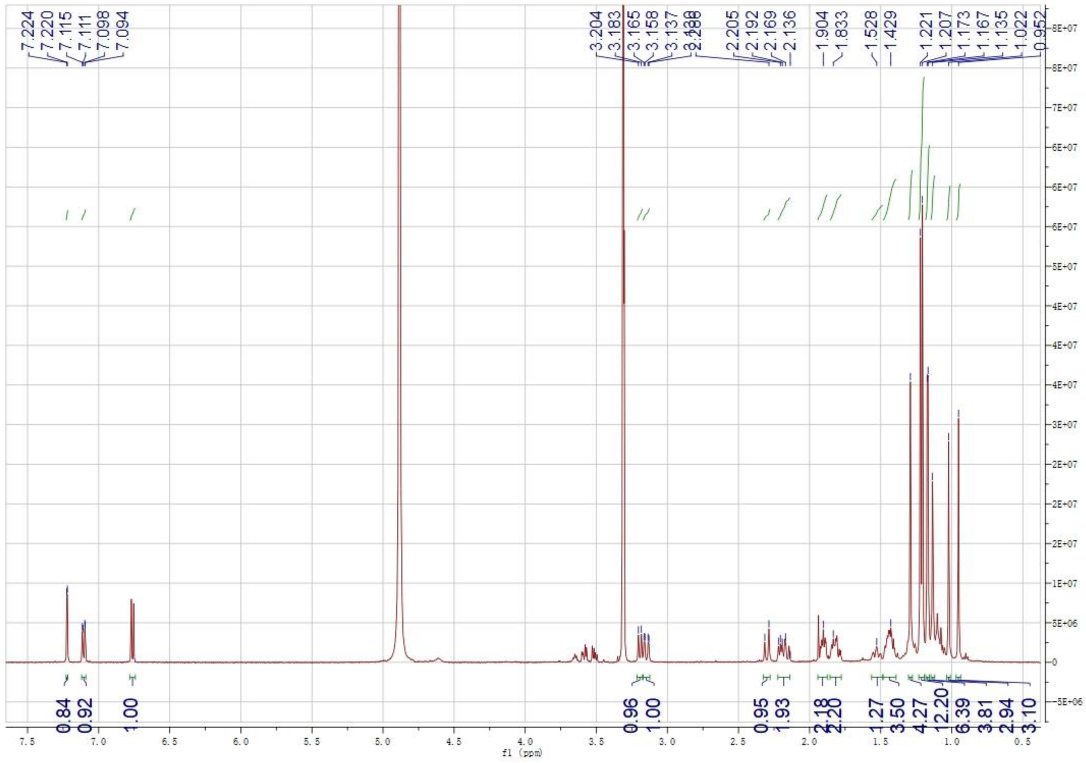

Figure S19. ^{1}H NMR spectrum of compound **7**

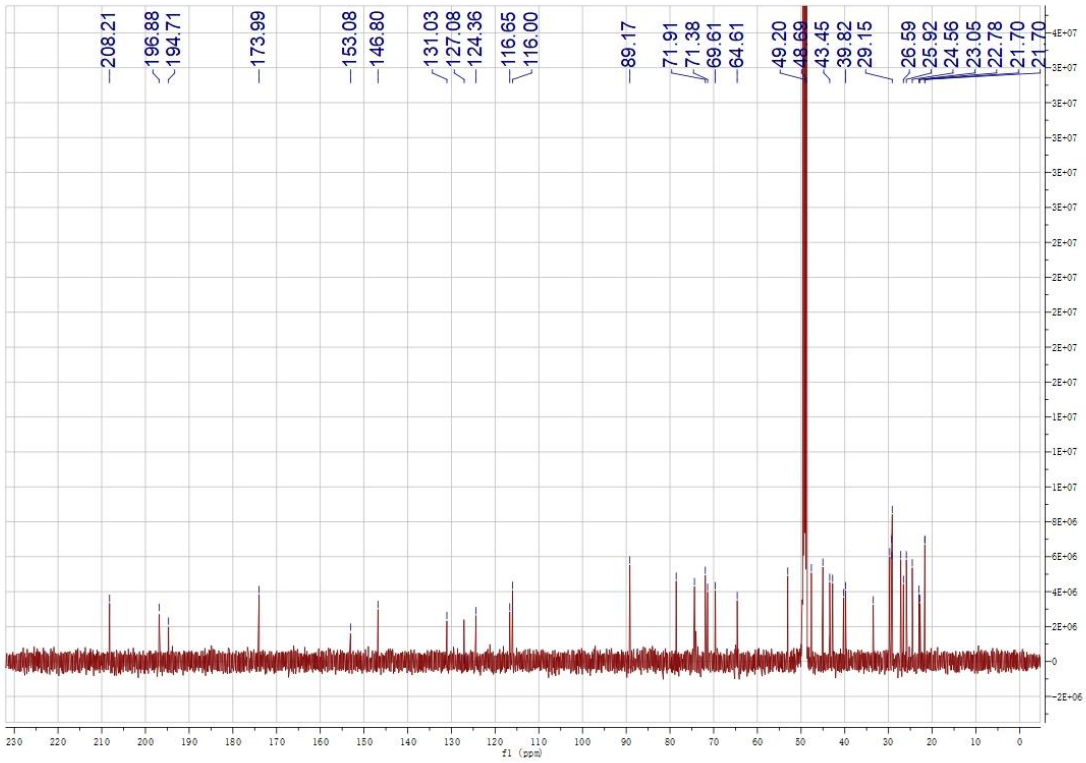

Figure S20. ^{13}C NMR spectrum of compound **7**

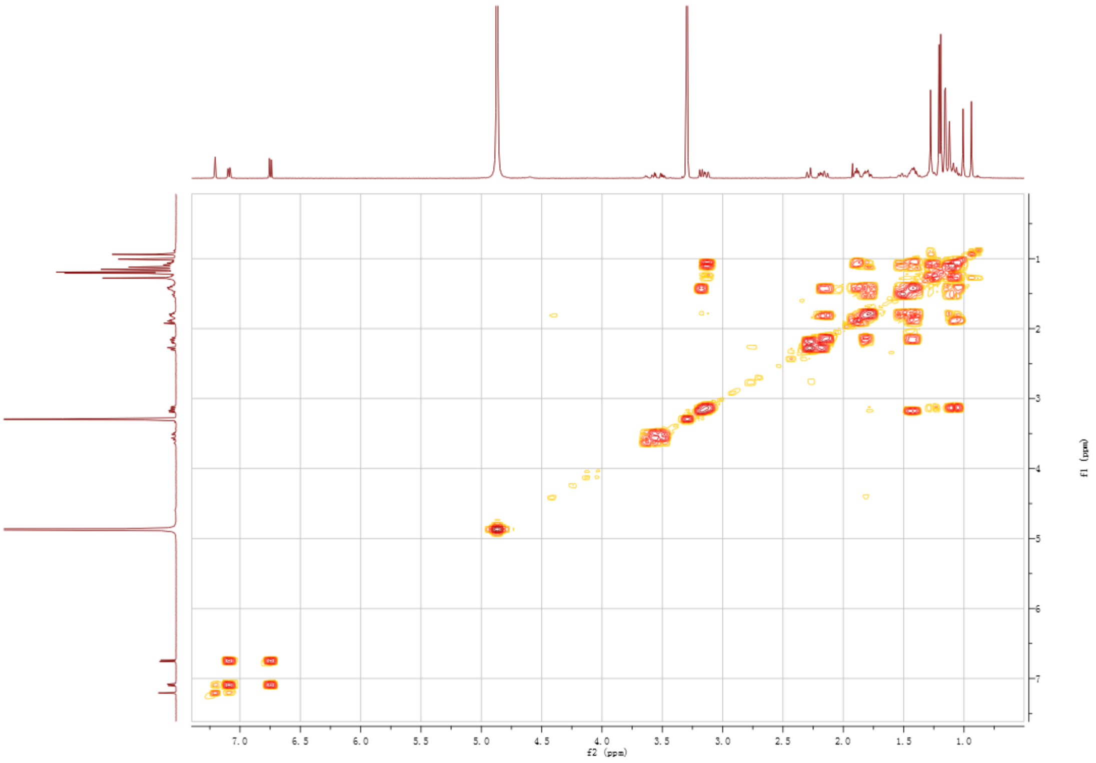

Figure S21. ^{1}H-^{1}HCOSY spectrum of compound **7**

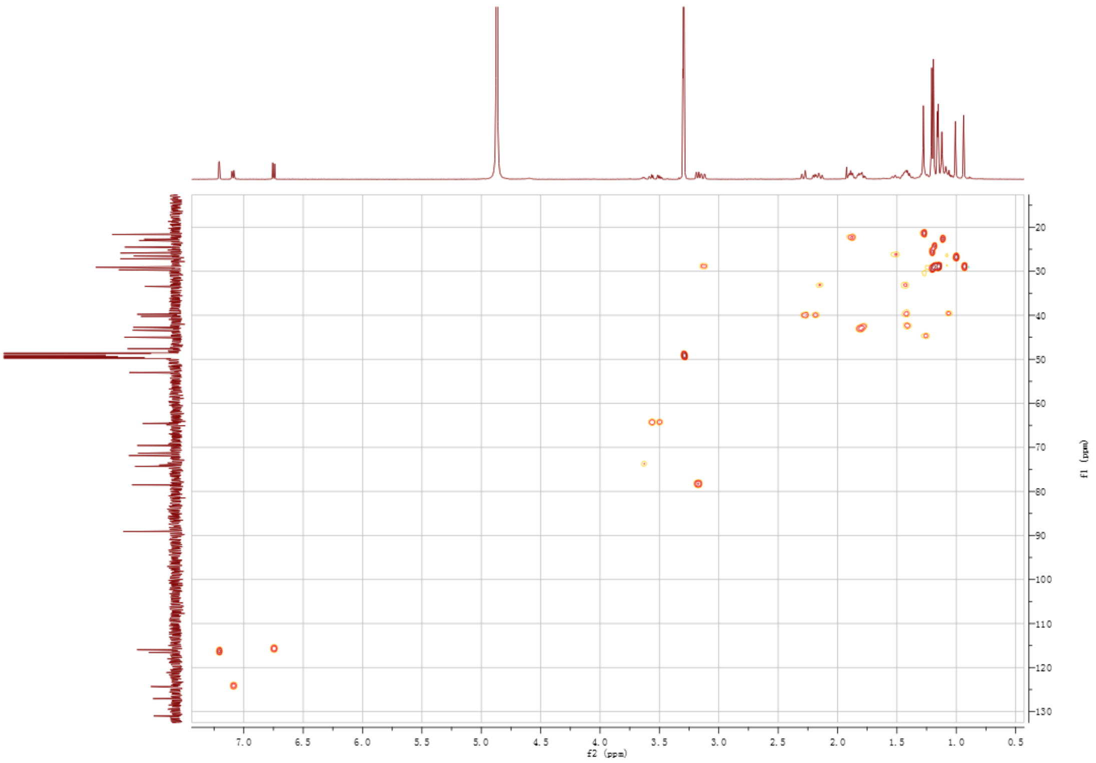

Figure S22. HSQC spectrum of compound **7**

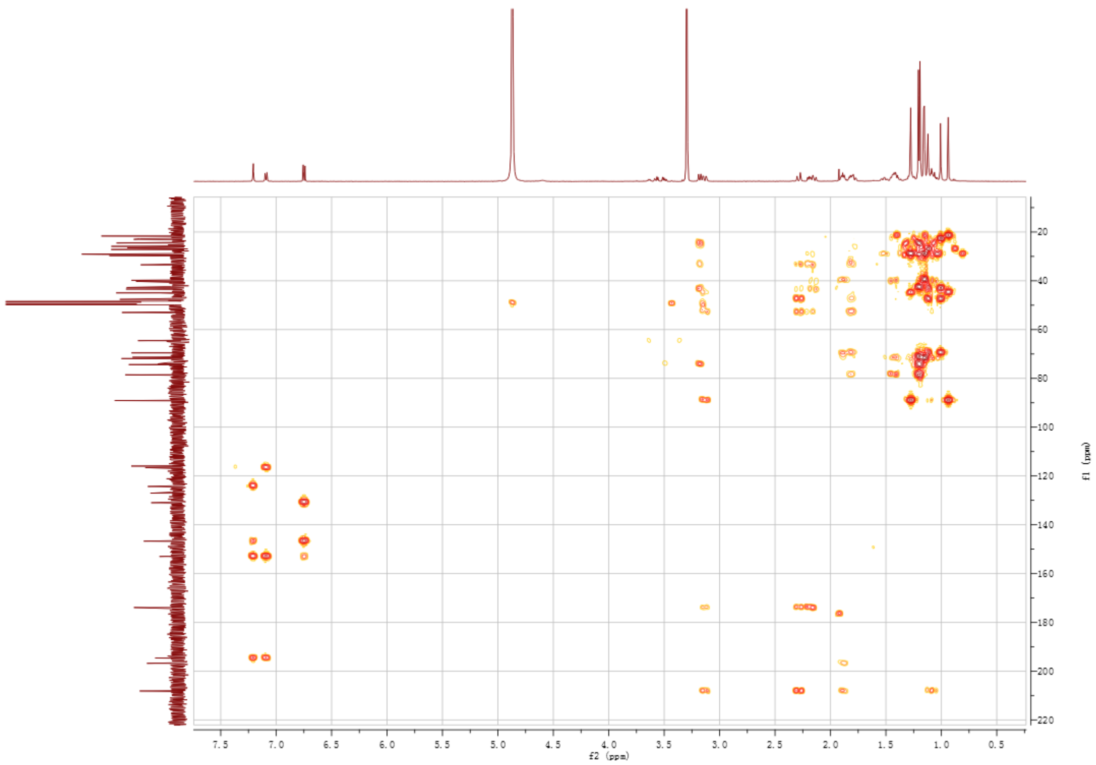

Figure S23. HMBC spectrum of compound **7**

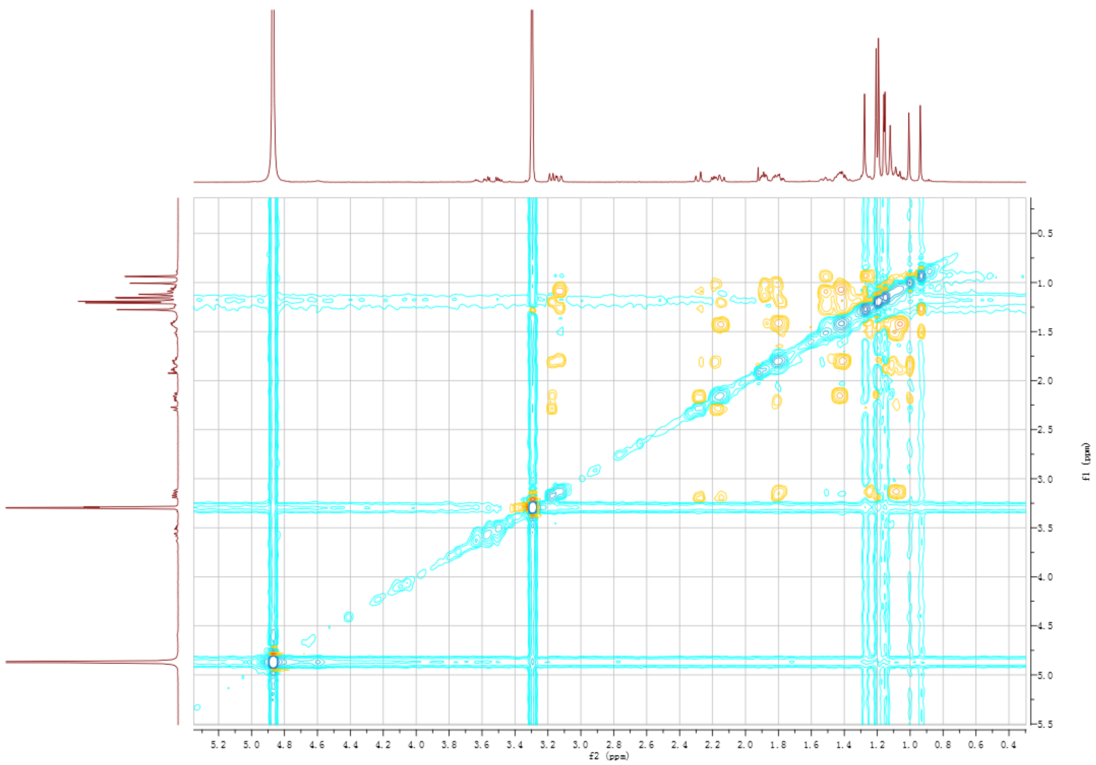

Figure S24. ROESY spectrum of compound **7**

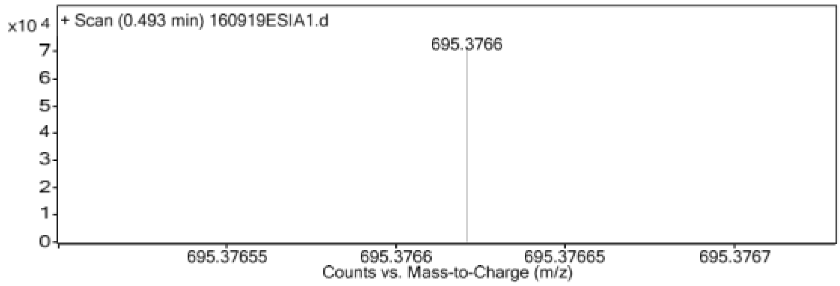

Peak List

m/z	z	Abund	Formula	Ion
121.0509	1	51364.41		
274.2736	1	18445.03		
425.2146	1	33774.45		
673.3942	1	13041.42		
695.3766	1	71912.7	C38 H56 Na O10	M+
696.3796	1	23815.15	C38 H56 Na O10	M+
711.3519	1	12881.7		
922.0098	1	59510		
1367.7612	1	22479.28		
1368.7651	1	17675.48		

Formula Calculator Element Limits

Element	Min	Max
C	0	200
H	0	400
O	0	12
Na	1	1

Formula Calculator Results

Formula	CalculatedMass	Mz	Diff.(mDa)	Diff. (ppm)	DBE
C38 H56 Na O10	695.3771	695.3766	0.5	0.7	10.5

Figure S25. HR-ESI-MS spectrum of compound **7**

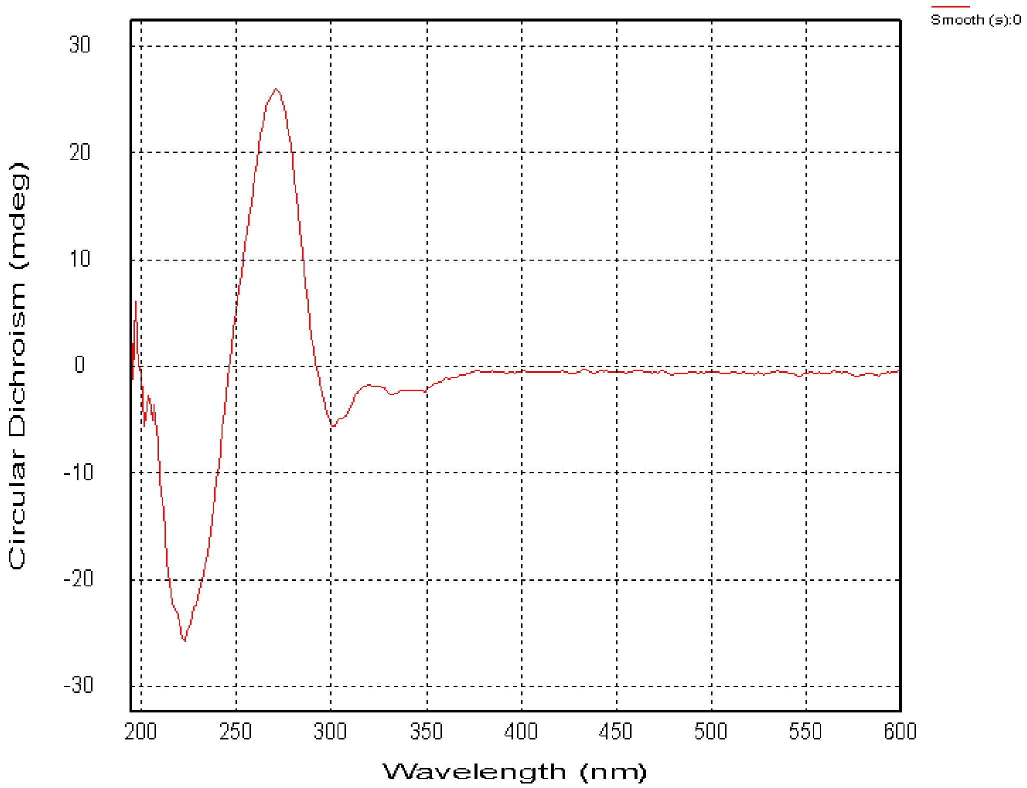

Figure S26. CD spectrum of compound **7**

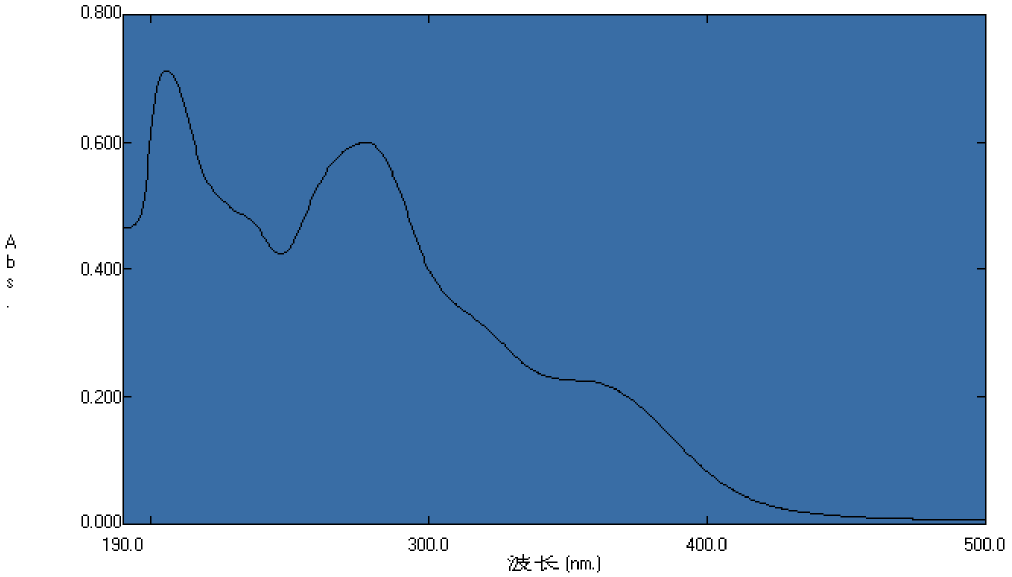

Figure S27. UV spectrum of compound **7**

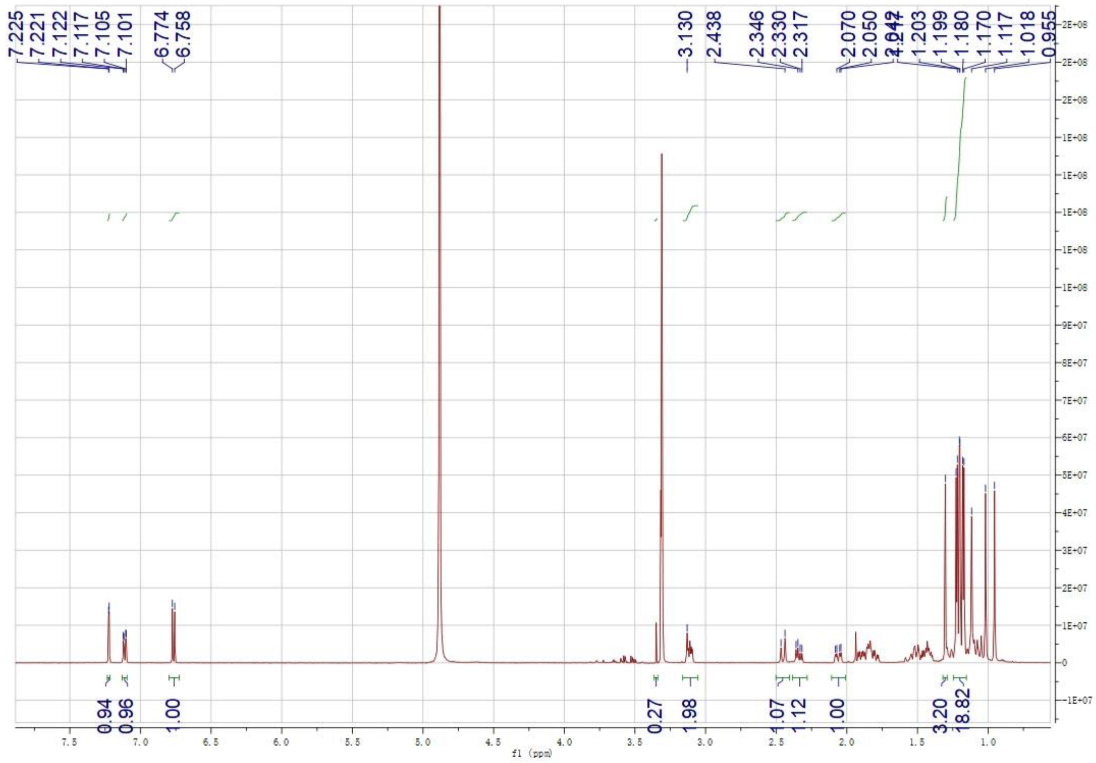

Figure S28. ^{1}H NMR spectrum of compound **8**

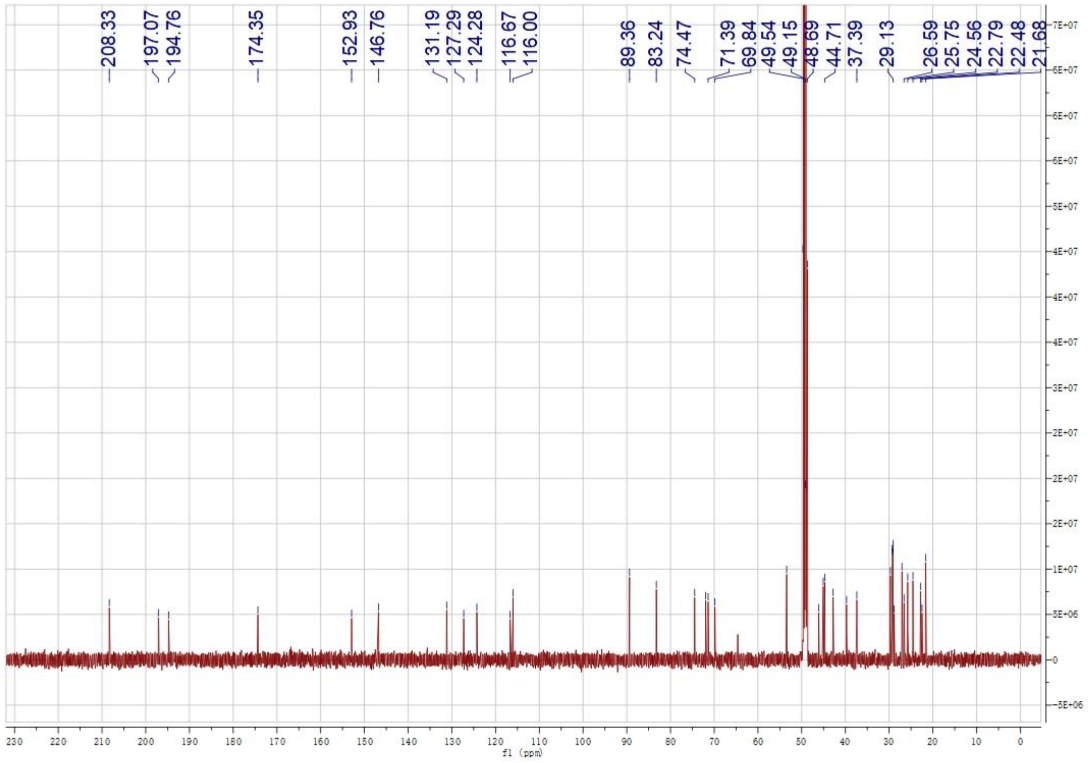

Figure S29. ^{13}C NMR spectrum of compound **8**

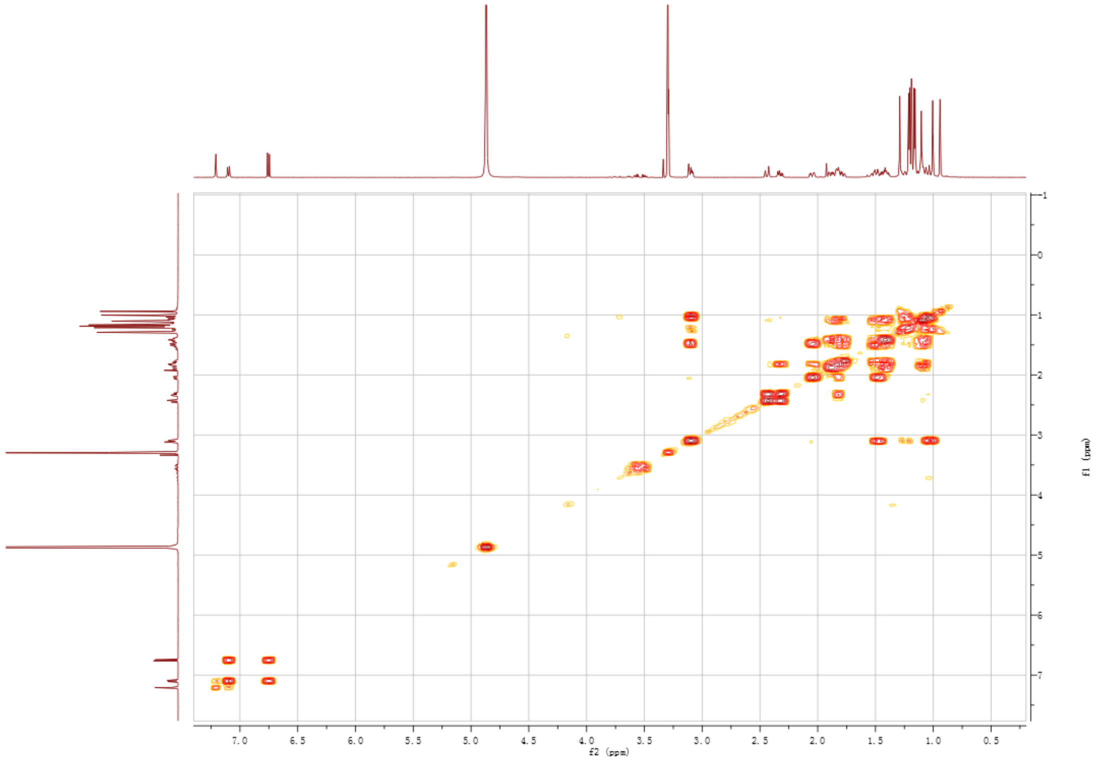

Figure S30.^{1}H -^{1}HCOSY spectrum of compound **8**

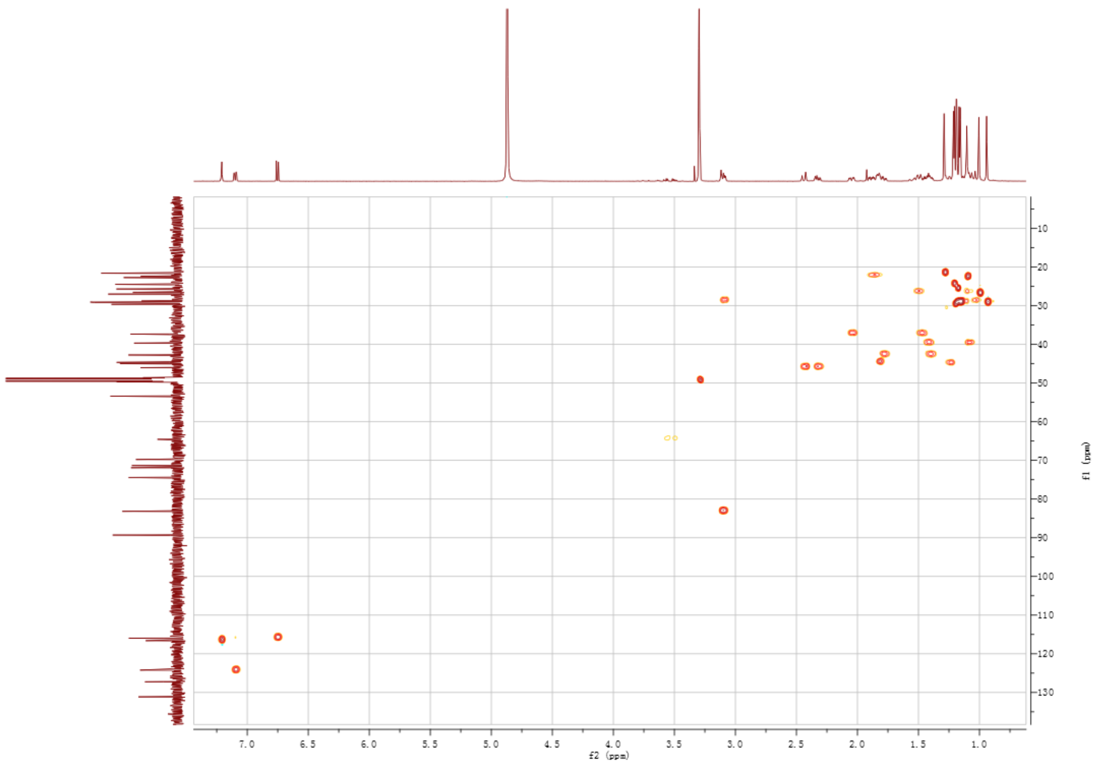

Figure S31. HSQC spectrum of compound **8**

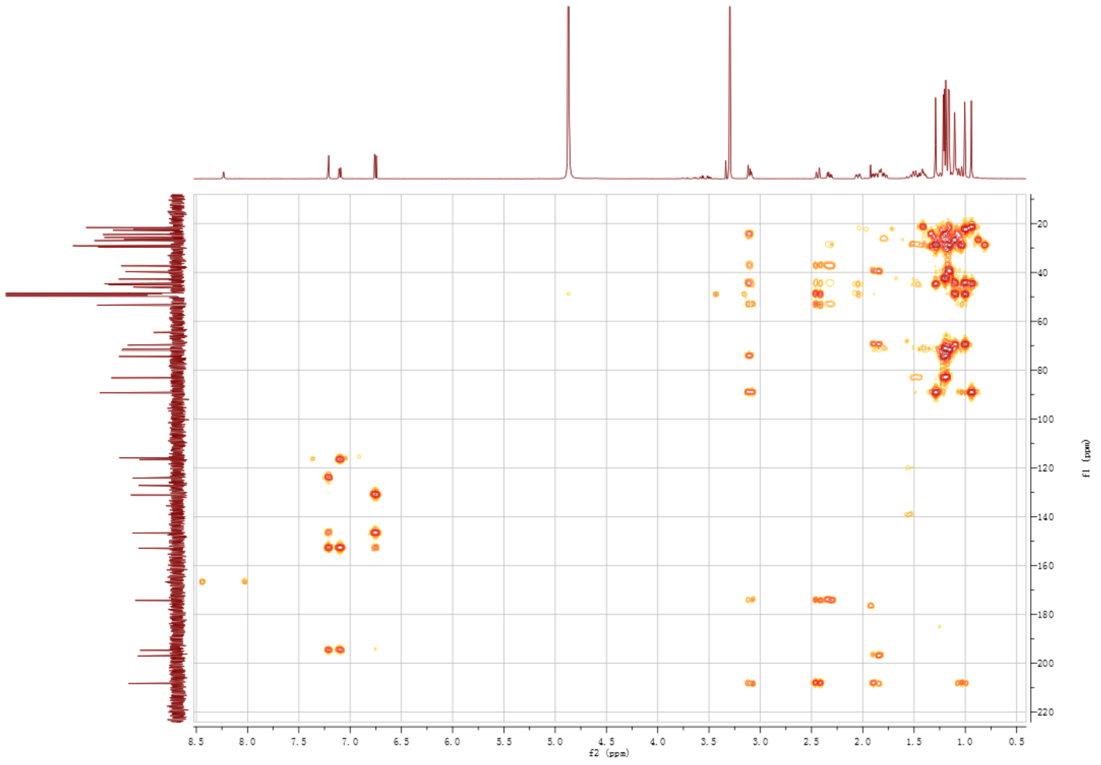

Figure S32. HMBC spectrum of compound **8**

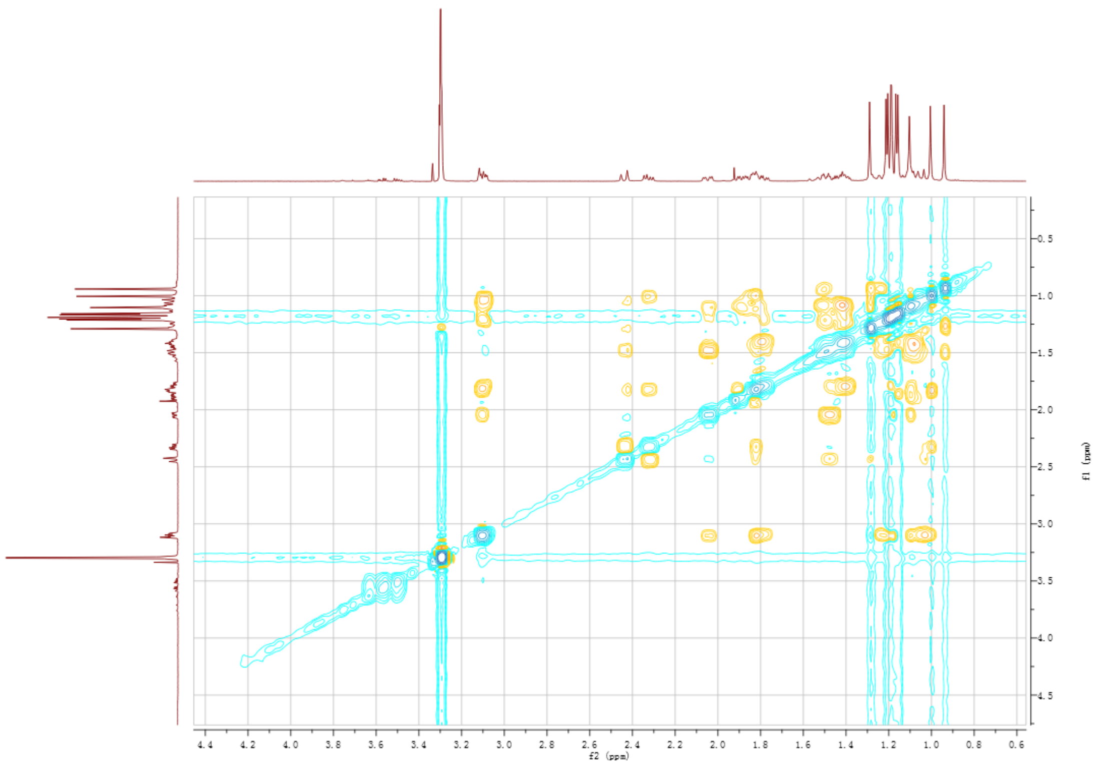

Figure S33. ROESY spectrum of compound **8**

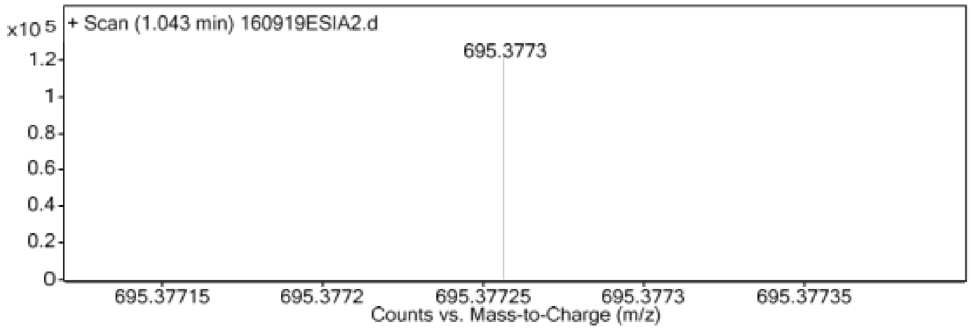

Peak List

m/z	z	Abund	Formula	Ion
121.0509	1	34114.15		
673.395	1	75069.27		
674.398	1	24636.68		
695.3773	1	123813.91	C38 H56 Na O10	M+
696.3801	1	43001.16	C38 H56 Na O10	M+
922.0098	1	38828.61		
1362.8071	1	29009.37		
1367.7638	1	172719.64		
1368.7673	1	141331.05		
1369.7698	1	56734.86		

Formula Calculator Element Limits

Element	Min	Max
C	0	200
H	0	400
O	0	12
Na	1	1

Formula Calculator Results

Formula	CalculatedMass	Mz	Diff.(mDa)	Diff. (ppm)	DBE
C38 H56 Na O10	695.3771	695.3773	-0.2	0.3	10.5

Figure S34. HR-ESI-MS spectrum of compound **8**

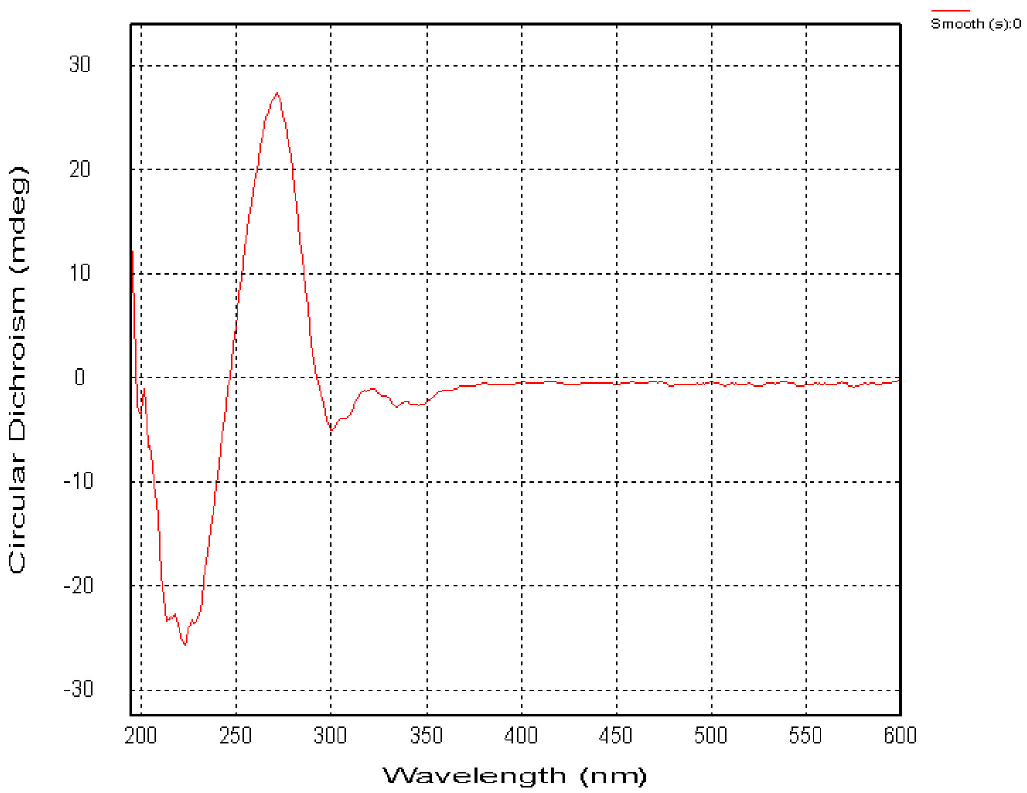

Figure S35. CD spectrum of compound **8**

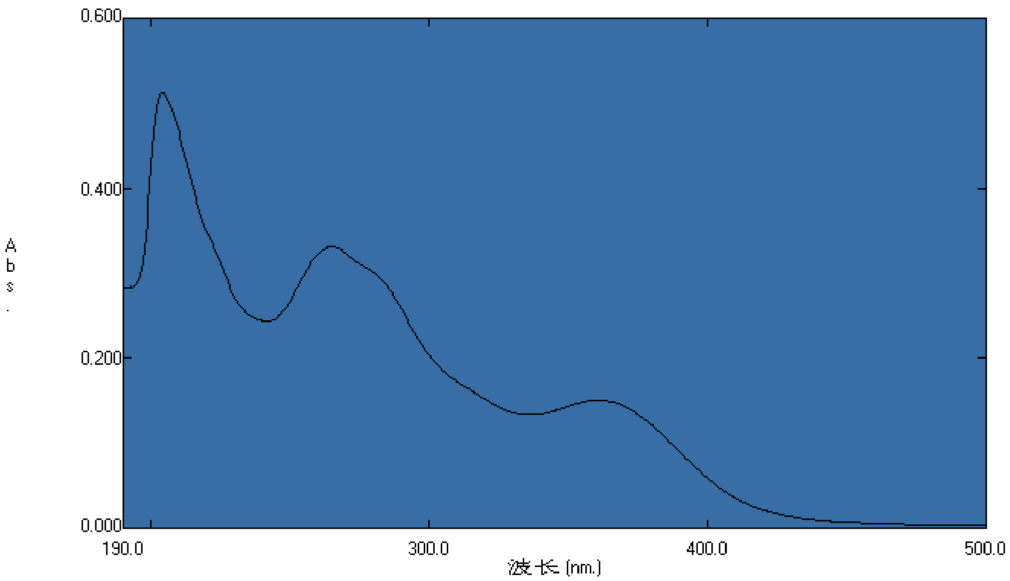

Figure S36. UV spectrum of compound **8**

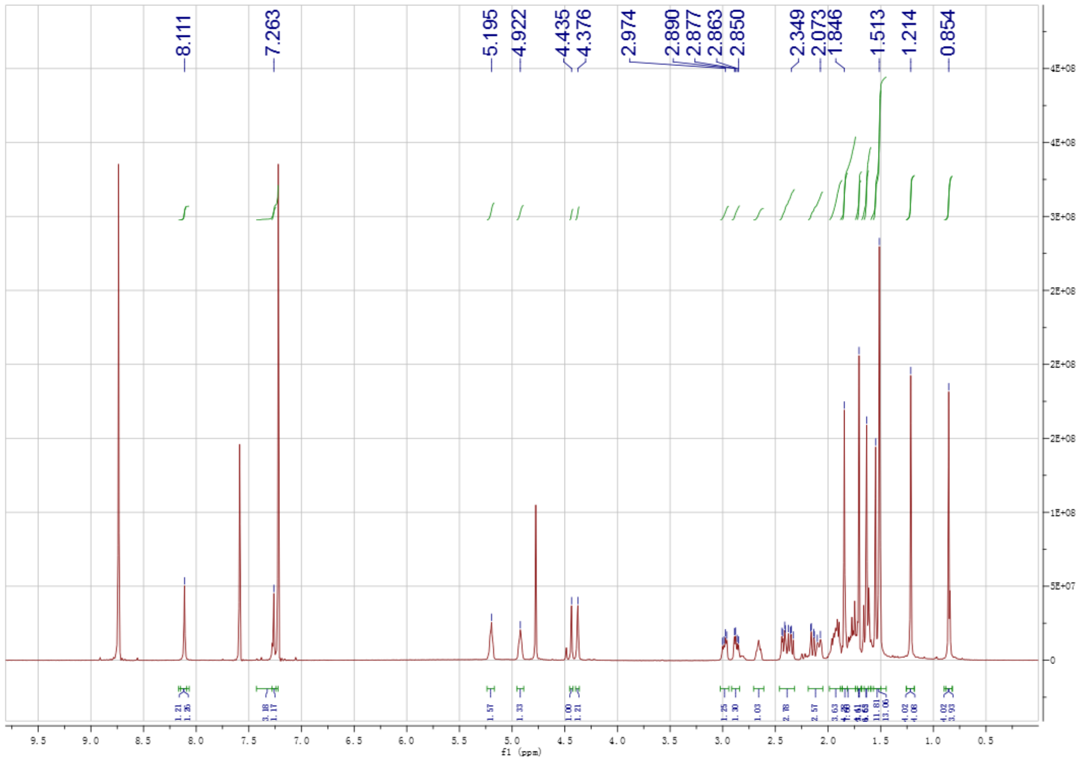

Figure S37. ^{1}H NMR spectrum of compound **11**

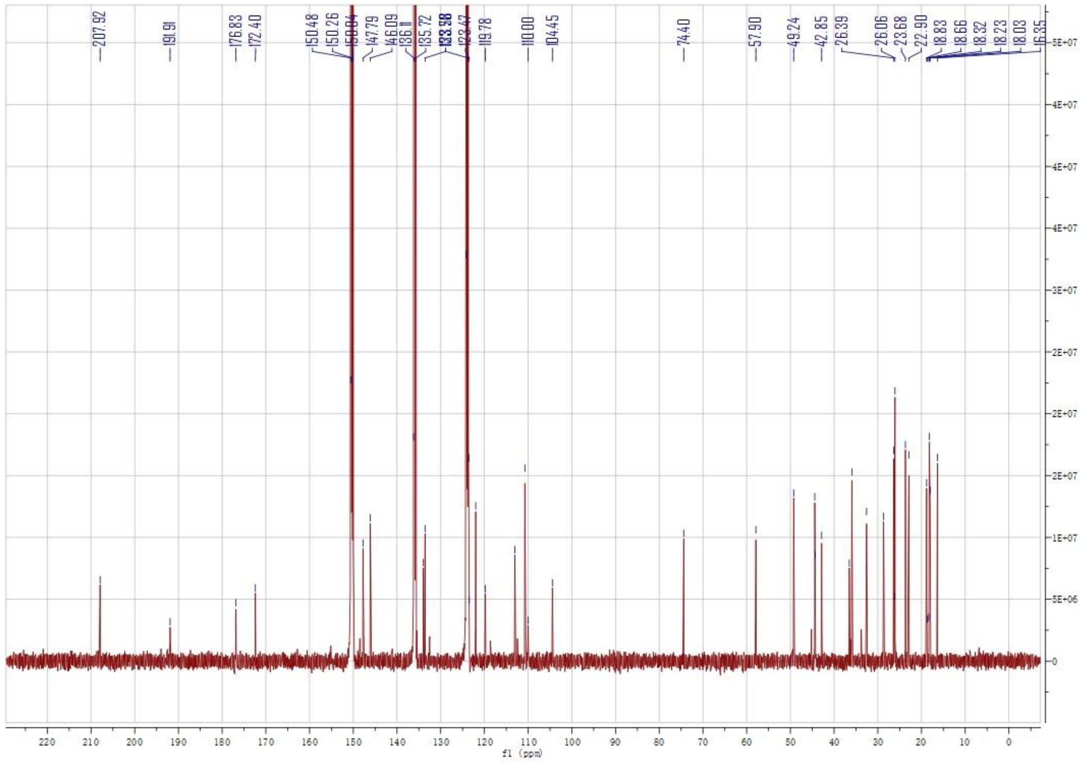

Figure S38. ^{13}C NMR spectrum of compound **11**

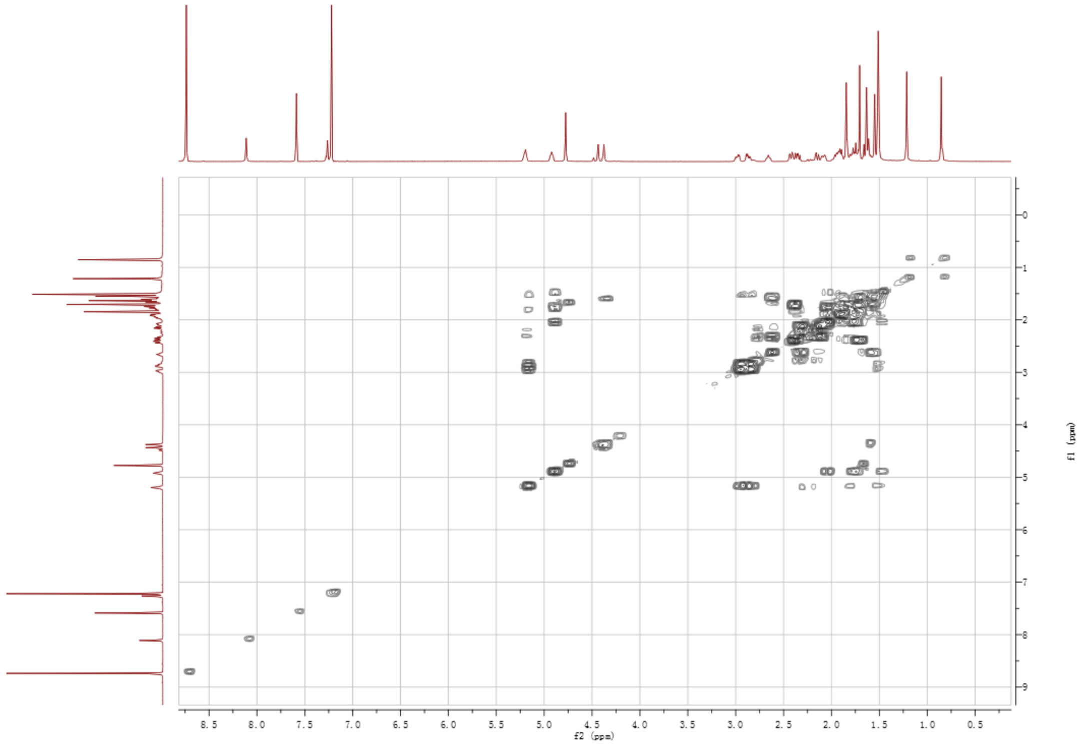

Figure S39. ^{1}H -^{1}H COSY spectrum of compound **11**

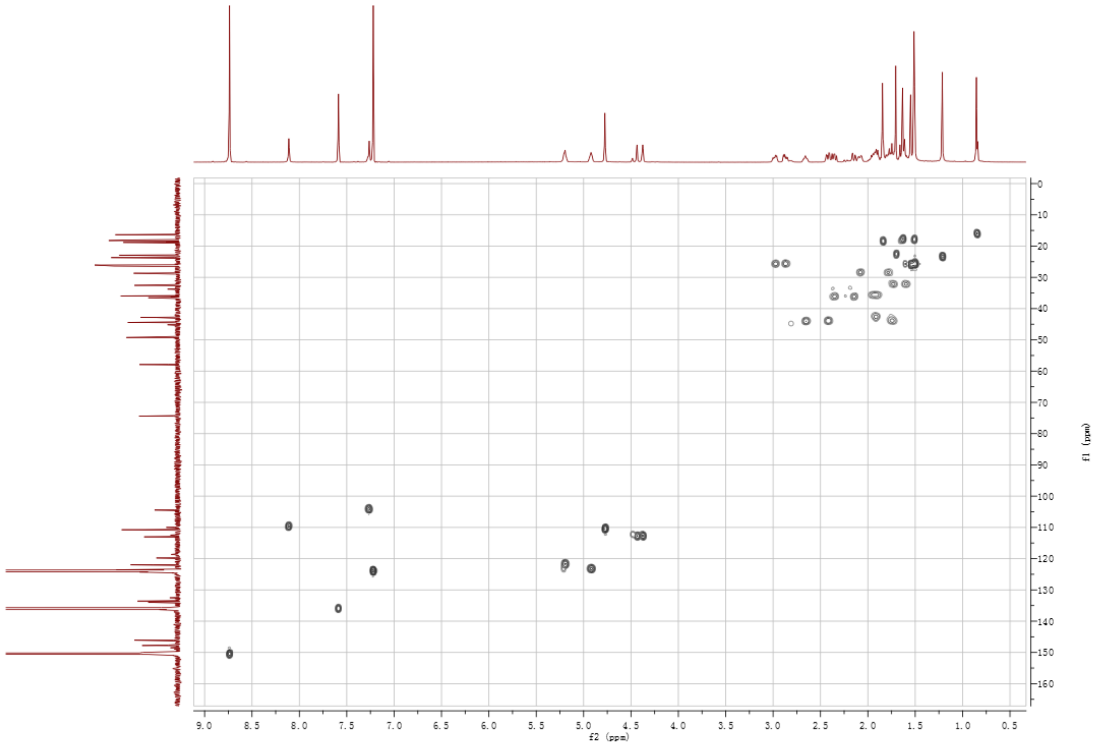

Figure S40. HSQC spectrum of compound **11**

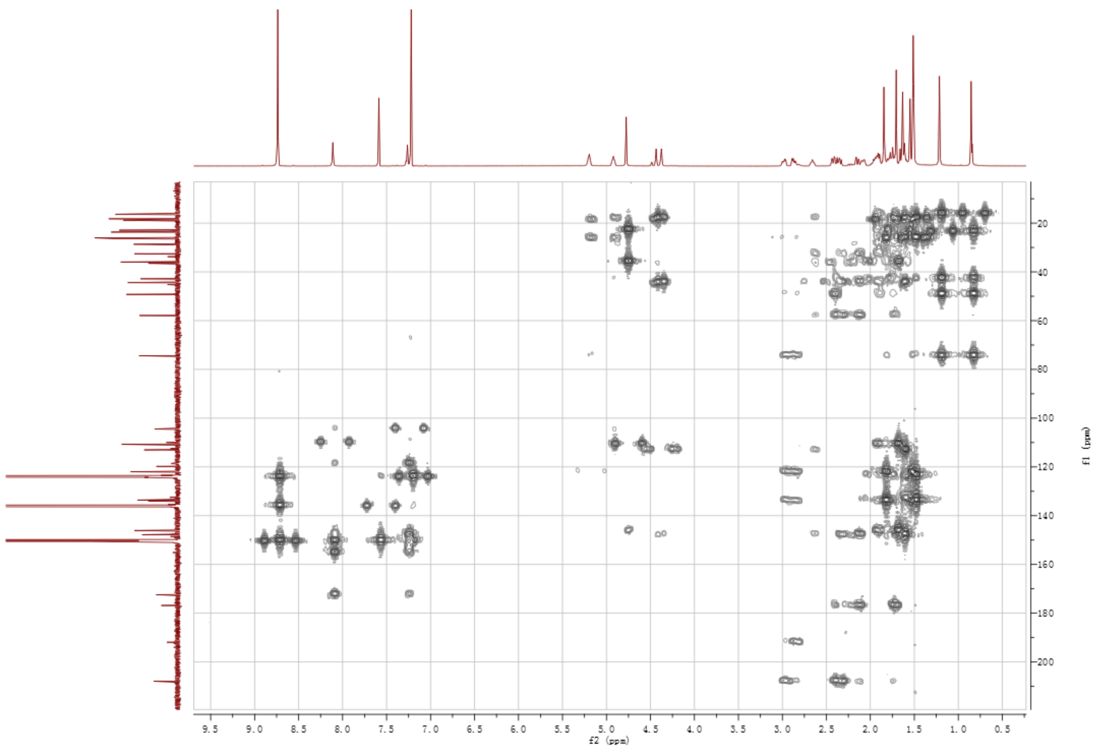

Figure S41. HMBC spectrum of compound **11**

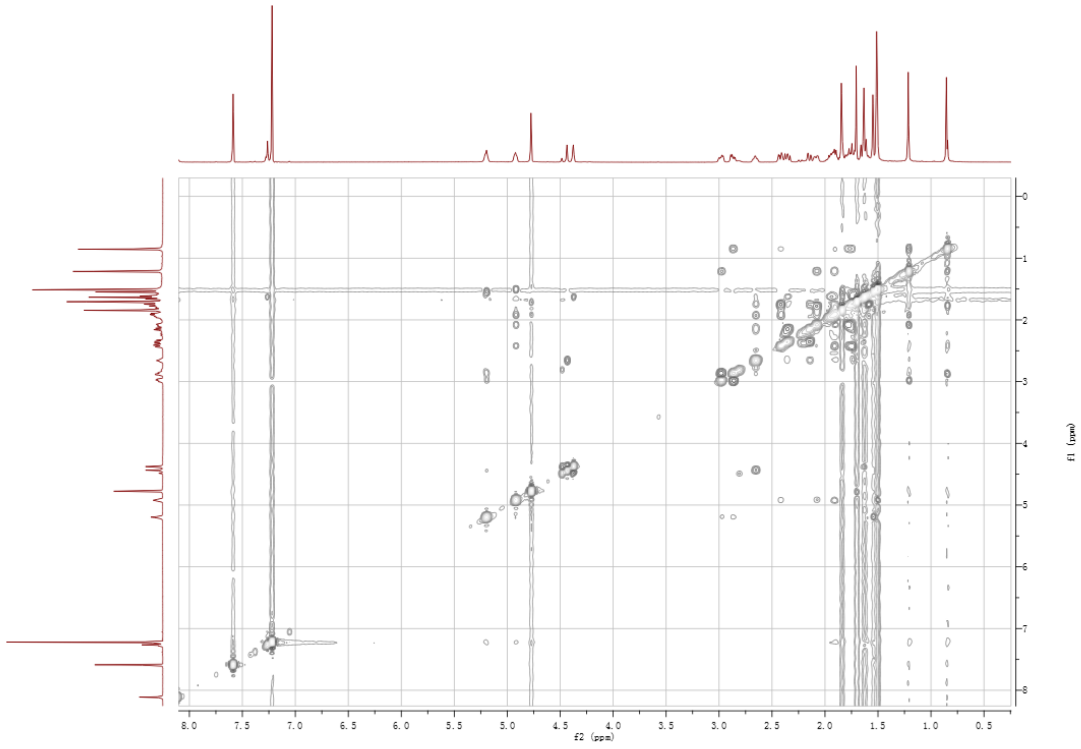

Figure S42. ROESY spectrum of compound **11**

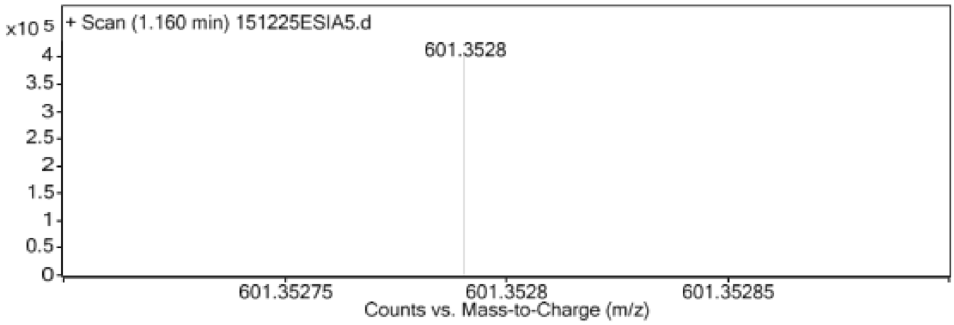

Peak List

m/z	z	Abund	Formula	Ion
232.1123	1	133430.34		
601.3528	1	409202.16	C38 H49 O6	M+
602.3559	1	158666.92	C38 H49 O6	M+
623.3344	1	146258.17		
922.0098	1	90576.55		
1201.6975	1	144697.56		
1202.7012	1	122529.56		
1223.6796	1	200306.56		
1224.683	1	165874.39		
1825.0277	1	87352.64		

Formula Calculator Element Limits

Element	Min	Max
C	0	200
H	0	400
O	0	9

Formula Calculator Results

Formula	CalculatedMass	Mz	Diff.(mDa)	Diff. (ppm)	DBE
C38 H49 O6	601.3529	601.3528	0.1	0.2	14.5

Figure S43. HR-ESI-MS spectrum of compound **11**

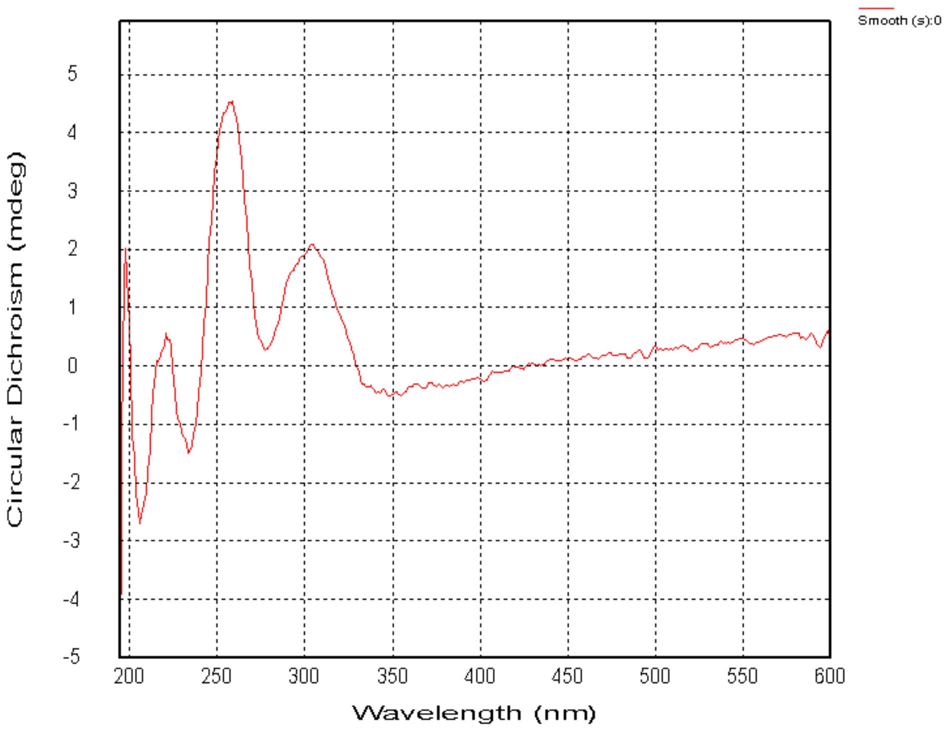

Figure S44. CD spectrum of compound **11**

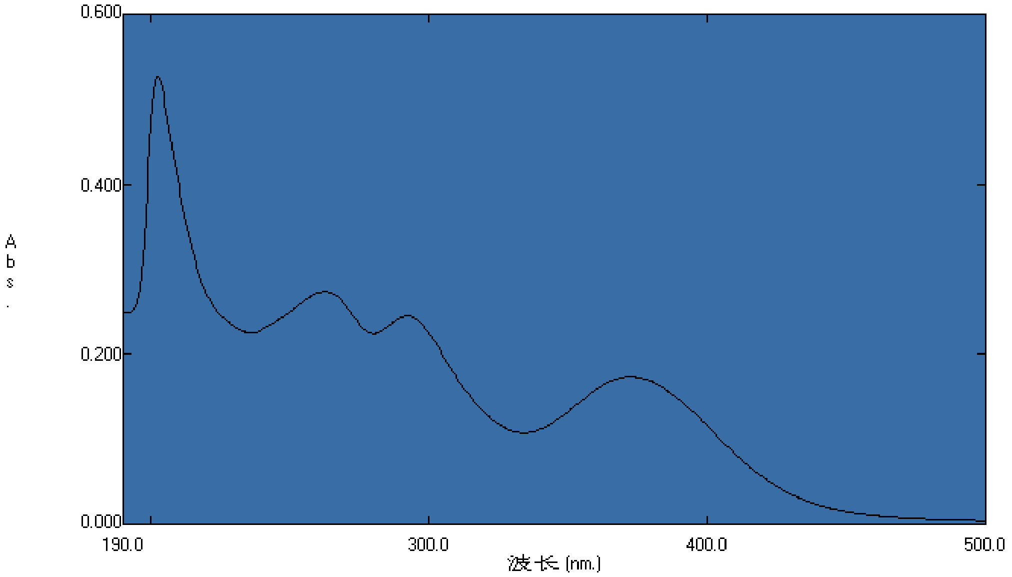

Figure S45. UV spectrum of compound **11**

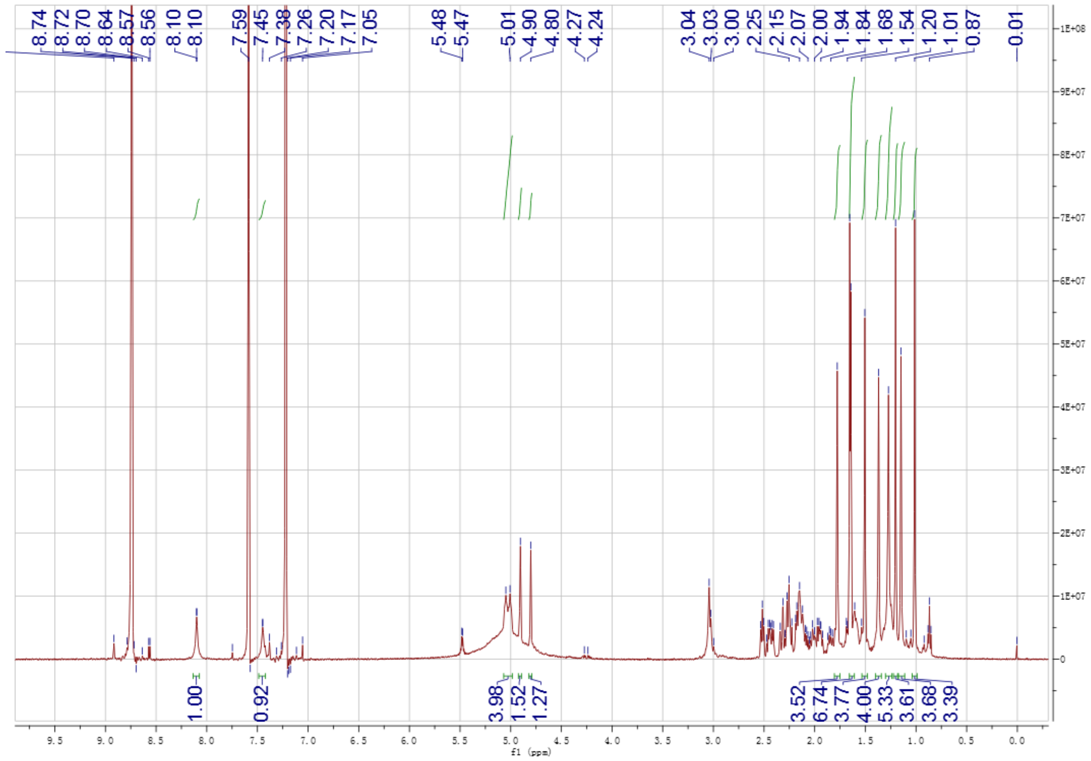

Figure S46. ^{1}H NMR spectrum of compound **15**

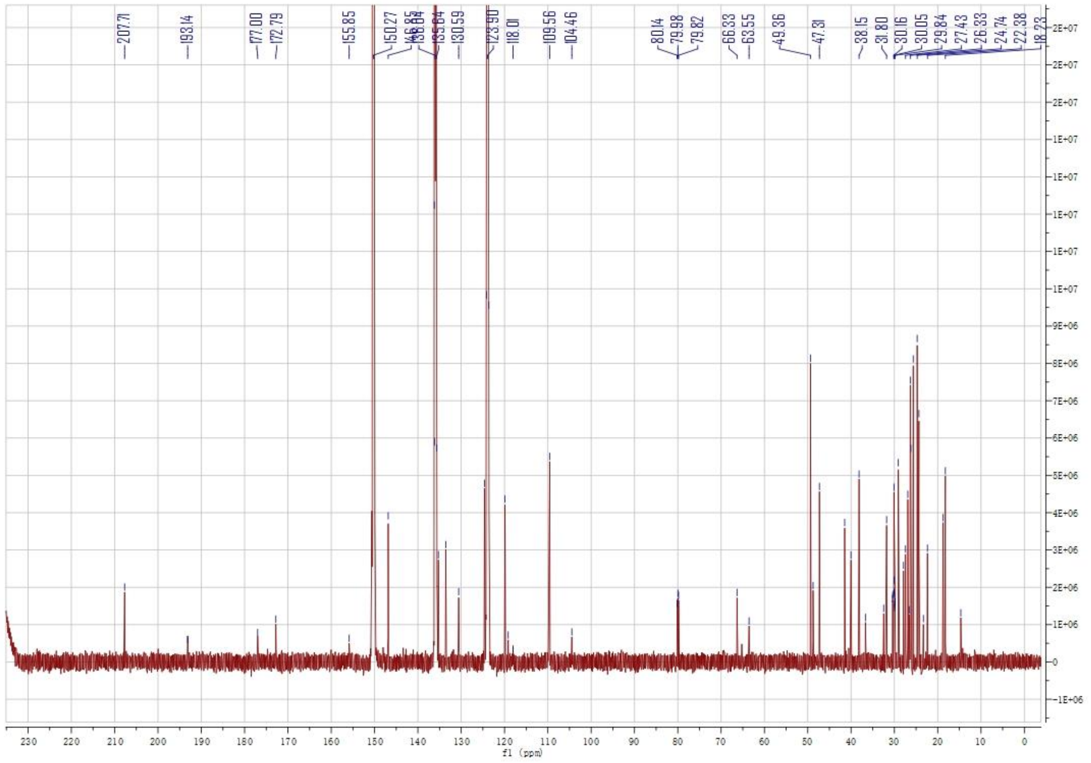

Figure S47. ^{13}C NMR spectrum of compound **15**

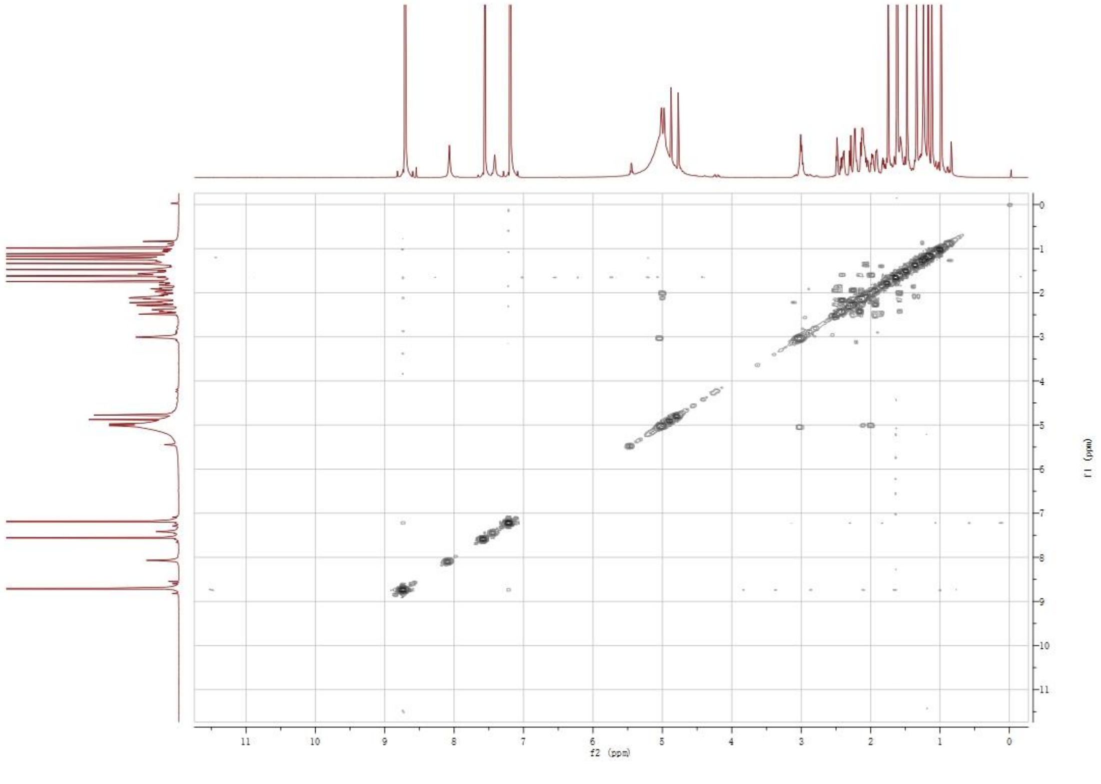

Figure S48.^{1}H -^{1}HCOSY spectrum of compound **15**

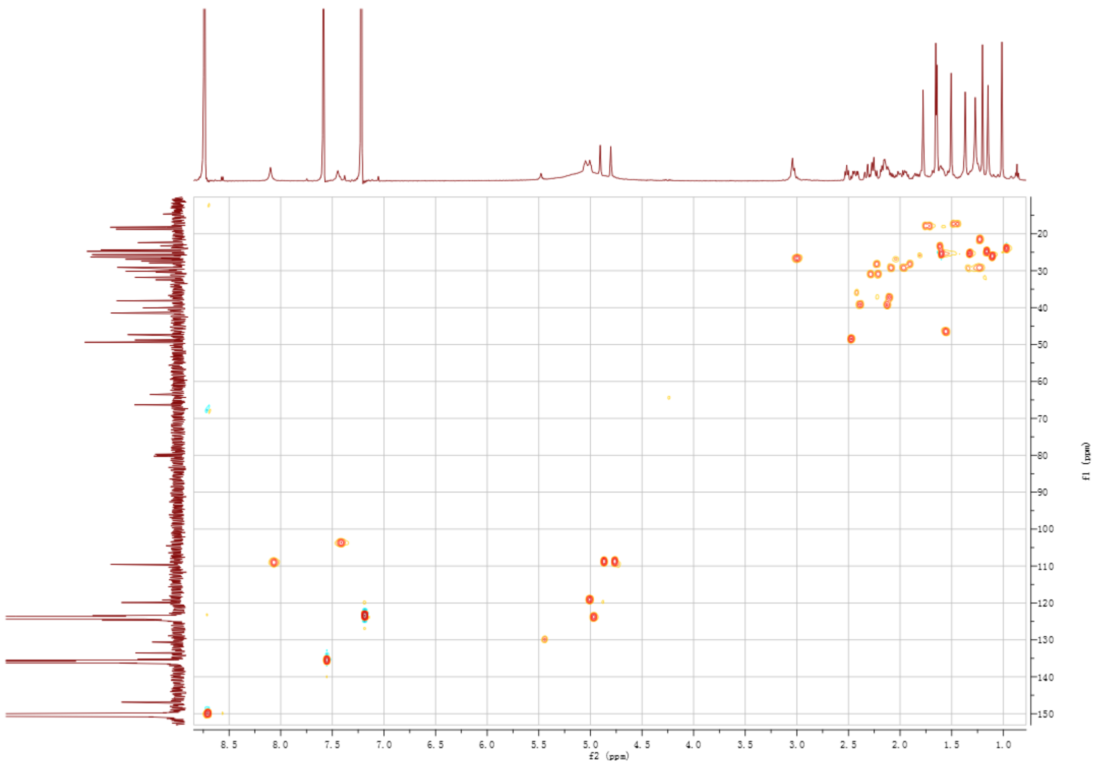

Figure S49. HSQC spectrum of compound **15**

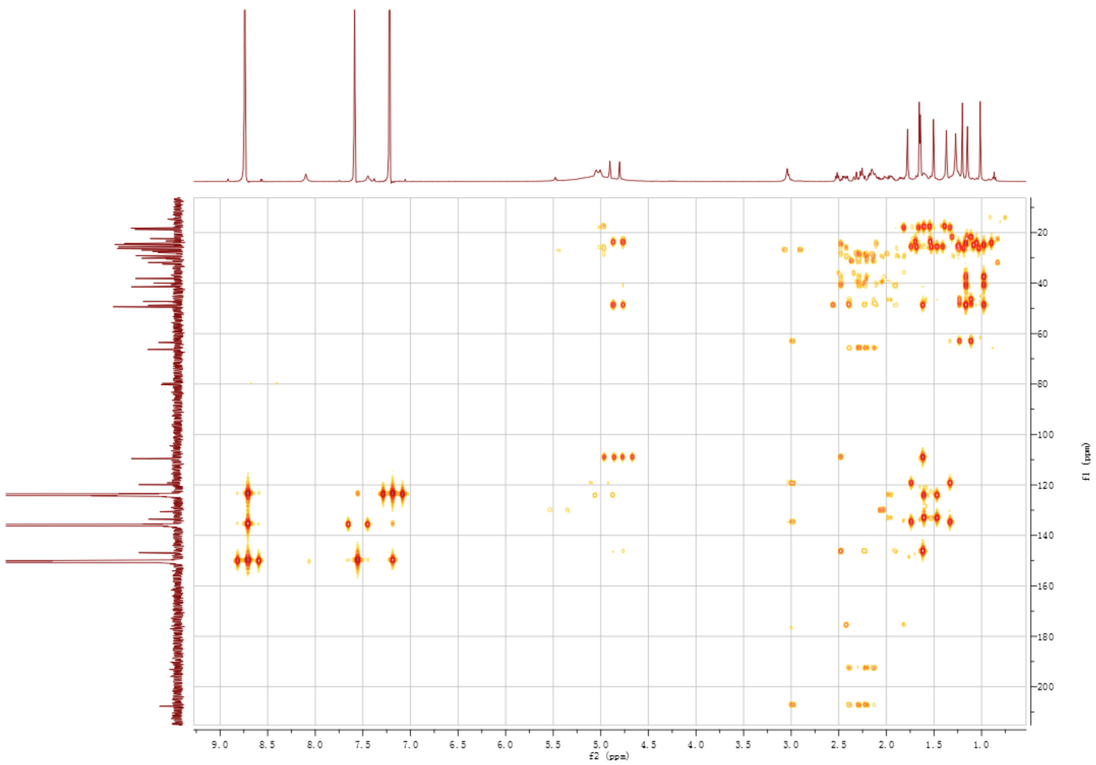

Figure S50. HMBC spectrum of compound **15**

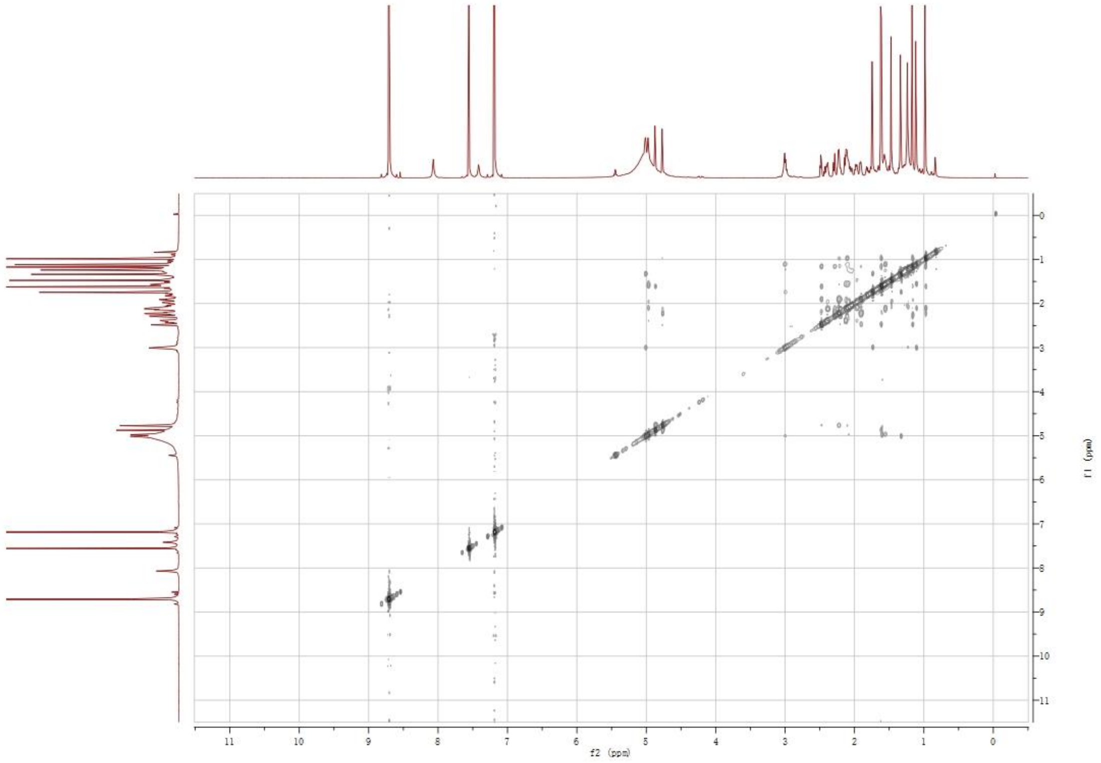

Figure S51. ROESY spectrum of compound **15**

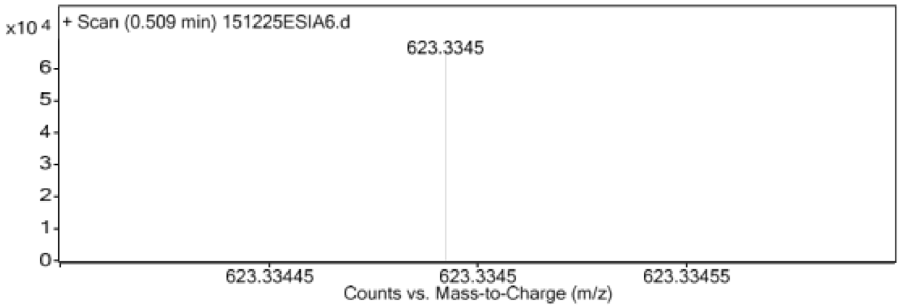

Peak List

m/z	z	Abund	Formula	Ion
121.0509		48542.58		
232.1125	1	265155.63		
233.1153	1	42027.89		
601.352	1	28641.88		
623.3345	1	66076.87	C38 H48 Na O6	M+
922.0098	1	59309.74		
1223.6797	1	85734.8		
1224.6832	1	69575.97		
1225.6855	1	26548.52		
1825.0274	1	26869.52		

Formula Calculator Element Limits

Element	Min	Max
C	0	200
H	0	400
O	0	9
Na	1	1

Formula Calculator Results

Formula	CalculatedMass	Mz	Diff.(mDa)	Diff. (ppm)	DBE
C38 H48 Na O6	623.3349	623.3345	0.4	0.6	14.5

Figure S52. HR-ESI-MS spectrum of compound **15**

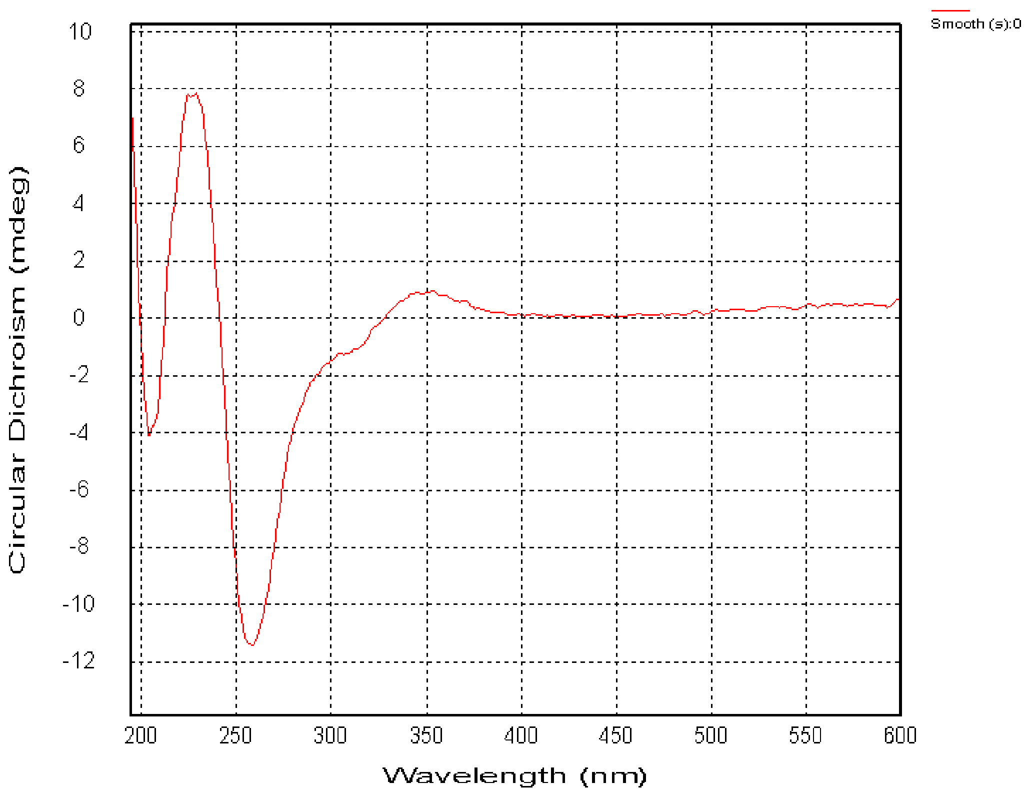

Figure S53. CD spectrum of compound **15**

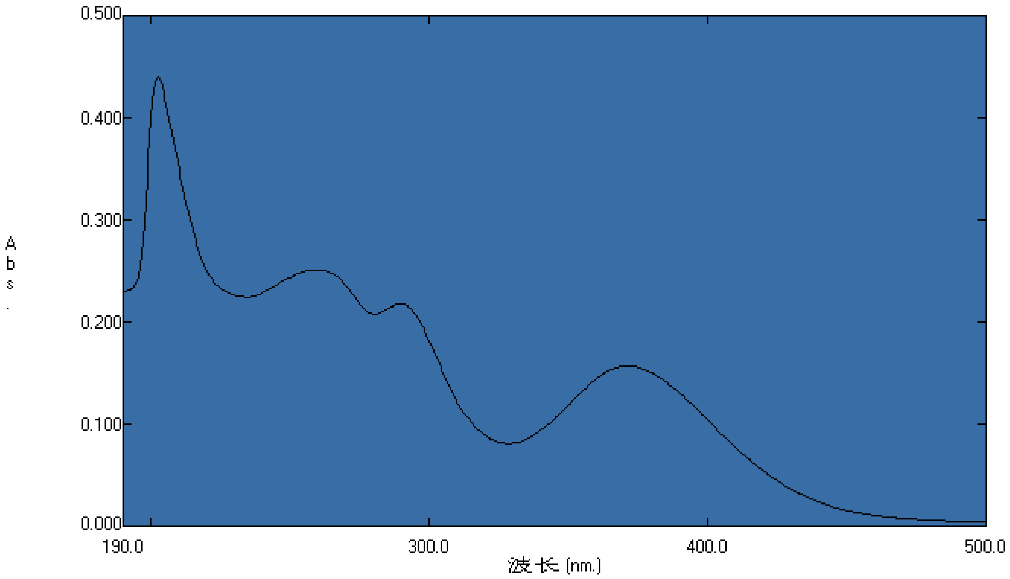

Figure S54. UV spectrum of compound **15**

1. Computational methods

1.1 Conformational analysis

Conformational analysis for compound **3-15** were performed in Yinfo Cloud Platform (http://cloud.yinfotek.com/) using Stochastic or Custom algorithm by Confab [1] at MMFF94 force field with RMSD threshold of 0.5 Å and energy window of 7 kcal/mol.

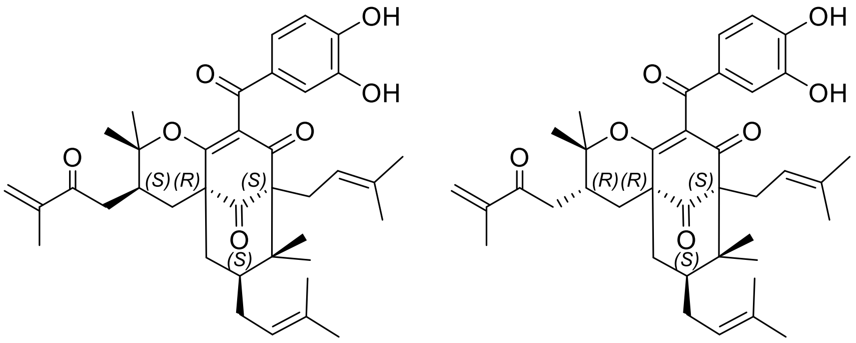

3a **3b**

Figure S55. Chemical structure of compound **3**.

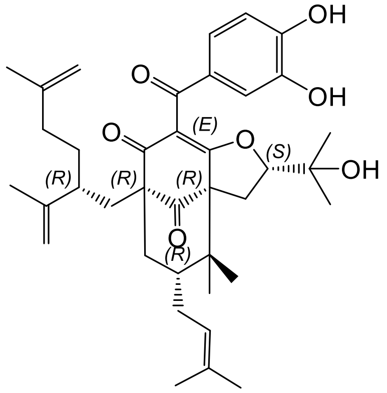

5a

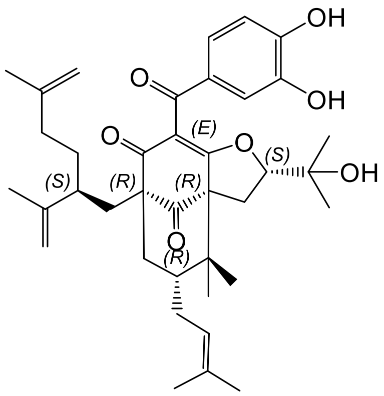

5b

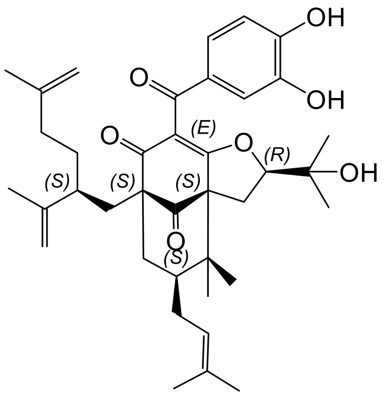

5a'

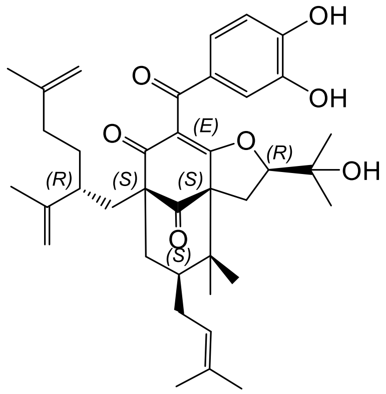

5b'

Figure S56. Chemical structure of compound **5**

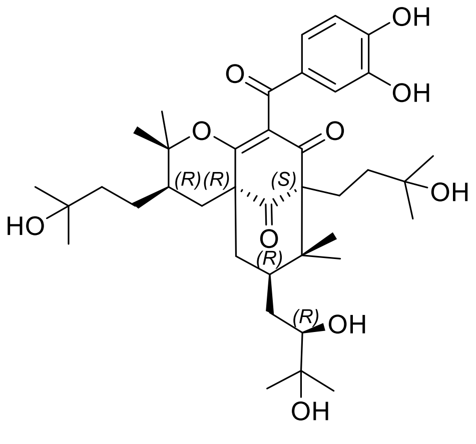

7a

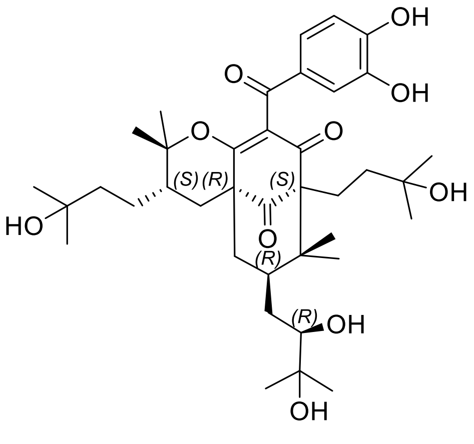

7b

Figure S57. Chemical structure of compound **7**

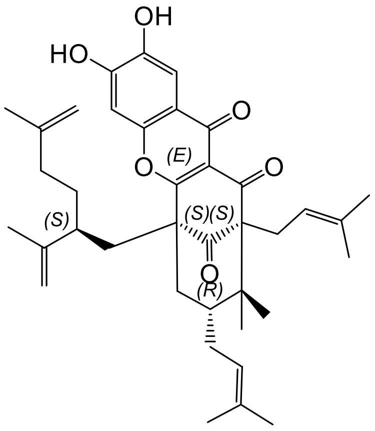

11a

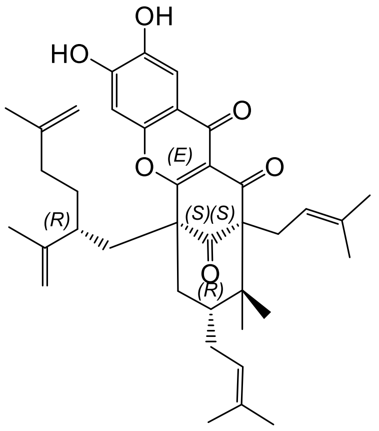

11b

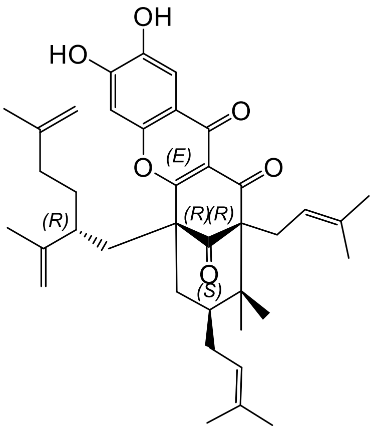

11a'

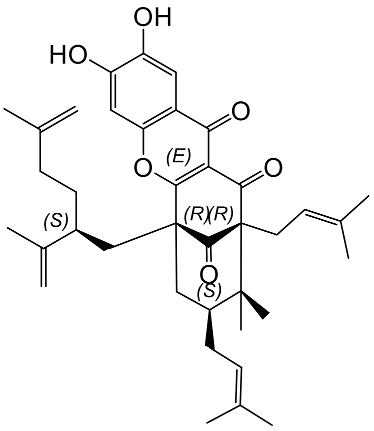

11b'

Figure S58. Chemical structure of compound **11**

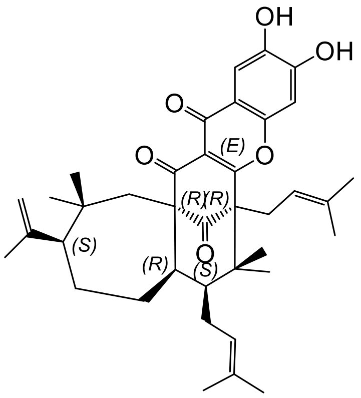

15a

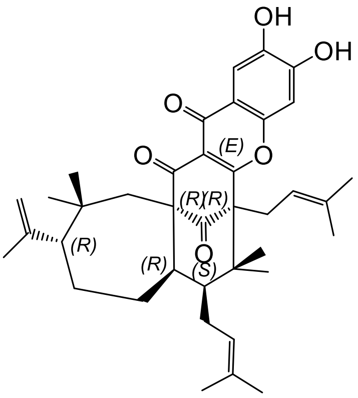

15b

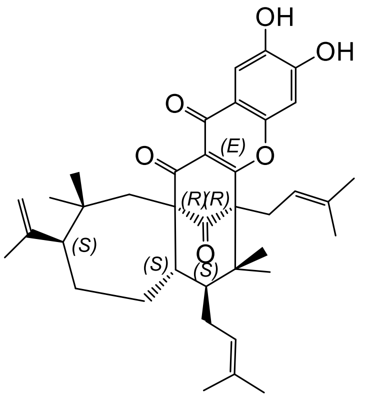

15c

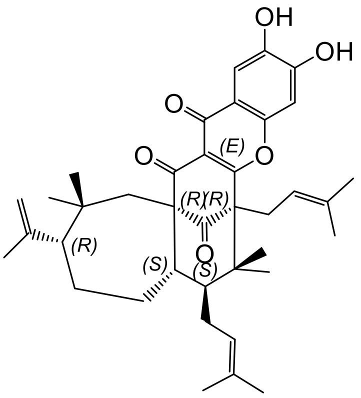

15d

Figure S59. Chemical structure of compound **15**

1.2 NMR calculation

The theoretical calculations were carried out using Gaussian 09 [2]. At first, all conformers were optimized at PM6. Room-temperature equilibrium populations were calculated according to Boltzmann distribution law), based on which dominative conformers of population over 1% were kept. The chosen conformers were further optimized at B3LYP/6-31G(d,p) in gas phase. Vibrational frequency analysis confirmed the stable structures. NMR calculations were carried out following the protocol adapted from Michael *et al.* [3] using the Gauge-Including Atomic Orbitals (GIAO) method at mPW1PW91/6-311+G(2d,p) level in $CDCl_3$ simulated by the IEFPCM model. The TMS-corrected NMR chemical shift values were averaged according to Boltzmann distribution and fitted to the experimental values by linear

regression. The calculated ^{13}C- and ^{1}H-NMR chemical shift values of TMS in (CH3)$_2$CO were 207.07, 30.92 and 2.17 ppm respectively. To confirm the conclusions of NMR calculations, DP4+ analysis was also performed.

$$\frac{N_i}{N} = \frac{g_i e^{-\frac{E_i}{k_B T}}}{\sum g_i e^{-\frac{E_i}{k_B T}}} \quad \textbf{(1)}$$

where N_i is the number of conformer i with energy E_i and degeneracy g_i at temperature *T*, and k_B is Boltzmann constant.

1.3 ECD calculation

The structures were directly derived from the previous NMR calculations. ECD calculations were conducted at B3LYP/6-311G(d,p) level in methanol with IEFPCM model using Time-dependent Density functional theory (TD-DFT). Rotatory strengths for 30 excited states were calculated. The ECD spectrum was simulated using the ECD/UV analysis tool in Yinfo Cloud Platform (https://cloud.yinfotek.com/) by overlapping Gaussian functions for each transition according to (2).

$$\Delta\varepsilon(E) = \frac{1}{2.297\times10^{-39}} \times \frac{1}{\sqrt{2\pi\sigma}} \sum_{i}^{A} \Delta E_i R_i e^{-\left(\frac{E-E_i}{2\sigma}\right)^2} \quad \textbf{(2)}$$

where σ represents the width of the band at 1/*e* height, while ΔE_i and R_i are the excitation energies and rotatory strengths for transition *i*, respectively.

The spectrum of the enantiomers were produced directly by mirror inversion about the horizontal axis.

1.4 References

1. Noel M OBoyle, Tim V, ermeersch, Christopher J Flynn, Anita R Maguire Maguire, and Geoffey R Hutchison. Confab - systematic generation of diverse low-energy conformers. *Journal of Cheminformatics*, 3:3–8, March 2011.

2. M. J. Frisch, G. W. Trucks, H. B. Schlegel, G. E. Scuseria, M. A. Robb, J. R. Cheeseman, G. Scalmani, V. Barone, B. Mennucci, G. A. Petersson, H. Nakatsuji, M. Caricato, X. Li, H. P. Hratchian, A. F. Izmaylov, J. Bloino, G. Zheng, J. L. Sonnenberg, M. Hada, M. Ehara, K. Toyota, R. Fukuda, J. Hasegawa, M. Ishida, T. Nakajima, Y. Honda, O. Kitao, H. Nakai, T. Vreven, J. A. Montgomery, Jr., J. E. Peralta, F. Ogliaro, M. Bearpark, J. J. Heyd, E. Brothers, K. N. Kudin, V. N. Staroverov, R. Kobayashi, J. Normand, K. Raghavachari, A. Rendell, J. C. Burant, S. S. Iyengar, J. Tomasi, M. Cossi, N. Rega, J. M. Millam, M. Klene, J. E. Knox, J. B. Cross, V. Bakken, C. Adamo, J. Jaramillo, R. Gomperts, R. E. Stratmann, O. Yazyev, A. J. Austin, R. Cammi, C. Pomelli, J. W. Ochterski, R. L. Martin, K. Morokuma, V. G. Zakrzewski, G. A. Voth, P. Salvador, J. J. Dannenberg, S. Dapprich, A. D. Daniels, O. Farkas, J. B. Foresman, J. V. Ortiz, J. Cioslowski, and D. J. Fox. Gaussian 09 Revision D.01. Gaussian Inc. Wallingford CT 2009.
3. Michael W. Lodewyk, Matthew R. Siebert, and Dean J. Tantillo. Computational Prediction of 1H and 13C Chemical Shifts: A Useful Tool for Natural Product, Mechanistic, and Synthetic Organic Chemistry. *Chem. Rev.*, 2012, *112* (3), pp 1839–1862.

2. Energies and Coordinates

2.1 Energies at B3LYP theory level

Table S1. Energies of configurations **3-15**

Configuration	Conformer	Structure	E (Hartree)	E (kcal/mol)	Population (%)
3a	1	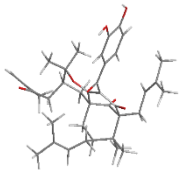	-2003.44644560	-1257181.62	92.1
3a	2	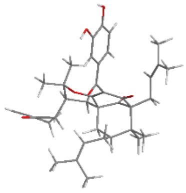	-2003.44390520	-1257180.02	6.2
3a	3	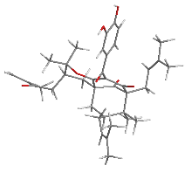	-2003.44271410	-1257179.27	1.7
3b	1	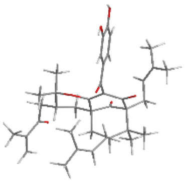	-2003.45242160	-1257185.37	91.3
3b	2	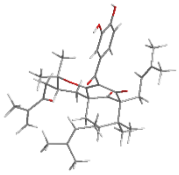	-2003.44988080	-1257183.77	6.1
3b	3	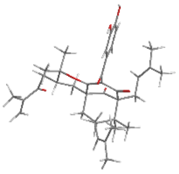	-2003.44903000	-1257183.24	2.5

5a	1	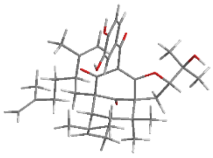	-1992.07050250	-1250043.10	100.0
5b	1	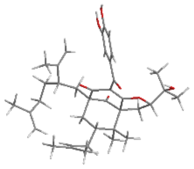	-2004.64733410	-1257935.18	64.7
5b	2	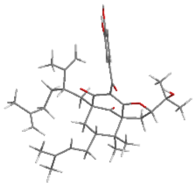	-2004.64675160	-1257934.82	35.3
7a	1	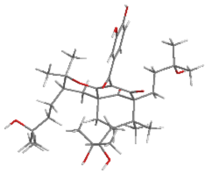	-2233.97168530	-1401838.39	61.2
7a	2	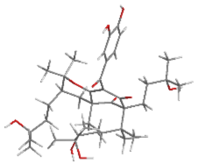	-2233.97126550	-1401838.12	38.8
7b	2	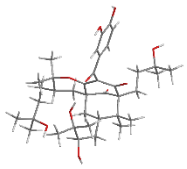	-2233.98081510	-1401844.12	100.0
11a	1	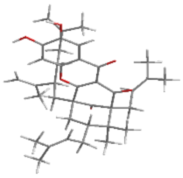	-1928.21805330	-1209975.09	76.1

11a	2	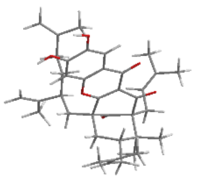	-1928.21689230	-1209974.36	22.2
11a	3	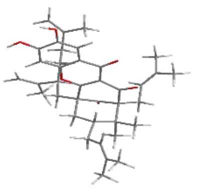	-1928.21443940	-1209972.82	1.6
11b	1	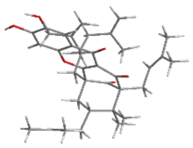	-1928.22034100	-1209976.52	78.0
11b	2	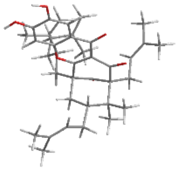	-1928.21914390	-1209975.77	22.0
15a	1	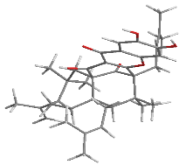	-1928.32490450	-1210042.14	99.0
15a	9	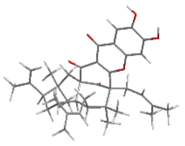	-1928.32052220	-1210039.39	1.0
15b	2	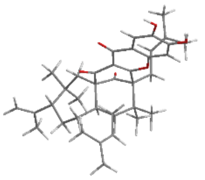	-1928.32976750	-1210045.19	66.6

15b	1	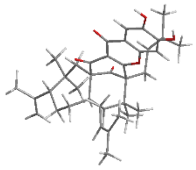	-1928.32911680	-1210044.78	33.4
15c	1	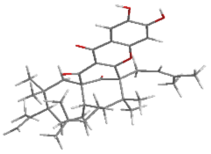	-1928.32229170	-1210040.50	93.2
15c	2	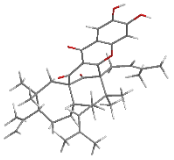	-1928.31982180	-1210038.95	6.8
15d	1	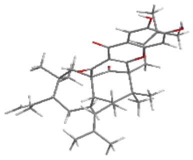	-1928.32117400	-1210039.80	100.0

2.2 Coordinates at B3LYP theory level

Table S2. Standard orientations of configurations **3-15**

Conformer 3a-1

Center	Atomic	Atomic	Coordinates (Angstroms)		
Number	Number	Type	X	Y	Z
1	6	0	1.115804	0.920099	-0.867602
2	6	0	-0.848091	2.557904	-0.559302
3	6	0	-1.479694	1.516106	0.394998
4	6	0	-0.885698	0.179004	0.485598
5	6	0	0.316602	-0.103199	-0.076802

6	6	0	-3.292306	-2.562189	1.741198
7	1	0	-2.928406	-2.626590	2.763498
8	6	0	-2.685203	-1.636090	0.870798
9	6	0	-1.573701	-0.812794	1.402398
10	6	0	-4.324508	-3.361986	1.292398
11	6	0	-4.779408	-3.262184	-0.040102
12	6	0	-4.183005	-2.345586	-0.903902
13	1	0	-4.547705	-2.279785	-1.923902
14	6	0	-3.142303	-1.535689	-0.450202
15	1	0	-2.695601	-0.812990	-1.124502
16	8	0	-1.198101	-0.915895	2.563798
17	8	0	-4.987111	-4.299784	2.052498
18	1	0	-4.642911	-4.285985	2.958398
19	8	0	-5.791110	-4.054181	-0.475502
20	1	0	-6.072712	-4.608881	0.273398
21	8	0	-2.477593	1.802709	1.048298
22	8	0	0.862898	-1.314501	0.164498
23	6	0	0.098807	1.862702	-1.531002
24	8	0	0.109208	2.075301	-2.728802
25	6	0	-2.003589	3.272208	-1.322702
26	1	0	-1.556087	4.014206	-1.991602
27	1	0	-2.611087	3.807309	-0.593302
28	6	0	-5.025791	2.536916	-0.847602
29	1	0	-5.530693	1.685518	-0.372502

30	1	0	-5.817589	3.205419	-1.215402
31	1	0	-4.461489	3.051215	-0.069302
32	6	0	-4.163092	2.057214	-1.988802
33	6	0	-2.865891	2.361010	-2.164602
34	1	0	-2.366693	1.952209	-3.041202
35	6	0	-4.883395	1.183416	-2.990102
36	1	0	-5.742993	1.711618	-3.426902
37	1	0	-5.287997	0.282417	-2.507902
38	1	0	-4.227896	0.871214	-3.809702
39	6	0	1.987807	1.822796	0.081098
40	1	0	2.652609	2.385894	-0.585302
41	1	0	2.631205	1.188694	0.696198
42	6	0	0.075612	3.576502	0.279698
43	6	0	1.246410	2.816098	1.012398
44	1	0	1.983412	3.608196	1.209998
45	6	0	0.969208	2.217799	2.427598
46	1	0	0.379810	2.945501	2.998398
47	6	0	2.265708	1.992895	3.167798
48	1	0	2.896410	2.883893	3.232598
49	1	0	0.372706	1.306601	2.391398
50	6	0	1.584296	-2.027403	-0.900802
51	6	0	2.660099	-1.084606	-1.499602
52	1	0	3.026897	-1.577007	-2.410102
53	6	0	1.984702	0.231296	-1.947302

54	1	0	1.351702	0.039998	-2.817402
55	1	0	2.744005	0.939594	-2.296002
56	6	0	0.549395	-2.459200	-1.952502
57	1	0	0.047497	-1.609398	-2.423002
58	1	0	-0.217807	-3.078798	-1.479002
59	1	0	1.031793	-3.048501	-2.740002
60	6	0	3.881999	-0.837509	-0.562602
61	1	0	3.577599	-0.835509	0.486498
62	1	0	4.289402	0.155489	-0.784702
63	6	0	2.138692	-3.265604	-0.200202
64	1	0	2.666891	-3.900206	-0.919502
65	1	0	1.310491	-3.841002	0.224298
66	1	0	2.834793	-3.013006	0.600098
67	6	0	0.731115	4.594900	-0.689602
68	1	0	1.355617	5.288398	-0.114802
69	1	0	-0.019183	5.193302	-1.212802
70	1	0	1.364014	4.126898	-1.449302
71	6	0	-0.764385	4.393304	1.282198
72	1	0	-1.395987	3.769006	1.915898
73	1	0	-1.413883	5.102706	0.759998
74	1	0	-0.096384	4.981102	1.923498
75	6	0	5.015396	-1.852213	-0.707202
76	6	0	5.845097	-1.831915	-1.971302
77	6	0	6.581593	-2.919117	-2.239502

78	1	0	6.575991	-3.767217	-1.562402
79	1	0	7.204193	-2.983919	-3.127902
80	6	0	5.838700	-0.617215	-2.870002
81	1	0	4.841401	-0.390012	-3.265302
82	1	0	6.506100	-0.767417	-3.723702
83	1	0	6.180603	0.278784	-2.335802
84	8	0	5.247494	-2.660313	0.178998
85	6	0	2.733704	0.878094	3.753298
86	6	0	4.069604	0.892690	4.461398
87	1	0	4.749002	0.138888	4.038998
88	1	0	3.953504	0.640490	5.524998
89	1	0	4.561207	1.868889	4.396698
90	6	0	2.020601	-0.450904	3.798698
91	1	0	1.870500	-0.761604	4.842598
92	1	0	2.637998	-1.234106	3.335398
93	1	0	1.044900	-0.453501	3.310698

Conformer 3a-2

Center	Atomic	Atomic	Coordinates (Angstroms)		
Number	Number	Type	X	Y	Z
1	6	0	1.130501	0.661200	-0.980004
2	6	0	-0.749798	2.417501	-0.880504
3	6	0	-1.444899	1.513101	0.166796
4	6	0	-0.902299	0.177901	0.435896
5	6	0	0.283400	-0.225300	-0.083904

6	6	0	-3.394900	-2.254898	2.099496
7	1	0	-2.995200	-2.215398	3.109696
8	6	0	-2.777600	-1.473798	1.103496
9	6	0	-1.606400	-0.652999	1.491296
10	6	0	-4.481501	-3.046697	1.784496
11	6	0	-4.982701	-3.082097	0.465496
12	6	0	-4.376301	-2.308497	-0.522204
13	1	0	-4.777201	-2.345097	-1.530004
14	6	0	-3.279900	-1.507598	-0.204204
15	1	0	-2.824100	-0.895798	-0.975304
16	8	0	-1.184000	-0.629099	2.640396
17	8	0	-5.159401	-3.850597	2.674196
18	1	0	-4.781201	-3.744597	3.560196
19	8	0	-6.048901	-3.865097	0.162996
20	1	0	-6.328001	-4.309496	0.982896
21	8	0	-2.445098	1.910302	0.755996
22	8	0	0.778800	-1.421800	0.302496
23	6	0	0.171101	1.576201	-1.756604
24	8	0	0.206701	1.659201	-2.969904
25	6	0	-1.856698	3.102501	-1.736404
26	1	0	-1.363398	3.739101	-2.478304
27	1	0	-2.440498	3.748302	-1.081404
28	6	0	-4.924598	2.598703	-1.237604
29	1	0	-5.465299	1.840603	-0.655804

30	1	0	-5.687398	3.238503	-1.704604
31	1	0	-4.347798	3.195403	-0.530804
32	6	0	-4.073298	1.934903	-2.291704
33	6	0	-2.759598	2.146002	-2.479304
34	1	0	-2.273399	1.605502	-3.289404
35	6	0	-4.826899	0.979403	-3.188404
36	1	0	-5.650199	1.491303	-3.707004
37	1	0	-5.287299	0.170603	-2.603404
38	1	0	-4.178499	0.529603	-3.947404
39	6	0	2.024701	1.619600	-0.114904
40	1	0	2.707602	2.101999	-0.824804
41	1	0	2.649201	1.029299	0.559196
42	6	0	0.215802	3.475800	-0.133004
43	6	0	1.316602	2.726900	0.707396
44	1	0	2.106902	3.474200	0.865096
45	6	0	0.903302	2.323900	2.165396
46	1	0	0.765902	3.255000	2.723996
47	6	0	1.901801	1.441800	2.877396
48	1	0	1.709101	0.372300	2.791796
49	1	0	-0.060199	1.814201	2.199096
50	6	0	1.461500	-2.281100	-0.676604
51	6	0	2.585400	-1.465501	-1.366304
52	1	0	2.928400	-2.074601	-2.213304
53	6	0	1.980901	-0.176000	-1.963404

54	1	0	1.352700	-0.428400	-2.821004
55	1	0	2.778001	0.457199	-2.368004
56	6	0	0.406399	-2.768500	-1.683504
57	1	0	-0.055200	-1.948799	-2.241104
58	1	0	-0.388701	-3.299599	-1.152204
59	1	0	0.860199	-3.456900	-2.404704
60	6	0	3.817700	-1.180201	-0.452504
61	1	0	3.517200	-1.077501	0.592696
62	1	0	4.260600	-0.226401	-0.760104
63	6	0	1.952199	-3.465100	0.153096
64	1	0	2.450799	-4.195501	-0.492704
65	1	0	1.096099	-3.952400	0.629496
66	1	0	2.658499	-3.165301	0.927796
67	6	0	0.938103	4.351700	-1.188404
68	1	0	1.603103	5.056300	-0.675704
69	1	0	0.228803	4.943400	-1.773204
70	1	0	1.542202	3.775800	-1.895204
71	6	0	-0.596097	4.434201	0.761696
72	1	0	-1.242198	3.912101	1.468696
73	1	0	-1.226197	5.094401	0.158096
74	1	0	0.092203	5.075401	1.325796
75	6	0	4.908200	-2.248902	-0.512904
76	6	0	5.742499	-2.355102	-1.770204
77	6	0	6.448899	-3.480402	-1.945904

78	1	0	6.417299	-4.270402	-1.202404
79	1	0	7.071899	-3.633903	-2.823104
80	6	0	5.771800	-1.218002	-2.764704
81	1	0	4.780800	-0.993802	-3.176804
82	1	0	6.432200	-1.457302	-3.603504
83	1	0	6.141700	-0.292102	-2.305304
84	8	0	5.100899	-3.000402	0.430496
85	6	0	2.958701	1.824499	3.612596
86	6	0	3.846301	0.805599	4.287296
87	1	0	4.889201	0.899998	3.952196
88	1	0	3.516600	-0.218801	4.087896
89	1	0	3.857701	0.952199	5.376696
90	6	0	3.356202	3.261399	3.850896
91	1	0	3.391402	3.481899	4.926996
92	1	0	2.676103	3.978399	3.383696
93	1	0	4.366402	3.456299	3.463896

Conformer 3a-3

Center	Atomic	Atomic	Coordinates (Angstroms)		
Number	Number	Type	X	Y	Z
1	6	0	1.427605	0.512597	-0.967797
2	6	0	-0.672387	1.871809	-1.579397
3	6	0	-1.403192	1.142014	-0.428097
4	6	0	-0.727298	0.041410	0.265003
5	6	0	0.574400	-0.254598	0.029303

6	6	0	-3.011812	-2.413277	2.174903
7	1	0	-2.853310	-2.022778	3.177103
8	6	0	-2.359509	-1.781481	1.098503
9	6	0	-1.491502	-0.618186	1.397703
10	6	0	-3.831919	-3.500572	1.946203
11	6	0	-4.026621	-3.986871	0.635403
12	6	0	-3.387618	-3.360875	-0.433197
13	1	0	-3.554520	-3.743574	-1.434897
14	6	0	-2.558211	-2.263880	-0.202097
15	1	0	-2.078809	-1.770482	-1.040897
16	8	0	-1.378099	-0.170187	2.531503
17	8	0	-4.513323	-4.196668	2.919603
18	1	0	-4.362720	-3.780769	3.782003
19	8	0	-4.832628	-5.056466	0.418603
20	1	0	-5.187929	-5.335764	1.280903
21	8	0	-2.540790	1.473520	-0.110397
22	8	0	1.161695	-1.207001	0.788003
23	6	0	0.493608	1.024803	-2.073697
24	8	0	0.726107	0.820101	-3.250397
25	6	0	-1.701286	2.116815	-2.723897
26	1	0	-1.183783	2.635612	-3.537097
27	1	0	-2.476982	2.782220	-2.346397
28	6	0	-4.693191	1.158733	-2.477597
29	1	0	-5.184595	0.473336	-1.774297

30	1	0	-5.468089	1.494837	-3.181597
31	1	0	-4.344186	2.014931	-1.900297
32	6	0	-3.586296	0.443426	-3.211697
33	6	0	-2.310593	0.857319	-3.294497
34	1	0	-1.619597	0.245015	-3.870797
35	6	0	-4.019503	-0.829571	-3.902497
36	1	0	-4.826302	-0.633166	-4.622897
37	1	0	-4.421807	-1.552669	-3.178697
38	1	0	-3.194006	-1.305976	-4.441497
39	6	0	2.034812	1.804294	-0.313097
40	1	0	2.734915	2.207989	-1.055297
41	1	0	2.637311	1.516490	0.552903
42	6	0	-0.008879	3.237005	-1.019797
43	6	0	1.047519	2.924699	0.109003
44	1	0	1.675124	3.826096	0.152603
45	6	0	0.511318	2.746802	1.566203
46	1	0	-0.440985	2.221408	1.598603
47	6	0	0.420925	4.039903	2.341403
48	1	0	1.225630	4.752198	2.142003
49	1	0	1.213714	2.082698	2.092703
50	6	0	2.096389	-2.159107	0.170303
51	6	0	3.197894	-1.366313	-0.580197
52	1	0	3.745490	-2.102516	-1.183397
53	6	0	2.530800	-0.388309	-1.574497

54	1	0	2.087796	-0.951007	-2.399997
55	1	0	3.291203	0.248686	-2.039097
56	6	0	1.291884	-3.057702	-0.782797
57	1	0	0.827187	-2.495099	-1.596797
58	1	0	0.495581	-3.563297	-0.228997
59	1	0	1.943679	-3.819406	-1.224197
60	6	0	4.223998	-0.658819	0.357103
61	1	0	3.748000	-0.332516	1.285103
62	1	0	4.595803	0.236979	-0.153397
63	6	0	2.600084	-2.992710	1.346203
64	1	0	3.289080	-3.766214	0.991203
65	1	0	1.750381	-3.483305	1.830503
66	1	0	3.124488	-2.390413	2.088903
67	6	0	0.731125	3.957001	-2.175997
68	1	0	1.199930	4.869998	-1.791197
69	1	0	0.039327	4.257305	-2.967397
70	1	0	1.512721	3.347997	-2.639697
71	6	0	-1.089974	4.205612	-0.503097
72	1	0	-1.735276	3.756316	0.252303
73	1	0	-1.724372	4.555115	-1.323597
74	1	0	-0.610268	5.084709	-0.059997
75	6	0	5.431193	-1.514726	0.740103
76	6	0	6.459391	-1.827332	-0.324097
77	6	0	7.308585	-2.834937	-0.079697

78	1	0	7.250082	-3.388837	0.851803
79	1	0	8.075884	-3.122442	-0.793597
80	6	0	6.516096	-1.011633	-1.594697
81	1	0	5.584296	-1.062927	-2.170197
82	1	0	7.324294	-1.364237	-2.242297
83	1	0	6.701102	0.049466	-1.382597
84	8	0	5.562791	-1.935827	1.879003
85	6	0	-0.491573	4.386608	3.262403
86	6	0	-0.405865	5.720308	3.967203
87	1	0	-1.319961	6.310013	3.807703
88	1	0	0.445839	6.314903	3.621003
89	1	0	-0.309166	5.589007	5.054403
90	6	0	-1.653978	3.523715	3.691503
91	1	0	-1.622479	3.359915	4.777903
92	1	0	-1.675783	2.542115	3.213703
93	1	0	-2.608475	4.028421	3.484303

Conformer 3b-1

Center	Atomic	Atomic	Coordinates (Angstroms)		
Number	Number	Type	X	Y	Z
1	6	0	1.219500	0.684897	-0.660389
2	6	0	-0.543702	2.526595	-0.348689
3	6	0	-1.358201	1.510794	0.488911
4	6	0	-0.939299	0.107294	0.513311
5	6	0	0.261001	-0.281005	0.009511

6	6	0	-3.770396	-2.337609	1.453811
7	1	0	-3.490996	-2.504809	2.491011
8	6	0	-2.978597	-1.468708	0.677811
9	6	0	-1.797098	-0.846207	1.320011
10	6	0	-4.873596	-2.953311	0.896711
11	6	0	-5.215496	-2.723511	-0.453389
12	6	0	-4.434697	-1.864710	-1.224589
13	1	0	-4.713897	-1.697010	-2.259689
14	6	0	-3.323298	-1.238909	-0.660789
15	1	0	-2.730198	-0.559108	-1.262789
16	8	0	-1.499998	-1.086207	2.484611
17	8	0	-5.716695	-3.818212	1.559111
18	1	0	-5.446695	-3.887311	2.487311
19	8	0	-6.298795	-3.334912	-0.994889
20	1	0	-6.712095	-3.872113	-0.295989
21	8	0	-2.352101	1.877392	1.108411
22	8	0	0.644103	-1.556604	0.216411
23	6	0	0.368599	1.781496	-1.313289
24	8	0	0.476998	2.057096	-2.492689
25	6	0	-1.553503	3.428793	-1.119689
26	1	0	-0.977904	4.140994	-1.719689
27	1	0	-2.126604	3.997793	-0.388189
28	6	0	-4.669003	3.092990	-0.851189
29	1	0	-5.306002	2.296089	-0.445089

30	1	0	-5.345704	3.874389	-1.226289
31	1	0	-4.089903	3.496890	-0.020389
32	6	0	-3.810102	2.553091	-1.967989
33	6	0	-2.476002	2.685692	-2.056689
34	1	0	-1.982502	2.254693	-2.925889
35	6	0	-4.578701	1.832590	-3.051889
36	1	0	-5.329102	2.493589	-3.508589
37	1	0	-5.131500	0.976489	-2.640189
38	1	0	-3.921601	1.467791	-3.848289
39	6	0	2.139999	1.397698	0.393911
40	1	0	2.913199	1.908299	-0.191789
41	1	0	2.656700	0.642298	0.995411
42	6	0	0.445497	3.364396	0.608211
43	6	0	1.468798	2.418097	1.350611
44	1	0	2.280997	3.098998	1.643811
45	6	0	1.026099	1.771796	2.700111
46	1	0	0.478398	2.525196	3.279311
47	6	0	2.230999	1.356698	3.508911
48	1	0	2.955298	2.160699	3.665711
49	1	0	0.338500	0.937496	2.562311
50	6	0	1.523304	-2.297303	-0.701289
51	6	0	2.655003	-1.359802	-1.187189
52	1	0	3.267402	-1.140501	-0.301089
53	6	0	2.068801	-0.043602	-1.716589

54	1	0	1.455801	-0.232803	-2.603989
55	1	0	2.868100	0.618499	-2.058789
56	6	0	0.635504	-2.844504	-1.826189
57	1	0	0.218103	-2.045305	-2.445489
58	1	0	-0.197895	-3.404505	-1.391789
59	1	0	1.201205	-3.519303	-2.476589
60	6	0	3.573503	-2.029601	-2.247589
61	1	0	3.055403	-2.070501	-3.210289
62	1	0	3.816105	-3.052400	-1.944289
63	6	0	2.040605	-3.436402	0.179911
64	1	0	2.643406	-4.142402	-0.398589
65	1	0	1.196406	-3.982603	0.610911
66	1	0	2.646904	-3.044202	1.002911
67	6	0	1.281396	4.350197	-0.250689
68	1	0	1.940795	4.928397	0.406811
69	1	0	0.644795	5.064396	-0.779689
70	1	0	1.907696	3.854297	-0.997989
71	6	0	-0.350004	4.216795	1.616611
72	1	0	-1.088803	3.637894	2.173111
73	1	0	-0.878305	5.027794	1.106111
74	1	0	0.341895	4.681196	2.330011
75	6	0	4.849402	-1.217799	-2.464589
76	6	0	6.052103	-1.484298	-1.589889
77	6	0	7.086502	-0.638296	-1.696689

78	1	0	7.042901	0.194103	-2.391489
79	1	0	7.988302	-0.758995	-1.102589
80	6	0	6.067804	-2.663798	-0.646489
81	1	0	5.246004	-2.626499	0.079211
82	1	0	7.005104	-2.693197	-0.083189
83	1	0	5.975205	-3.615998	-1.184689
84	8	0	4.870201	-0.341399	-3.316089
85	6	0	2.519501	0.167498	4.062711
86	6	0	3.789401	-0.012100	4.863611
87	1	0	3.564001	-0.301601	5.899911
88	1	0	4.393000	0.900900	4.891511
89	1	0	4.410302	-0.818700	4.447611
90	6	0	1.653102	-1.065703	3.984711
91	1	0	1.369703	-1.392403	4.995511
92	1	0	2.215403	-1.901902	3.543311
93	1	0	0.734602	-0.933604	3.410911

Conformer 3b-2

Center	Atomic	Atomic	Coordinates (Angstroms)		
Number	Number	Type	X	Y	Z
1	6	0	1.196501	0.431404	-0.809905
2	6	0	-0.541903	2.323101	-0.883005
3	6	0	-1.386701	1.497899	0.116895
4	6	0	-0.964998	0.137000	0.460895
5	6	0	0.236103	-0.352798	0.059895

6	6	0	-3.804793	-1.963906	2.012595
7	1	0	-3.469893	-1.934706	3.046495
8	6	0	-3.036095	-1.295605	1.040195
9	6	0	-1.803796	-0.602102	1.484395
10	6	0	-4.956792	-2.631709	1.646495
11	6	0	-5.371992	-2.655210	0.297795
12	6	0	-4.613593	-1.995508	-0.667405
13	1	0	-4.949493	-2.021309	-1.699005
14	6	0	-3.452595	-1.317806	-0.297405
15	1	0	-2.876996	-0.792704	-1.052305
16	8	0	-1.442596	-0.611901	2.654995
17	8	0	-5.783390	-3.315911	2.510795
18	1	0	-5.463691	-3.206610	3.418995
19	8	0	-6.504190	-3.315312	-0.055005
20	1	0	-6.891290	-3.687413	0.757095
21	8	0	-2.396702	1.982997	0.617695
22	8	0	0.629706	-1.543297	0.557495
23	6	0	0.367799	1.389403	-1.673705
24	8	0	0.494699	1.430803	-2.882305
25	6	0	-1.517704	3.077399	-1.834505
26	1	0	-0.913905	3.647600	-2.547405
27	1	0	-2.092706	3.789497	-1.243205
28	6	0	-4.644704	2.830392	-1.557205
29	1	0	-5.292902	2.127090	-1.017705

30	1	0	-5.310105	3.531190	-2.082005
31	1	0	-4.072305	3.378193	-0.808405
32	6	0	-3.776802	2.085094	-2.540905
33	6	0	-2.439902	2.184397	-2.631705
34	1	0	-1.942001	1.593898	-3.398405
35	6	0	-4.538900	1.183392	-3.485105
36	1	0	-5.275201	1.754891	-4.068405
37	1	0	-5.108798	0.423791	-2.931405
38	1	0	-3.875499	0.671094	-4.189505
39	6	0	2.121200	1.340406	0.069895
40	1	0	2.863899	1.758308	-0.620505
41	1	0	2.678001	0.719708	0.778695
42	6	0	0.449895	3.316803	-0.077705
43	6	0	1.447797	2.507005	0.835795
44	1	0	2.274896	3.203807	1.030795
45	6	0	0.924098	2.154404	2.269895
46	1	0	0.789796	3.101003	2.801995
47	6	0	1.839400	1.249606	3.060595
48	1	0	1.620602	0.185605	2.969195
49	1	0	-0.058701	1.682102	2.250195
50	6	0	1.458408	-2.487195	-0.205905
51	6	0	2.598906	-1.706593	-0.904905
52	1	0	3.238705	-1.322991	-0.097505
53	6	0	2.033204	-0.513994	-1.690805

54	1	0	1.415704	-0.870595	-2.522005
55	1	0	2.844502	0.049308	-2.158905
56	6	0	0.521009	-3.227397	-1.168505
57	1	0	0.100208	-2.559198	-1.925605
58	1	0	-0.310190	-3.660099	-0.604005
59	1	0	1.045911	-4.038296	-1.683405
60	6	0	3.468408	-2.613291	-1.820205
61	1	0	2.930708	-2.814892	-2.751405
62	1	0	3.668910	-3.568690	-1.327005
63	6	0	1.972110	-3.431994	0.881895
64	1	0	2.546312	-4.257693	0.451995
65	1	0	1.126811	-3.854896	1.432595
66	1	0	2.605809	-2.893393	1.593995
67	6	0	1.292594	4.129305	-1.094305
68	1	0	1.979692	4.787806	-0.550205
69	1	0	0.661192	4.766803	-1.718905
70	1	0	1.888395	3.503306	-1.764505
71	6	0	-0.347707	4.336501	0.760395
72	1	0	-1.061606	3.865000	1.437095
73	1	0	-0.902908	5.024700	0.115995
74	1	0	0.347892	4.943303	1.352795
75	6	0	4.775707	-1.919888	-2.200305
76	6	0	5.991207	-2.104285	-1.321805
77	6	0	7.069805	-1.359483	-1.602805

78	1	0	7.052204	-0.661583	-2.433705
79	1	0	7.983305	-1.432581	-1.018905
80	6	0	5.970709	-3.097785	-0.184305
81	1	0	5.174209	-2.883887	0.538795
82	1	0	6.921609	-3.083183	0.356395
83	1	0	5.809611	-4.122786	-0.542605
84	8	0	4.813005	-1.198988	-3.186405
85	6	0	2.853099	1.606508	3.865995
86	6	0	3.656601	0.566910	4.610995
87	1	0	4.724001	0.626212	4.353295
88	1	0	3.308903	-0.448191	4.394395
89	1	0	3.592601	0.721710	5.697495
90	6	0	3.281096	3.032009	4.119095
91	1	0	3.252395	3.262609	5.193295
92	1	0	2.656894	3.765207	3.601995
93	1	0	4.320196	3.190511	3.797395

Conformer 3b-3

Center	Atomic	Atomic	Coordinates (Angstroms)		
Number	Number	Type	X	Y	Z
1	6	0	-1.497797	0.158706	0.715199
2	6	0	0.293803	1.755606	1.635599
3	6	0	1.243803	1.226705	0.535099
4	6	0	0.814803	0.099806	-0.297801
5	6	0	-0.452897	-0.380094	-0.242001

6	6	0	3.627203	-1.844495	-2.083701
7	1	0	3.547203	-1.371695	-3.059401
8	6	0	2.757503	-1.420495	-1.060501
9	6	0	1.780503	-0.351395	-1.376201
10	6	0	4.556802	-2.835295	-1.836701
11	6	0	4.644902	-3.431795	-0.560401
12	6	0	3.788202	-3.012995	0.455799
13	1	0	3.874902	-3.477295	1.432899
14	6	0	2.849603	-2.012195	0.206499
15	1	0	2.197603	-1.678295	1.006499
16	8	0	1.738903	0.179505	-2.479101
17	8	0	5.452502	-3.328095	-2.760001
18	1	0	5.365602	-2.836295	-3.590501
19	8	0	5.560302	-4.405495	-0.325601
20	1	0	6.065502	-4.537296	-1.147301
21	8	0	2.343703	1.743605	0.367299
22	8	0	-0.815197	-1.305794	-1.155501
23	6	0	-0.765497	0.704906	1.945999
24	8	0	-1.074497	0.365406	3.072099
25	6	0	1.148103	2.067505	2.900599
26	1	0	0.471504	2.429506	3.681099
27	1	0	1.836504	2.875605	2.656099
28	6	0	4.267703	1.615605	2.938399
29	1	0	4.925303	1.081805	2.239399

30	1	0	4.909803	1.995305	3.746299
31	1	0	3.843904	2.461705	2.396999
32	6	0	3.224103	0.676405	3.490199
33	6	0	1.896203	0.877405	3.454299
34	1	0	1.260203	0.117405	3.904399
35	6	0	3.787703	-0.570295	4.133299
36	1	0	4.479903	-0.316995	4.948899
37	1	0	4.367603	-1.158795	3.407899
38	1	0	3.001203	-1.211495	4.544499
39	6	0	-2.253397	1.388306	0.104099
40	1	0	-3.053797	1.625106	0.815699
41	1	0	-2.747797	1.082606	-0.824701
42	6	0	-0.516596	3.043206	1.083199
43	6	0	-1.412896	2.663806	-0.159601
44	1	0	-2.158896	3.469206	-0.213301
45	6	0	-0.733196	2.662206	-1.567301
46	1	0	0.283503	2.275506	-1.540201
47	6	0	-0.766196	4.004106	-2.258701
48	1	0	-1.689296	4.571306	-2.114001
49	1	0	-1.287097	1.944106	-2.191601
50	6	0	-1.792497	-2.366294	-0.870101
51	6	0	-2.987097	-1.754494	-0.096701
52	1	0	-3.479297	-1.066194	-0.798501
53	6	0	-2.489197	-0.949994	1.113499

54	1	0	-2.009497	-1.616994	1.837599
55	1	0	-3.333297	-0.508294	1.648999
56	6	0	-1.052298	-3.469794	-0.104801
57	1	0	-0.722398	-3.132494	0.882099
58	1	0	-0.166398	-3.774294	-0.669501
59	1	0	-1.692598	-4.347094	0.031199
60	6	0	-4.031098	-2.821993	0.336499
61	1	0	-3.646198	-3.386694	1.190899
62	1	0	-4.218098	-3.522993	-0.482101
63	6	0	-2.180598	-2.845694	-2.270101
64	1	0	-2.853398	-3.706694	-2.222501
65	1	0	-1.283398	-3.144594	-2.820001
66	1	0	-2.670997	-2.043894	-2.831701
67	6	0	-1.455496	3.571506	2.198299
68	1	0	-2.037896	4.415006	1.810099
69	1	0	-0.888096	3.940006	3.057199
70	1	0	-2.159096	2.820606	2.570099
71	6	0	0.447004	4.191206	0.724999
72	1	0	1.207904	3.897805	0.001399
73	1	0	0.958304	4.564806	1.617599
74	1	0	-0.122896	5.023806	0.298999
75	6	0	-5.335897	-2.167493	0.787799
76	6	0	-6.410397	-1.881493	-0.235601
77	6	0	-7.466897	-1.162693	0.169799

78	1	0	-7.530797	-0.814193	1.195599
79	1	0	-8.281797	-0.913393	-0.504301
80	6	0	-6.280797	-2.399693	-1.648401
81	1	0	-5.369197	-2.039593	-2.140701
82	1	0	-7.133097	-2.079793	-2.255101
83	1	0	-6.247098	-3.496493	-1.677901
84	8	0	-5.480297	-1.852293	1.959599
85	6	0	0.169904	4.550506	-3.050101
86	6	0	-0.055596	5.902806	-3.685401
87	1	0	0.729204	6.613606	-3.389501
88	1	0	-1.023696	6.331606	-3.406301
89	1	0	-0.015496	5.838006	-4.782001
90	6	0	1.494404	3.910905	-3.390601
91	1	0	1.601304	3.819705	-4.480901
92	1	0	1.628204	2.914205	-2.965001
93	1	0	2.327604	4.543705	-3.052601

Conformer 5a-1

Center	Atomic	Atomic	Coordinates (Angstroms)		
Number	Number	Type	X	Y	Z
1	6	0	1.328561	-0.505109	0.597310
2	6	0	0.277405	1.665000	1.413197
3	6	0	-0.610880	1.590842	0.186828
4	6	0	-0.682119	0.549752	-0.680063
5	6	0	0.202928	-0.601906	-0.476463

6	6	0	1.571385	0.923231	1.057851
7	8	0	2.667420	1.425691	1.218801
8	6	0	0.739241	-1.225707	1.857362
9	6	0	-0.339754	0.956711	2.721395
10	6	0	-0.531952	-0.571466	2.430415
11	6	0	-1.043492	-1.404472	3.642248
12	6	0	-1.503125	-2.788728	3.246767
13	6	0	-2.755386	-3.183397	2.964439
14	6	0	-3.041210	-4.602150	2.533495
15	6	0	-3.973942	-2.294046	3.018393
16	6	0	-1.698258	1.602465	3.060267
17	6	0	0.623277	1.174882	3.909226
18	6	0	2.605768	-1.249651	0.142545
19	6	0	3.503298	-0.590014	-0.942584
20	6	0	-2.651787	-0.254374	-2.180175
21	6	0	-1.494531	0.654798	-1.955356
22	6	0	2.763604	-0.261735	-2.235383
23	6	0	4.750003	-1.468039	-1.221065
24	8	0	0.070513	-1.646511	-1.103352
25	6	0	2.454717	1.007903	-2.529539
26	8	0	-1.191056	1.509125	-2.777958
27	6	0	-3.059200	-1.206229	-1.231523
28	6	0	-4.168653	-2.003986	-1.472944
29	6	0	-4.882561	-1.857280	-2.674667

30	6	0	-4.482747	-0.917538	-3.623648
31	6	0	-3.372614	-0.116923	-3.375631
32	6	0	2.408071	-1.390676	-3.174253
33	6	0	5.771469	-1.514554	-0.064273
34	6	0	7.019969	-2.310520	-0.389712
35	6	0	7.960526	-1.720557	-1.413569
36	6	0	7.279242	-3.478103	0.208648
37	6	0	0.459949	3.196073	1.538667
38	6	0	-0.749443	3.806125	0.804695
39	8	0	-1.306656	2.723359	0.009106
40	6	0	-0.481172	5.022434	-0.115168
41	6	0	0.139197	6.158244	0.714286
42	6	0	0.383910	4.662074	-1.324367
43	8	0	-1.728227	5.458060	-0.669305
44	8	0	-4.569928	-2.925934	-0.544268
45	8	0	-5.963620	-2.692960	-2.813755
46	1	0	1.520083	-1.269848	2.627802
47	1	0	0.518960	-2.256687	1.562858
48	1	0	-1.319126	-0.641494	1.670561
49	1	0	-0.238354	-1.497649	4.383172
50	1	0	-1.857151	-0.870353	4.140539
51	1	0	-0.715601	-3.537573	3.160890
52	1	0	-3.777076	-5.078048	3.197191
53	1	0	-2.137012	-5.219054	2.529159

54	1	0	-3.470623	-4.613797	1.522729
55	1	0	-3.751593	-1.269737	3.328271
56	1	0	-4.455072	-2.254855	2.032618
57	1	0	-4.718706	-2.702654	3.716003
58	1	0	-2.423420	1.476875	2.249058
59	1	0	-2.126099	1.158410	3.963839
60	1	0	-1.593199	2.673220	3.263497
61	1	0	1.623650	0.772864	3.724972
62	1	0	0.740645	2.236829	4.145334
63	1	0	0.225874	0.692043	4.807262
64	1	0	3.220780	-1.398623	1.036886
65	1	0	2.287314	-2.242189	-0.194299
66	1	0	3.859727	0.357610	-0.528274
67	1	0	5.245441	-1.069752	-2.115628
68	1	0	4.445668	-2.494203	-1.467231
69	1	0	2.729015	1.829058	-1.870811
70	1	0	1.906883	1.272503	-3.430089
71	1	0	-2.510074	-1.354178	-0.309515
72	1	0	-5.044576	-0.813747	-4.550174
73	1	0	-3.047722	0.625989	-4.095171
74	1	0	1.799140	-2.150456	-2.672719
75	1	0	3.308370	-1.888670	-3.557372
76	1	0	1.840074	-1.018852	-4.032526
77	1	0	5.304515	-1.941580	0.830083

78	1	0	6.054389	-0.481911	0.191902
79	1	0	8.296047	-0.720136	-1.106769
80	1	0	8.845732	-2.348176	-1.555329
81	1	0	7.474313	-1.599500	-2.390176
82	1	0	8.170960	-4.056296	-0.022339
83	1	0	6.608172	-3.900482	0.953093
84	1	0	1.405122	3.473349	1.065242
85	1	0	0.509164	3.540199	2.572249
86	1	0	-1.535300	4.097926	1.512922
87	1	0	-0.496396	6.415205	1.573416
88	1	0	0.233430	7.049635	0.086753
89	1	0	1.132002	5.899903	1.097551
90	1	0	-0.081193	3.859704	-1.902620
91	1	0	0.481246	5.543475	-1.965286
92	1	0	1.388345	4.347756	-1.022361
93	1	0	-2.288856	5.769301	0.060212
94	1	0	-5.348607	-3.384427	-0.903687
95	1	0	-6.385038	-2.543803	-3.673741

Conformer 5b-1

Center	Atomic	Atomic	Coordinates (Angstroms)		
Number	Number	Type	X	Y	Z
1	6	0	-0.933596	-0.206593	-0.978710
2	6	0	0.700696	-2.174299	-0.940510
3	6	0	1.512199	-1.368902	0.053890

4	6	0	1.142504	-0.180101	0.599690
5	6	0	-0.153494	0.401704	0.224990
6	6	0	-0.037100	-1.137597	-1.793810
7	8	0	0.076300	-1.091897	-3.003110
8	6	0	-2.018500	-1.126289	-0.340510
9	1	0	-2.665697	-0.497687	0.276790
10	1	0	-2.645401	-1.534187	-1.144510
11	6	0	-0.395107	-3.137895	-0.258010
12	6	0	-1.443504	-2.267291	0.520290
13	1	0	-0.909002	-1.814993	1.364690
14	6	0	-2.610407	-3.085087	1.148890
15	1	0	-2.205910	-3.949988	1.681590
16	1	0	-3.244509	-3.486085	0.347290
17	6	0	-3.477804	-2.268784	2.078690
18	1	0	-4.262802	-1.687381	1.595290
19	6	0	-1.082610	-3.959993	-1.370810
20	1	0	-1.563208	-3.331491	-2.127010
21	1	0	-1.848313	-4.609890	-0.937810
22	1	0	-0.373813	-4.609995	-1.892210
23	6	0	0.309789	-4.104598	0.714390
24	1	0	-0.407314	-4.803695	1.154190
25	1	0	0.804891	-3.570700	1.532890
26	1	0	1.058287	-4.715301	0.199790
27	6	0	-1.549292	0.846009	-1.939910

28	1	0	-2.117495	0.259211	-2.670410
29	1	0	-0.734391	1.285906	-2.521310
30	6	0	-2.452188	1.975412	-1.384910
31	1	0	-2.966189	1.618514	-0.485510
32	6	0	2.449811	1.857494	1.536190
33	6	0	1.971906	0.455396	1.690790
34	6	0	-1.709183	3.260710	-1.007810
35	6	0	-3.544187	2.347717	-2.428610
36	1	0	-3.980383	3.315718	-2.153910
37	1	0	-3.056186	2.511415	-3.398610
38	8	0	-0.646791	1.318906	0.866690
39	6	0	-0.712981	3.778606	-1.734610
40	1	0	-0.313683	3.294804	-2.622210
41	1	0	-0.237178	4.715304	-1.458110
42	8	0	2.264904	-0.209505	2.676990
43	6	0	2.333914	2.566595	0.329490
44	1	0	1.855112	2.132296	-0.540210
45	6	0	2.833219	3.858093	0.222990
46	6	0	3.453621	4.454090	1.335290
47	6	0	3.574419	3.757190	2.536390
48	1	0	4.057320	4.231488	3.388890
49	6	0	3.078214	2.461192	2.635290
50	1	0	3.167012	1.898191	3.557490
51	6	0	-2.242381	3.976212	0.208290

52	1	0	-3.319280	4.178216	0.112690
53	1	0	-1.735977	4.933010	0.371290
54	1	0	-2.106283	3.352511	1.098490
55	6	0	-4.683391	1.322221	-2.615910
56	1	0	-4.274994	0.357319	-2.940310
57	1	0	-5.309789	1.681223	-3.447710
58	6	0	-5.577291	1.104924	-1.410710
59	6	0	-6.261487	2.322527	-0.836410
60	1	0	-6.802085	2.875929	-1.616510
61	1	0	-5.538884	3.023824	-0.399910
62	1	0	-6.975988	2.049529	-0.053810
63	6	0	-5.788596	-0.116075	-0.905010
64	1	0	-5.310399	-0.998677	-1.323310
65	1	0	-6.459997	-0.280872	-0.065310
66	6	0	1.825094	-2.916104	-1.703410
67	1	0	2.049396	-2.359304	-2.616710
68	1	0	1.545890	-3.926003	-2.004610
69	6	0	3.017293	-2.939208	-0.734610
70	1	0	3.091490	-3.885608	-0.191810
71	8	0	2.712497	-1.922707	0.274490
72	6	0	4.425295	-2.627713	-1.288110
73	6	0	4.520400	-1.246814	-1.954310
74	1	0	4.223703	-0.453213	-1.260110
75	1	0	3.895700	-1.167711	-2.850410

76	1	0	5.559300	-1.068918	-2.247510
77	6	0	4.853890	-3.740615	-2.246810
78	1	0	4.798287	-4.717515	-1.754810
79	1	0	5.892091	-3.575819	-2.550510
80	1	0	4.228490	-3.758513	-3.145310
81	8	0	5.338094	-2.694917	-0.187010
82	1	0	5.030397	-2.052416	0.474790
83	8	0	2.723722	4.541093	-0.953310
84	1	0	3.121825	5.418392	-0.821310
85	8	0	3.910426	5.732889	1.128590
86	1	0	4.312427	6.072787	1.942390
87	6	0	-3.379604	-2.173384	3.414290
88	6	0	-4.329000	-1.298580	4.197790
89	1	0	-3.783197	-0.523983	4.754190
90	1	0	-4.883003	-1.885278	4.944390
91	1	0	-5.055999	-0.800578	3.548790
92	6	0	-2.351706	-2.890188	4.255590
93	1	0	-2.839709	-3.505586	5.024190
94	1	0	-1.721304	-2.167190	4.790990
95	1	0	-1.689609	-3.537790	3.675890

Conformer 5b-2

Center	Atomic	Atomic	Coordinates (Angstroms)		
Number	Number	Type	X	Y	Z
1	6	0	-0.848597	-0.267302	-0.875203

2	6	0	0.706209	-2.297097	-0.736803
3	6	0	1.552907	-1.474295	0.210997
4	6	0	1.226603	-0.251496	0.705497
5	6	0	-0.041699	0.368000	0.298597
6	6	0	0.003706	-1.280199	-1.640903
7	8	0	0.107406	-1.309399	-2.851603
8	6	0	-1.966195	-1.109905	-0.183903
9	1	0	-2.592497	-0.430507	0.402897
10	1	0	-2.608393	-1.537507	-0.965503
11	6	0	-0.420188	-3.179001	0.002897
12	6	0	-1.434791	-2.228504	0.732697
13	1	0	-0.878893	-1.756502	1.553697
14	6	0	-2.631889	-2.965807	1.391197
15	1	0	-2.258786	-3.841606	1.939597
16	1	0	-3.301688	-3.352309	0.616297
17	6	0	-3.383592	-2.087710	2.360297
18	1	0	-2.744593	-1.537708	3.054297
19	6	0	-1.143886	-4.037803	-1.058003
20	1	0	-1.609688	-3.435504	-1.844203
21	1	0	-1.926384	-4.637805	-0.584203
22	1	0	-0.461584	-4.738901	-1.547303
23	6	0	0.256114	-4.111299	1.027597
24	1	0	-0.484984	-4.739801	1.529497
25	1	0	0.794913	-3.548497	1.797497

26	1	0	0.961016	-4.792997	0.541297
27	6	0	-1.439200	0.744096	-1.897203
28	1	0	-2.144098	0.157094	-2.498303
29	1	0	-0.645901	1.011299	-2.599803
30	6	0	-2.146404	2.033294	-1.416203
31	1	0	-2.659103	1.837693	-0.468603
32	6	0	2.640797	1.737709	1.602397
33	6	0	2.061401	0.380507	1.796297
34	6	0	-1.212208	3.225297	-1.193903
35	6	0	-3.225205	2.454691	-2.456303
36	1	0	-3.466908	3.514590	-2.313603
37	1	0	-2.786605	2.386392	-3.460903
38	8	0	-0.495902	1.331999	0.899597
39	6	0	-0.168708	3.509200	-1.981503
40	1	0	0.132993	2.880301	-2.815203
41	1	0	0.447089	4.387502	-1.810703
42	8	0	2.270103	-0.258793	2.820197
43	6	0	2.599395	2.407708	0.368797
44	1	0	2.102396	1.978307	-0.493303
45	6	0	3.194891	3.654810	0.228097
46	6	0	3.834489	4.245312	1.332497
47	6	0	3.879091	3.587412	2.560597
48	1	0	4.377890	4.057314	3.406397
49	6	0	3.288295	2.335210	2.693997

50	1	0	3.316997	1.801911	3.637397
51	6	0	-1.602610	4.139596	-0.059003
52	1	0	-2.645311	4.474593	-0.158403
53	1	0	-0.966013	5.029098	-0.016203
54	1	0	-1.524909	3.606296	0.894697
55	6	0	-4.534003	1.633487	-2.422303
56	1	0	-4.306200	0.564588	-2.515703
57	1	0	-5.120404	1.901785	-3.314303
58	6	0	-5.393003	1.856884	-1.193903
59	6	0	-6.000908	3.229483	-1.026603
60	1	0	-6.590908	3.508281	-1.910703
61	1	0	-5.231810	4.003585	-0.908803
62	1	0	-6.655908	3.274681	-0.151103
63	6	0	-5.622201	0.881484	-0.307503
64	1	0	-5.194498	-0.111015	-0.423303
65	1	0	-6.245401	1.040882	0.569597
66	6	0	1.795911	-3.122394	-1.464003
67	1	0	2.036210	-2.624793	-2.406803
68	1	0	1.476414	-4.135495	-1.708803
69	6	0	2.992711	-3.139890	-0.500503
70	1	0	3.034214	-4.058490	0.091397
71	8	0	2.733308	-2.060491	0.455197
72	6	0	4.407311	-2.911086	-1.077003
73	6	0	4.548807	-1.570886	-1.814603

74	1	0	4.284704	-0.732187	-1.161403
75	1	0	3.922507	-1.514988	-2.711303
76	1	0	5.591806	-1.446583	-2.120303
77	6	0	4.787314	-4.087485	-1.978603
78	1	0	4.699917	-5.034585	-1.435503
79	1	0	5.828314	-3.977082	-2.297003
80	1	0	4.154314	-4.129287	-2.871103
81	8	0	5.325411	-2.954983	0.020897
82	1	0	5.048809	-2.265284	0.647797
83	8	0	3.161989	4.301110	-0.973703
84	1	0	3.620187	5.151511	-0.862003
85	8	0	4.387986	5.478914	1.091097
86	1	0	4.794985	5.819915	1.901997
87	6	0	-4.709892	-1.926514	2.489397
88	6	0	-5.272295	-1.013615	3.553797
89	1	0	-5.925493	-1.565617	4.244497
90	1	0	-5.889997	-0.221517	3.107897
91	1	0	-4.482496	-0.535913	4.141397
92	6	0	-5.754090	-2.616917	1.645497
93	1	0	-6.398188	-3.249519	2.272297
94	1	0	-5.331888	-3.250415	0.861497
95	1	0	-6.414892	-1.881919	1.165897

Conformer 7a-1

Center	Atomic	Atomic	Coordinates (Angstroms)		

Number	Number	Type	X	Y	Z
1	6	0	0.872400	0.765896	-1.111001
2	6	0	-0.901099	2.170797	0.133199
3	6	0	-1.407000	0.846297	0.755299
4	6	0	-0.889200	-0.427203	0.248199
5	6	0	0.156500	-0.479404	-0.617201
6	6	0	-3.251001	-3.371503	1.048299
7	1	0	-2.707101	-3.779303	1.896499
8	6	0	-2.718701	-2.246803	0.389199
9	6	0	-1.438400	-1.687803	0.884499
10	6	0	-4.439101	-3.929002	0.618199
11	6	0	-5.127301	-3.383402	-0.486901
12	6	0	-4.601701	-2.273602	-1.145301
13	1	0	-5.144301	-1.867102	-1.992701
14	6	0	-3.405001	-1.708303	-0.707301
15	1	0	-3.002400	-0.843603	-1.223701
16	8	0	-0.820401	-2.217403	1.801699
17	8	0	-5.051801	-5.023302	1.187599
18	1	0	-4.544202	-5.313802	1.960399
19	8	0	-6.293201	-3.937602	-0.904401
20	1	0	-6.491801	-4.684302	-0.312201
21	8	0	-2.265800	0.863797	1.633699
22	8	0	0.633900	-1.692704	-0.958001
23	6	0	-0.192699	1.878696	-1.185901

24	8	0	-0.397999	2.498097	-2.211501
25	6	0	-2.114399	3.116897	-0.062301
26	1	0	-2.506199	3.349397	0.929199
27	1	0	-1.777899	4.048097	-0.520301
28	6	0	-5.466899	2.854598	-2.070801
29	1	0	-4.985999	2.709298	-3.043801
30	1	0	-6.332999	3.514598	-2.213401
31	1	0	-5.843099	1.887198	-1.717601
32	6	0	-4.472999	3.471398	-1.073101
33	6	0	-3.247399	2.545597	-0.931401
34	1	0	-3.602400	1.594997	-0.516401
35	1	0	-2.864699	2.351897	-1.938701
36	6	0	-5.168999	3.736798	0.271699
37	1	0	-5.491499	2.803698	0.747099
38	1	0	-6.060499	4.360898	0.122999
39	1	0	-4.503999	4.262998	0.962199
40	6	0	1.945200	1.247196	-0.063001
41	1	0	2.529201	2.018296	-0.577901
42	1	0	2.638800	0.424996	0.137099
43	6	0	0.230301	2.814096	1.084499
44	6	0	1.432801	1.818696	1.283299
45	1	0	2.241501	2.460696	1.652499
46	6	0	1.287700	0.724296	2.381199
47	6	0	2.625100	0.453196	3.071799

48	1	0	3.400900	0.244295	2.312299
49	6	0	2.630100	-0.770804	4.025899
50	8	0	3.946300	-0.671005	4.631299
51	1	0	3.970400	-1.279505	5.385999
52	6	0	2.529499	-2.095104	3.262799
53	1	0	2.669899	-2.935804	3.954499
54	1	0	3.314699	-2.156805	2.500699
55	1	0	1.554599	-2.220404	2.781399
56	6	0	1.557700	-0.662704	5.114699
57	1	0	1.700400	-1.448104	5.869299
58	1	0	0.554000	-0.791504	4.697499
59	1	0	1.613600	0.311896	5.608099
60	8	0	2.981400	1.629896	3.792899
61	1	0	3.736100	1.360295	4.346099
62	6	0	1.119499	-1.947704	-2.325801
63	6	0	2.143800	-0.855904	-2.725801
64	1	0	0.587400	1.050696	3.152399
65	1	0	0.896600	-0.210604	1.979799
66	1	0	2.285100	-0.957204	-3.811901
67	6	0	1.509800	0.536496	-2.501601
68	1	0	0.734800	0.705496	-3.253201
69	1	0	2.248600	1.321996	-2.683801
70	6	0	1.703999	-3.357604	-2.234201
71	1	0	2.158299	-3.642804	-3.189001

72	1	0	0.909699	-4.073204	-2.000901
73	1	0	2.462099	-3.427204	-1.451601
74	6	0	3.538100	-1.063105	-2.090301
75	1	0	3.451900	-1.124105	-0.999401
76	1	0	3.928499	-2.028605	-2.423201
77	6	0	-0.104901	-1.965004	-3.254401
78	1	0	0.193599	-2.264904	-4.264801
79	1	0	-0.593100	-0.988903	-3.323201
80	1	0	-0.841101	-2.684603	-2.883501
81	6	0	0.798001	4.095696	0.420499
82	1	0	1.549401	4.538296	1.084299
83	1	0	0.018201	4.845096	0.262199
84	1	0	1.273301	3.912996	-0.547701
85	6	0	-0.356799	3.243097	2.445499
86	1	0	-1.001399	2.482197	2.888999
87	1	0	-0.949199	4.157197	2.340599
88	1	0	0.461601	3.456596	3.142699
89	6	0	4.571900	0.000495	-2.490601
90	1	0	4.370000	0.955695	-1.991601
91	1	0	4.494800	0.186295	-3.570901
92	6	0	6.036400	-0.386305	-2.201101
93	6	0	6.975100	0.724994	-2.695801
94	1	0	6.804400	1.665494	-2.158901
95	1	0	6.824800	0.901894	-3.765801

96	1	0	8.024500	0.441694	-2.541301
97	6	0	6.282100	-0.671005	-0.711101
98	1	0	5.678200	-1.515705	-0.367001
99	1	0	6.045600	0.201195	-0.090401
100	1	0	7.337400	-0.920006	-0.538301
101	8	0	6.270500	-1.581805	-2.968901
102	1	0	7.179199	-1.870006	-2.787001
103	8	0	-3.969099	4.708298	-1.610001
104	1	0	-4.722998	5.313798	-1.693001

Conformer 7a-2

Center	Atomic	Atomic	Coordinates (Angstroms)		
Number	Number	Type	X	Y	Z
1	6	0	0.805004	0.888097	-1.066502
2	6	0	-0.942196	2.176097	0.332398
3	6	0	-1.401796	0.804997	0.886698
4	6	0	-0.875396	-0.422803	0.287498
5	6	0	0.136404	-0.401103	-0.619702
6	6	0	-3.174095	-3.463903	0.882598
7	1	0	-2.613095	-3.923003	1.692598
8	6	0	-2.673095	-2.279903	0.307598
9	6	0	-1.400295	-1.733603	0.836598
10	6	0	-4.352795	-4.014804	0.419898
11	6	0	-5.063795	-3.402404	-0.634702
12	6	0	-4.570195	-2.232804	-1.209102

13	1	0	-5.129895	-1.775004	-2.018302
14	6	0	-3.382195	-1.675104	-0.738502
15	1	0	-3.005396	-0.763803	-1.190202
16	8	0	-0.768795	-2.316503	1.710598
17	8	0	-4.934595	-5.163804	0.907898
18	1	0	-4.407595	-5.506704	1.645398
19	8	0	-6.220195	-3.950304	-1.085002
20	1	0	-6.394195	-4.747004	-0.553102
21	8	0	-2.233396	0.752597	1.790198
22	8	0	0.600404	-1.584503	-1.068102
23	6	0	-0.282096	1.978697	-1.032102
24	8	0	-0.537296	2.659497	-2.006102
25	6	0	-2.182597	3.102897	0.233298
26	1	0	-2.548897	3.264597	1.248198
27	1	0	-1.883097	4.067797	-0.178202
28	6	0	-5.585397	2.889696	-1.693802
29	1	0	-5.129997	2.797196	-2.685402
30	1	0	-6.463797	3.542996	-1.781602
31	1	0	-5.938696	1.901596	-1.376602
32	6	0	-4.572197	3.472896	-0.695502
33	6	0	-3.331496	2.558396	-0.632602
34	1	0	-3.660996	1.582796	-0.256802
35	1	0	-2.975396	2.422397	-1.658802
36	6	0	-5.234297	3.665896	0.678398

37	1	0	-5.530396	2.707196	1.118998
38	1	0	-6.138097	4.282596	0.582498
39	1	0	-4.558197	4.170696	1.373998
40	6	0	1.900504	1.362798	-0.047302
41	1	0	2.387404	2.214998	-0.535402
42	1	0	2.672004	0.599298	0.044598
43	6	0	0.208803	2.790597	1.287498
44	6	0	1.453104	1.826998	1.360898
45	1	0	2.283704	2.459598	1.695198
46	6	0	1.389704	0.716698	2.447998
47	6	0	2.612904	-0.221402	2.455398
48	1	0	2.527204	-0.924502	1.618498
49	6	0	2.763004	-1.057802	3.765298
50	8	0	3.301204	-0.167502	4.778698
51	1	0	2.559304	0.339398	5.145598
52	6	0	3.836205	-2.132502	3.571598
53	1	0	4.047205	-2.625302	4.525498
54	1	0	4.759705	-1.688002	3.191398
55	1	0	3.492705	-2.886402	2.855098
56	6	0	1.452005	-1.678202	4.248498
57	1	0	1.642405	-2.325102	5.111798
58	1	0	0.974205	-2.265203	3.458698
59	1	0	0.730404	-0.910503	4.553198
60	8	0	3.830204	0.501898	2.253998

61	1	0	4.103404	0.759798	3.155898
62	6	0	1.043005	-1.734203	-2.467502
63	6	0	2.056704	-0.611902	-2.801002
64	1	0	1.337104	1.227798	3.418298
65	1	0	0.493904	0.098297	2.378198
66	1	0	2.203604	-0.640002	-3.890502
67	6	0	1.406104	0.754998	-2.485902
68	1	0	0.611504	0.954097	-3.209002
69	1	0	2.129904	1.560598	-2.637902
70	6	0	1.629505	-3.145902	-2.504102
71	1	0	2.051805	-3.353802	-3.493002
72	1	0	0.843105	-3.879503	-2.302202
73	1	0	2.413705	-3.275902	-1.755902
74	6	0	3.441904	-0.859402	-2.159502
75	1	0	3.324304	-1.048202	-1.086302
76	1	0	3.859905	-1.774702	-2.588702
77	6	0	-0.209295	-1.683003	-3.356502
78	1	0	0.063305	-1.876603	-4.399802
79	1	0	-0.715296	-0.714703	-3.314602
80	1	0	-0.922695	-2.449003	-3.037702
81	6	0	0.690403	4.143697	0.704098
82	1	0	1.482203	4.549998	1.343898
83	1	0	-0.118297	4.878497	0.677698
84	1	0	1.089703	4.064098	-0.311002

85	6	0	-0.337397	3.083697	2.699798
86	1	0	-0.815896	2.216697	3.158198
87	1	0	-1.069397	3.895997	2.677098
88	1	0	0.486203	3.412397	3.345798
89	6	0	4.472304	0.254398	-2.387802
90	1	0	4.215604	1.150298	-1.811802
91	1	0	4.468404	0.544698	-3.447602
92	6	0	5.920204	-0.139001	-2.030502
93	6	0	6.852104	1.061999	-2.253702
94	1	0	6.615504	1.885999	-1.570502
95	1	0	6.767704	1.425899	-3.283302
96	1	0	7.897504	0.776299	-2.079102
97	6	0	6.050004	-0.663001	-0.591002
98	1	0	5.517204	-1.611701	-0.477302
99	1	0	5.648604	0.040999	0.146498
100	1	0	7.105704	-0.840601	-0.345302
101	8	0	6.264604	-1.184501	-2.961502
102	1	0	7.147704	-1.506101	-2.719602
103	8	0	-4.097997	4.740096	-1.186402
104	1	0	-4.863197	5.335196	-1.233702

Conformer 7b-2

Center	Atomic	Atomic	Coordinates (Angstroms)		
Number	Number	Type	X	Y	Z
1	6	0	0.981390	0.815511	-0.598001

2	6	0	-0.824818	1.993599	0.805699
3	6	0	-1.574509	0.635293	0.858199
4	6	0	-1.048201	-0.502203	0.111299
5	6	0	0.111899	-0.419295	-0.609001
6	6	0	-3.640681	-3.363721	0.045699
7	1	0	-3.138776	-4.044518	0.728299
8	6	0	-3.018589	-2.137617	-0.262301
9	6	0	-1.705391	-1.842908	0.342699
10	6	0	-4.862978	-3.678330	-0.513601
11	6	0	-5.496385	-2.781034	-1.400701
12	6	0	-4.881993	-1.569130	-1.711601
13	1	0	-5.384298	-0.889333	-2.392001
14	6	0	-3.651295	-1.250021	-1.143401
15	1	0	-3.178102	-0.305618	-1.388401
16	8	0	-1.128785	-2.675204	1.048799
17	8	0	-5.563470	-4.842535	-0.283301
18	1	0	-5.087466	-5.384231	0.363999
19	8	0	-6.696582	-3.098943	-1.946601
20	1	0	-6.957176	-3.968844	-1.595601
21	8	0	-2.595508	0.533586	1.536899
22	8	0	0.487706	-1.503892	-1.284801
23	6	0	0.087382	2.042505	-0.416401
24	8	0	0.181475	3.008606	-1.150901
25	6	0	-1.854726	3.149691	0.757699

26	1	0	-2.414326	3.152287	1.694699
27	1	0	-1.301733	4.089995	0.688299
28	6	0	-4.734035	4.390971	0.670799
29	1	0	-5.254228	3.451468	0.883699
30	1	0	-5.489540	5.167966	0.491599
31	1	0	-4.170337	4.685375	1.562799
32	6	0	-3.827134	4.232978	-0.559001
33	6	0	-2.847826	3.051284	-0.413101
34	1	0	-3.457519	2.148580	-0.301701
35	1	0	-2.307425	2.969788	-1.361301
36	6	0	-3.106043	5.549683	-0.884801
37	1	0	-2.483045	5.895787	-0.052901
38	1	0	-3.836849	6.342177	-1.096101
39	1	0	-2.468842	5.426887	-1.766401
40	6	0	1.897290	0.787918	0.678399
41	1	0	2.581384	1.642422	0.601999
42	1	0	2.503697	-0.115578	0.644999
43	6	0	0.163381	2.060106	2.097999
44	6	0	1.119090	0.817512	2.001199
45	1	0	0.473396	-0.060192	2.002999
46	6	0	2.065092	0.607719	3.203099
47	6	0	1.596507	-1.600784	-2.261701
48	6	0	2.579599	-0.410678	-2.130801
49	1	0	2.806686	1.422024	3.245799

50	6	0	2.862101	-0.719276	3.253099
51	1	0	3.595601	-0.739470	2.431599
52	6	0	2.058610	-2.047581	3.129999
53	8	0	1.644211	-2.124484	1.755699
54	1	0	0.710113	-2.409491	1.680499
55	6	0	0.861511	-2.097590	4.086499
56	1	0	0.120205	-1.324395	3.861799
57	1	0	1.198710	-1.962687	5.118899
58	1	0	0.360217	-3.069393	4.012199
59	6	0	2.994119	-3.238675	3.395499
60	1	0	3.847318	-3.206869	2.708399
61	1	0	2.449325	-4.171578	3.212999
62	1	0	3.373819	-3.238272	4.420299
63	8	0	3.555701	-0.777271	4.507299
64	1	0	4.005695	0.072732	4.630499
65	1	0	1.497491	0.663115	4.136799
66	1	0	3.204200	-0.606373	-1.256101
67	6	0	1.815390	0.896517	-1.881801
68	1	0	1.160088	1.135213	-2.729001
69	1	0	2.512184	1.732622	-1.789601
70	6	0	2.258816	-2.929280	-1.885901
71	1	0	3.123218	-3.128874	-2.527801
72	1	0	1.549522	-3.755085	-2.001201
73	1	0	2.581816	-2.900678	-0.840701

74	6	0	3.526098	-0.302271	-3.349101
75	1	0	3.927105	-1.294268	-3.591701
76	1	0	2.945396	0.007125	-4.227901
77	6	0	0.895508	-1.690089	-3.622601
78	1	0	1.602310	-1.977584	-4.406801
79	1	0	0.435901	-0.737593	-3.906201
80	1	0	0.108013	-2.447795	-3.576701
81	6	0	0.944072	3.392711	2.094799
82	1	0	1.677072	3.398116	2.909199
83	1	0	0.273066	4.240506	2.263399
84	1	0	1.479371	3.581515	1.159899
85	6	0	-0.656318	1.998600	3.403199
86	1	0	-1.180611	1.047196	3.521799
87	1	0	-1.404924	2.793895	3.442399
88	1	0	0.002481	2.143504	4.265399
89	6	0	4.700991	0.688437	-3.219001
90	1	0	4.321184	1.701335	-3.040401
91	1	0	5.204991	0.722941	-4.193701
92	6	0	5.785093	0.414645	-2.155701
93	6	0	6.325303	-1.021851	-2.214301
94	1	0	6.736305	-1.255048	-3.203501
95	1	0	5.542308	-1.749857	-1.983501
96	1	0	7.133104	-1.156546	-1.483101
97	6	0	6.932386	1.423453	-2.330501

98	1	0	6.550179	2.448450	-2.280801
99	1	0	7.445887	1.288757	-3.289301
100	1	0	7.678487	1.299958	-1.534601
101	8	0	5.157891	0.645541	-0.876301
102	1	0	5.823193	0.470645	-0.191301
103	8	0	-4.633931	3.849272	-1.691301
104	1	0	-5.279736	4.559567	-1.836101

Conformer 11a-1

Center	Atomic	Atomic	Coordinates (Angstroms)		
Number	Number	Type	X	Y	Z
1	6	0	0.863802	-0.095894	1.059899
2	6	0	2.037305	-1.697592	-0.571801
3	6	0	1.128904	-1.144993	-1.705801
4	6	0	0.082102	-0.139996	-1.380001
5	6	0	0.016601	0.361504	-0.107201
6	6	0	1.409105	-1.499593	0.795799
7	8	0	1.467207	-2.334993	1.680399
8	6	0	2.164600	0.787909	1.082099
9	1	0	2.738301	0.493110	1.969799
10	1	0	1.874798	1.834408	1.227899
11	6	0	3.413204	-0.824789	-0.529301
12	6	0	3.048501	0.664011	-0.173401
13	1	0	2.476500	1.051709	-1.027201
14	6	0	4.271599	1.610713	-0.018101

15	1	0	4.806399	1.381314	0.908899
16	6	0	3.881796	3.066412	-0.071101
17	1	0	3.372995	3.356311	-0.993201
18	1	0	4.977099	1.419814	-0.836601
19	6	0	4.115204	-0.870587	-1.902901
20	1	0	5.099803	-0.396685	-1.836401
21	1	0	3.541703	-0.364088	-2.682801
22	1	0	4.281806	-1.899087	-2.232201
23	6	0	4.379005	-1.427887	0.515399
24	1	0	3.940505	-1.516088	1.513399
25	1	0	5.277903	-0.808085	0.592999
26	1	0	4.708207	-2.425886	0.213399
27	6	0	0.153602	0.019405	2.435999
28	1	0	0.933702	-0.152794	3.184999
29	1	0	-0.176200	1.054204	2.569399
30	6	0	-1.023096	-0.939998	2.783299
31	1	0	-0.652694	-1.959797	2.637799
32	6	0	-1.301797	-0.774198	4.281399
33	6	0	-2.277997	-0.785100	1.899199
34	1	0	-2.698199	0.224299	1.992099
35	1	0	-1.986096	-0.902300	0.849299
36	6	0	-2.028499	0.232200	4.778699
37	1	0	-2.505600	0.974599	4.143499
38	1	0	-2.181199	0.348300	5.848999

39	6	0	-0.655194	-1.802097	5.177299
40	1	0	0.421306	-1.883395	4.975599
41	1	0	-1.076792	-2.799198	4.989699
42	1	0	-0.791895	-1.567097	6.237599
43	6	0	-3.373794	-1.823203	2.218799
44	1	0	-3.660895	-1.728803	3.271199
45	1	0	-2.942592	-2.829202	2.094499
46	6	0	-4.607095	-1.698805	1.346899
47	6	0	-4.449394	-2.028905	-0.118401
48	1	0	-5.403894	-1.967007	-0.650001
49	1	0	-4.049292	-3.043304	-0.252101
50	1	0	-3.745695	-1.349703	-0.617001
51	6	0	-5.783695	-1.320707	1.858399
52	1	0	-5.900296	-1.091608	2.915199
53	1	0	-6.674596	-1.230309	1.241199
54	6	0	2.279108	-3.212591	-0.840001
55	1	0	2.808009	-3.316990	-1.786201
56	1	0	2.928809	-3.594290	-0.046801
57	6	0	-0.831199	0.375903	-2.436601
58	6	0	-1.692001	1.499401	-1.993501
59	6	0	-1.627402	1.963201	-0.682001
60	8	0	-0.782201	1.389603	0.241999
61	6	0	-2.582402	2.120099	-2.886401
62	1	0	-2.635602	1.758199	-3.907101

63	6	0	-3.377005	3.172397	-2.469101
64	6	0	-3.288606	3.621198	-1.128001
65	6	0	-2.418604	3.022799	-0.229501
66	1	0	-2.343205	3.355900	0.801599
67	8	0	-4.238306	3.774696	-3.332601
68	1	0	-4.710507	4.472995	-2.847701
69	8	0	-4.119008	4.666196	-0.820501
70	1	0	-4.032508	4.900096	0.116099
71	8	0	-0.901398	-0.064998	-3.575501
72	8	0	1.330005	-1.514493	-2.848701
73	6	0	1.013910	-4.037994	-0.843101
74	1	0	0.536510	-4.132495	0.130899
75	6	0	0.441811	-4.675495	-1.877301
76	6	0	0.951311	-4.676094	-3.296801
77	1	0	0.182811	-4.282495	-3.975101
78	1	0	1.169313	-5.701993	-3.626601
79	1	0	1.843510	-4.065392	-3.436701
80	6	0	-0.820387	-5.480497	-1.668901
81	1	0	-1.636888	-5.101299	-2.299501
82	1	0	-1.154687	-5.457498	-0.626501
83	1	0	-0.671785	-6.531597	-1.954701
84	6	0	4.092194	4.030813	0.839299
85	6	0	3.641791	5.448912	0.575899
86	1	0	4.493689	6.143313	0.591199

87	1	0	2.946990	5.795210	1.354099
88	1	0	3.143491	5.547211	-0.393601
89	6	0	4.782794	3.834714	2.167299
90	1	0	4.130993	4.153313	2.992599
91	1	0	5.685593	4.458116	2.229099
92	1	0	5.079496	2.800515	2.355399

Conformer 11a-2

Center	Atomic	Atomic	Coordinates (Angstroms)		
Number	Number	Type	X	Y	Z
1	6	0	0.581700	-0.491799	1.339202
2	6	0	2.092304	-1.661393	-0.379398
3	6	0	1.453001	-0.776396	-1.486298
4	6	0	0.365397	0.172200	-1.126798
5	6	0	0.021697	0.318499	0.190602
6	6	0	1.174704	-1.802797	0.822002
7	8	0	1.034808	-2.847697	1.432302
8	6	0	1.842697	0.274606	1.886202
9	1	0	2.204999	-0.281593	2.759802
10	1	0	1.514793	1.255604	2.246702
11	6	0	3.419501	-0.906089	0.191802
12	6	0	2.979996	0.447810	0.862702
13	1	0	2.607394	1.090608	0.055602
14	6	0	4.145793	1.243714	1.521402
15	1	0	4.508195	0.695815	2.400302

16	6	0	3.755988	2.637812	1.957102
17	1	0	3.323188	2.714511	2.955102
18	1	0	4.985493	1.293817	0.823402
19	6	0	4.403100	-0.613585	-0.961598
20	1	0	5.352399	-0.246382	-0.558498
21	1	0	4.015197	0.128513	-1.663698
22	1	0	4.630703	-1.516784	-1.532798
23	6	0	4.139804	-1.826286	1.203402
24	1	0	3.498806	-2.156888	2.026102
25	1	0	5.000902	-1.306183	1.633902
26	1	0	4.525608	-2.721985	0.709402
27	6	0	-0.422700	-0.711102	2.503102
28	1	0	0.166402	-1.132800	3.324102
29	1	0	-0.773103	0.266896	2.847202
30	6	0	-1.650196	-1.648607	2.296002
31	1	0	-1.259293	-2.606905	1.938602
32	6	0	-2.252095	-1.900809	3.682602
33	6	0	-2.673198	-1.158910	1.251202
34	1	0	-3.106201	-0.196112	1.550802
35	1	0	-2.150199	-0.980009	0.305402
36	6	0	-3.102698	-1.054012	4.272602
37	1	0	-3.458102	-0.150913	3.782902
38	1	0	-3.485198	-1.238713	5.273502
39	6	0	-1.779591	-3.156707	4.373202

40	1	0	-0.682991	-3.210603	4.394802
41	1	0	-2.117988	-4.048708	3.828502
42	1	0	-2.149291	-3.223109	5.401402
43	6	0	-3.809994	-2.170514	0.995402
44	1	0	-4.322294	-2.381016	1.939902
45	1	0	-3.358991	-3.117613	0.659202
46	6	0	-4.820896	-1.708518	-0.034598
47	6	0	-4.336697	-1.564616	-1.457898
48	1	0	-5.153798	-1.299519	-2.135698
49	1	0	-3.884893	-2.499715	-1.816398
50	1	0	-3.563299	-0.791014	-1.550798
51	6	0	-6.088797	-1.450323	0.304402
52	1	0	-6.440897	-1.561724	1.327402
53	1	0	-6.822998	-1.122925	-0.428198
54	6	0	2.397509	-3.060292	-0.991198
55	1	0	3.127508	-2.939190	-1.790498
56	1	0	2.853211	-3.675691	-0.209898
57	6	0	-0.280606	1.017798	-2.167798
58	6	0	-1.201909	2.044995	-1.623798
59	6	0	-1.427310	2.135594	-0.252398
60	8	0	-0.822307	1.271596	0.633502
61	6	0	-1.856513	2.949892	-2.477098
62	1	0	-1.684212	2.878793	-3.545298
63	6	0	-2.705716	3.912789	-1.962598

64	6	0	-2.914416	3.979289	-0.562598
65	6	0	-2.281113	3.095691	0.297802
66	1	0	-2.434213	3.135090	1.372402
67	8	0	-3.338519	4.790287	-2.786898
68	1	0	-3.893621	5.370685	-2.238398
69	8	0	-3.773220	4.967786	-0.160698
70	1	0	-3.895720	4.936785	0.800302
71	8	0	-0.099305	0.903399	-3.372198
72	8	0	1.898601	-0.842494	-2.617898
73	6	0	1.171811	-3.784897	-1.496098
74	1	0	0.486812	-4.108499	-0.714098
75	6	0	0.855512	-4.097298	-2.763098
76	6	0	1.674611	-3.751595	-3.981398
77	1	0	1.066309	-3.184497	-4.698398
78	1	0	2.001014	-4.665694	-4.497998
79	1	0	2.551009	-3.143992	-3.754998
80	6	0	-0.412485	-4.863302	-3.062398
81	1	0	-1.072487	-4.286805	-3.725798
82	1	0	-0.970184	-5.105804	-2.151998
83	1	0	-0.192281	-5.804102	-3.586898
84	6	0	3.882484	3.777213	1.257602
85	6	0	3.443580	5.098211	1.843702
86	1	0	4.283077	5.805814	1.893702
87	1	0	3.036680	4.983410	2.853502

88	1	0	2.675378	5.573509	1.217602
89	6	0	4.456284	3.868815	-0.135398
90	1	0	3.717283	4.290212	-0.830598
91	1	0	4.775687	2.904616	-0.537498
92	1	0	5.320482	4.547318	-0.155298

Conformer 11a-3

Center	Atomic	Atomic	Coordinates (Angstroms)		
Number	Number	Type	X	Y	Z
1	6	0	-0.381293	-0.518397	-1.288300
2	6	0	-1.841189	-1.393804	0.632800
3	6	0	-1.192094	-0.326201	1.558000
4	6	0	-0.096498	0.531004	1.031000
5	6	0	0.223203	0.453906	-0.297800
6	6	0	-0.948688	-1.729900	-0.546600
7	8	0	-0.807383	-2.861799	-0.973200
8	6	0	-1.655796	0.169897	-1.899500
9	1	0	-2.036593	-0.493204	-2.685900
10	1	0	-1.333700	1.092299	-2.397700
11	6	0	-3.193492	-0.766209	-0.034000
12	6	0	-2.784598	0.488992	-0.894100
13	1	0	-2.403201	1.240894	-0.190300
14	6	0	-3.924401	1.186487	-1.692900
15	1	0	-3.421404	1.862690	-2.402900
16	6	0	-4.889204	2.022683	-0.890300

17	1	0	-4.424107	2.607785	-0.094800
18	1	0	-4.448397	0.457385	-2.318800
19	6	0	-4.185694	-0.358114	1.072500
20	1	0	-5.104395	0.021282	0.618800
21	1	0	-3.784597	0.416188	1.729800
22	1	0	-4.454790	-1.214915	1.696400
23	6	0	-3.883487	-1.837013	-0.908900
24	1	0	-3.257786	-2.206810	-1.726400
25	1	0	-4.801489	-1.428917	-1.343200
26	1	0	-4.179083	-2.701414	-0.308800
27	6	0	0.576409	-0.916193	-2.444600
28	1	0	-0.045289	-1.446796	-3.173700
29	1	0	0.915505	-0.001391	-2.940100
30	6	0	1.807913	-1.826187	-2.157100
31	1	0	1.422017	-2.741289	-1.697200
32	6	0	2.396014	-2.221285	-3.516500
33	6	0	2.846910	-1.234183	-1.182900
34	1	0	3.275206	-0.307581	-1.584900
35	1	0	2.339709	-0.961185	-0.250600
36	6	0	3.198611	-1.417781	-4.222700
37	1	0	3.518507	-0.445680	-3.855400
38	1	0	3.575112	-1.707680	-5.200700
39	6	0	1.969520	-3.570887	-4.039900
40	1	0	0.875421	-3.666992	-4.044200

41	1	0	2.344024	-4.374785	-3.391500
42	1	0	2.335821	-3.751985	-5.055500
43	6	0	3.988514	-2.216278	-0.846400
44	1	0	4.485016	-2.519176	-1.774100
45	1	0	3.544618	-3.124880	-0.409900
46	6	0	5.015712	-1.653273	0.114800
47	6	0	4.557211	-1.376275	1.527000
48	1	0	5.382909	-1.033572	2.158100
49	1	0	4.128315	-2.277977	1.985300
50	1	0	3.772807	-0.608879	1.559400
51	6	0	6.275111	-1.421768	-0.271600
52	1	0	6.609712	-1.629366	-1.285500
53	1	0	7.019709	-1.020664	0.412200
54	6	0	-2.109484	-2.666505	1.489800
55	1	0	-2.820185	-2.411508	2.274400
56	1	0	-2.574580	-3.416707	0.843600
57	6	0	0.592498	1.513907	1.911600
58	6	0	1.531194	2.411311	1.194900
59	6	0	1.729295	2.274712	-0.176900
60	8	0	1.077699	1.302409	-0.903500
61	6	0	2.234690	3.415415	1.882100
62	1	0	2.083189	3.521614	2.950500
63	6	0	3.105786	4.251318	1.207100
64	6	0	3.286487	4.086319	-0.188700

65	6	0	2.603291	3.102516	-0.886900
66	1	0	2.735392	2.964117	-1.956200
67	8	0	3.786682	5.223921	1.870600
68	1	0	4.348179	5.689424	1.227400
69	8	0	4.171083	4.964223	-0.756500
70	1	0	4.272884	4.775524	-1.701800
71	8	0	0.434298	1.598607	3.121600
72	8	0	-1.627895	-0.184703	2.686400
73	6	0	-0.857781	-3.272399	2.080000
74	1	0	-0.187579	-3.720796	1.348300
75	6	0	-0.501181	-3.344598	3.372600
76	6	0	-1.291583	-2.793501	4.532900
77	1	0	-0.692386	-2.055698	5.082600
78	1	0	-1.539179	-3.594202	5.244400
79	1	0	-2.213385	-2.295205	4.232100
80	6	0	0.790822	-4.022592	3.767000
81	1	0	1.452319	-3.327189	4.302500
82	1	0	1.333124	-4.409889	2.898200
83	1	0	0.604626	-4.861693	4.452400
84	6	0	-6.210205	2.177577	-1.072100
85	6	0	-7.002309	3.119674	-0.196100
86	1	0	-7.808207	2.588270	0.329500
87	1	0	-7.487013	3.903872	-0.794800
88	1	0	-6.372511	3.606776	0.554700

89	6	0	-7.026202	1.473474	-2.129000
90	1	0	-7.462205	2.200372	-2.828700
91	1	0	-7.871400	0.938370	-1.674400
92	1	0	-6.450599	0.754776	-2.717600

Conformer 11b-1

Center	Atomic	Atomic	Coordinates (Angstroms)		
Number	Number	Type	X	Y	Z
1	6	0	0.356099	-0.380097	-1.267707
2	6	0	1.092693	-2.198499	0.390693
3	6	0	0.208396	-1.512796	1.471293
4	6	0	-0.502800	-0.249694	1.140693
5	6	0	-0.466399	0.214706	-0.146407
6	6	0	1.500097	-1.219201	-0.693007
7	8	0	2.614997	-1.193104	-1.183907
8	6	0	-0.533904	-1.443094	-2.005707
9	1	0	0.045095	-1.817096	-2.859907
10	1	0	-1.410702	-0.928991	-2.414207
11	6	0	0.207690	-3.329796	-0.378907
12	6	0	-0.986208	-2.618592	-1.118007
13	1	0	-1.638807	-2.212090	-0.335607
14	6	0	-1.885811	-3.576390	-1.953607
15	1	0	-1.318512	-3.944991	-2.817607
16	6	0	-3.154009	-2.927785	-2.459307
17	1	0	-3.065907	-2.398986	-3.409007

18	1	0	-2.133114	-4.454389	-1.351407
19	6	0	-0.346614	-4.352895	0.635393
20	1	0	-0.818916	-5.186493	0.105593
21	1	0	-1.081412	-3.912692	1.313593
22	1	0	0.451285	-4.780197	1.247793
23	6	0	1.107787	-4.094099	-1.376807
24	1	0	1.607089	-3.441501	-2.098707
25	1	0	0.514685	-4.824997	-1.935407
26	1	0	1.885285	-4.655302	-0.851307
27	6	0	0.884103	0.667301	-2.278507
28	1	0	1.394101	0.089700	-3.055307
29	1	0	0.030404	1.151704	-2.762707
30	6	0	1.889106	1.725698	-1.731207
31	1	0	2.472705	1.253496	-0.936507
32	6	0	1.230110	2.959900	-1.124607
33	6	0	2.896407	2.099095	-2.847807
34	1	0	3.294304	1.167294	-3.266607
35	1	0	2.374909	2.603297	-3.673007
36	6	0	1.387411	3.255300	0.171593
37	1	0	1.979509	2.628698	0.833393
38	1	0	0.934614	4.138201	0.617193
39	6	0	0.436513	3.869103	-2.035007
40	1	0	-0.347489	3.325005	-2.577307
41	1	0	1.076615	4.332901	-2.796807

42	1	0	-0.040784	4.673704	-1.467307
43	6	0	4.070710	2.985491	-2.383507
44	1	0	3.687713	3.929892	-1.979907
45	1	0	4.664811	3.241489	-3.274507
46	6	0	4.987308	2.337988	-1.362607
47	6	0	5.657404	1.045886	-1.768007
48	1	0	6.403103	0.736083	-1.029307
49	1	0	6.160904	1.147884	-2.739507
50	1	0	4.929801	0.230488	-1.868507
51	6	0	5.206110	2.895687	-0.166407
52	1	0	4.716813	3.819189	0.132293
53	1	0	5.883709	2.448085	0.557393
54	6	0	2.354992	-2.776103	1.096393
55	1	0	2.038589	-3.541502	1.803693
56	1	0	2.974790	-3.256605	0.333693
57	6	0	-1.321998	0.452009	2.165193
58	6	0	-2.150394	1.554711	1.620693
59	6	0	-2.080293	1.889211	0.270593
60	8	0	-1.235795	1.226808	-0.591407
61	6	0	-3.024692	2.275714	2.451693
62	1	0	-3.076293	2.017614	3.503693
63	6	0	-3.804289	3.295617	1.936893
64	6	0	-3.713388	3.610416	0.558893
65	6	0	-2.855390	2.914414	-0.278707

66	1	0	-2.773389	3.150413	-1.335607
67	8	0	-4.653086	3.993120	2.740093
68	1	0	-5.099684	4.662321	2.194093
69	8	0	-4.527784	4.634919	0.151793
70	1	0	-4.407884	4.801919	-0.795407
71	8	0	-1.335199	0.176909	3.357193
72	8	0	0.075594	-2.062096	2.550193
73	6	0	3.194795	-1.727606	1.787193
74	1	0	3.697697	-1.037208	1.112293
75	6	0	3.408095	-1.569407	3.103493
76	6	0	2.813293	-2.420605	4.197093
77	1	0	2.213795	-1.799803	4.875793
78	1	0	3.607891	-2.877707	4.804093
79	1	0	2.159490	-3.210203	3.825493
80	6	0	4.308899	-0.460810	3.597693
81	1	0	3.767301	0.210292	4.278993
82	1	0	4.709201	0.140189	2.774793
83	1	0	5.156398	-0.864413	4.169993
84	6	0	-4.362309	-2.931281	-1.873207
85	6	0	-5.537307	-2.234278	-2.517307
86	1	0	-5.951704	-1.461576	-1.854507
87	1	0	-6.354709	-2.941575	-2.716107
88	1	0	-5.262705	-1.758978	-3.464407
89	6	0	-4.676011	-3.604780	-0.559407

90	1	0	-5.032809	-2.869879	0.175193
91	1	0	-3.818813	-4.120583	-0.120507
92	1	0	-5.486014	-4.337178	-0.682007

Conformer 11b-2

Center	Atomic	Atomic	Coordinates (Angstroms)		
Number	Number	Type	X	Y	Z
1	6	0	-0.191299	0.636495	-0.983394
2	6	0	-1.393509	1.851586	0.935406
3	6	0	-0.577502	0.986892	1.938606
4	6	0	0.346706	-0.056601	1.421406
5	6	0	0.551106	-0.152300	0.071506
6	6	0	-1.507003	1.173285	-0.415294
7	8	0	-2.528104	1.180777	-1.080294
8	6	0	0.624891	1.945201	-1.274494
9	1	0	0.109087	2.475197	-2.085594
10	1	0	1.613293	1.667208	-1.657094
11	6	0	-0.580219	3.233292	0.640006
12	6	0	0.786084	2.877602	-0.057394
13	1	0	1.383888	2.345306	0.694706
14	6	0	1.631575	4.112008	-0.479494
15	1	0	1.167971	4.604805	-1.339894
16	6	0	3.067277	3.754919	-0.769994
17	1	0	3.590181	3.296823	0.072506
18	1	0	1.624369	4.845008	0.337106

19	6	0	-0.306924	3.980394	1.962206
20	1	0	0.116568	4.967997	1.753906
21	1	0	0.383380	3.439199	2.613506
22	1	0	-1.229626	4.142787	2.524106
23	6	0	-1.436926	4.154585	-0.256794
24	1	0	-1.754722	3.678883	-1.189094
25	1	0	-0.871532	5.056189	-0.511994
26	1	0	-2.338328	4.483379	0.267506
27	6	0	-0.428594	-0.147907	-2.297094
28	1	0	-0.906799	0.563189	-2.977194
29	1	0	0.540908	-0.396000	-2.740294
30	6	0	-1.339684	-1.407914	-2.203094
31	1	0	-2.084586	-1.222620	-1.424394
32	6	0	-0.601775	-2.686908	-1.819694
33	6	0	-2.126483	-1.574620	-3.527594
34	1	0	-2.570090	-0.604823	-3.781794
35	1	0	-1.436881	-1.815415	-4.347894
36	6	0	-0.894270	-3.335211	-0.685594
37	1	0	-1.655473	-2.974116	0.000906
38	1	0	-0.386563	-4.255307	-0.404794
39	6	0	0.432729	-3.232301	-2.777894
40	1	0	1.196124	-2.484095	-3.026994
41	1	0	-0.021868	-3.542204	-3.727894
42	1	0	0.939936	-4.104097	-2.352994

43	6	0	-3.240175	-2.641428	-3.479294
44	1	0	-2.808368	-3.619125	-3.236594
45	1	0	-3.663474	-2.725331	-4.492194
46	6	0	-4.366377	-2.335237	-2.509894
47	6	0	-5.111487	-1.037442	-2.715994
48	1	0	-5.992187	-0.976749	-2.068894
49	1	0	-5.445388	-0.932645	-3.757794
50	1	0	-4.475593	-0.171037	-2.494294
51	6	0	-4.695271	-3.185639	-1.530794
52	1	0	-4.153164	-4.114735	-1.373894
53	1	0	-5.520973	-2.983745	-0.852094
54	6	0	-2.809410	2.087275	1.541606
55	1	0	-2.703615	2.655976	2.464806
56	1	0	-3.379615	2.697071	0.834406
57	6	0	1.112512	-0.924996	2.355906
58	6	0	2.163618	-1.737288	1.697006
59	6	0	2.325818	-1.693387	0.314506
60	8	0	1.512012	-0.917893	-0.480494
61	6	0	3.019024	-2.556782	2.453006
62	1	0	2.890325	-2.593682	3.529106
63	6	0	4.007630	-3.301174	1.835306
64	6	0	4.150629	-3.232673	0.427806
65	6	0	3.315423	-2.433979	-0.338194
66	1	0	3.413423	-2.377479	-1.418394

67	8	0	4.838936	-4.092768	2.566306
68	1	0	5.454439	-4.528263	1.952406
69	8	0	5.160135	-4.006866	-0.082194
70	1	0	5.196034	-3.918065	-1.046594
71	8	0	0.920613	-0.992397	3.562306
72	8	0	-0.674504	1.225691	3.129106
73	6	0	-3.581701	0.811969	1.784506
74	1	0	-3.881997	0.288667	0.878306
75	6	0	-3.955997	0.279867	2.959106
76	6	0	-3.645501	0.853969	4.318806
77	1	0	-3.111896	0.113373	4.929006
78	1	0	-4.573603	1.096762	4.856106
79	1	0	-3.018808	1.745274	4.278406
80	6	0	-4.759187	-1.000139	2.990806
81	1	0	-4.232682	-1.780136	3.558406
82	1	0	-4.957084	-1.385341	1.985206
83	1	0	-5.724788	-0.849247	3.494606
84	6	0	3.771076	3.938624	-1.898594
85	6	0	5.223679	3.529335	-1.974694
86	1	0	5.866773	4.393340	-2.194194
87	1	0	5.390184	2.806636	-2.786194
88	1	0	5.571682	3.077937	-1.040294
89	6	0	3.233971	4.560720	-3.164994
90	1	0	3.795865	5.470924	-3.416994

91	1	0	2.176869	4.829212	-3.104194
92	1	0	3.358976	3.876221	-4.015594

Conformer 15a-1

Center	Atomic	Atomic	Coordinates (Angstroms)		
Number	Number	Type	X	Y	Z
1	6	0	1.433006	0.480008	-0.454700
2	6	0	-0.458385	1.222599	1.143997
3	6	0	-1.412901	0.515396	0.198292
4	6	0	-1.076350	-0.064015	-0.996847
5	6	0	0.359284	-0.204415	-1.357435
6	6	0	-3.715887	-0.001528	-0.055805
7	6	0	-3.490718	-0.580637	-1.301529
8	6	0	-2.129555	-0.641109	-1.874290
9	6	0	-4.985681	0.070539	0.521076
10	6	0	-6.059425	-0.460458	-0.179875
11	6	0	-5.852743	-1.054737	-1.451293
12	6	0	-4.589873	-1.111464	-2.001209
13	8	0	-1.932199	-1.122228	-2.983866
14	8	0	-7.305525	-0.410229	0.348496
15	6	0	0.778964	1.616019	0.318905
16	8	0	1.219696	2.747767	0.340041
17	6	0	-1.133498	2.508251	1.726042
18	6	0	-2.286759	5.441376	-0.618944
19	6	0	-1.473079	4.683118	0.404128

20	6	0	-1.788174	3.414785	0.710779
21	6	0	-0.337433	5.474842	1.001260
22	6	0	1.923830	-0.561600	0.626373
23	6	0	0.081150	0.230489	2.291954
24	6	0	0.830973	-1.004249	1.654612
25	6	0	-0.074197	-2.192764	1.172909
26	6	0	0.276955	-3.495998	1.849189
27	6	0	1.087573	0.994086	3.192813
28	6	0	-1.053643	-0.240547	3.228715
29	8	0	-2.677955	0.541909	0.669224
30	8	0	-6.998172	-1.538925	-2.036827
31	8	0	0.718586	-0.889245	-2.299272
32	6	0	2.570826	1.006193	-1.377230
33	6	0	4.063291	1.148080	-0.930997
34	6	0	4.710815	-0.214913	-0.412268
35	6	0	3.918236	-1.470177	-0.838036
36	6	0	2.681295	-1.769133	0.040083
37	6	0	4.261490	2.218300	0.161698
38	6	0	4.772267	1.680927	-2.200837
39	6	0	6.208345	-0.357341	-0.701937
40	6	0	6.668236	-0.962913	-2.011477
41	6	0	7.109895	-0.004518	0.223845
42	6	0	0.438955	-4.710077	1.300199
43	6	0	0.305947	-5.022959	-0.170329

44	6	0	0.771928	-5.904884	2.163360
45	1	0	-5.136088	0.530837	1.490673
46	1	0	-4.400477	-1.558163	-2.973893
47	1	0	-7.913300	-0.826054	-0.288543
48	1	0	-0.368558	3.052914	2.277288
49	1	0	-1.892902	2.192976	2.449273
50	1	0	-3.104399	4.837055	-1.024554
51	1	0	-2.720366	6.353170	-0.184335
52	1	0	-1.656099	5.768197	-1.457627
53	1	0	-2.650245	2.987895	0.200098
54	1	0	-0.718831	6.379713	1.495074
55	1	0	0.256638	4.910022	1.719930
56	1	0	0.345319	5.811880	0.210034
57	1	0	2.656266	-0.001510	1.214884
58	1	0	1.408685	-1.413453	2.494157
59	1	0	-1.125537	-1.974186	1.388594
60	1	0	-0.026060	-2.306886	0.090250
61	1	0	0.390722	-3.423515	2.933438
62	1	0	1.518711	0.298271	3.921701
63	1	0	1.911493	1.451923	2.640424
64	1	0	0.594169	1.788648	3.759613
65	1	0	-1.910294	-0.667508	2.705131
66	1	0	-0.666099	-1.002850	3.915447
67	1	0	-1.424285	0.586660	3.841467

68	1	0	-6.787985	-1.923252	-2.901543
69	1	0	2.257282	2.002329	-1.707701
70	1	0	2.556108	0.373717	-2.265844
71	1	0	4.650987	-0.169642	0.682042
72	1	0	3.621045	-1.400739	-1.888966
73	1	0	4.569845	-2.349219	-0.769654
74	1	0	3.003053	-2.367623	0.903284
75	1	0	2.006379	-2.407868	-0.535008
76	1	0	3.841671	3.180115	-0.146423
77	1	0	3.782039	1.944885	1.107677
78	1	0	5.331128	2.354889	0.360466
79	1	0	4.336959	2.644145	-2.491370
80	1	0	4.655528	1.003779	-3.054300
81	1	0	5.840491	1.838804	-2.025145
82	1	0	7.761237	-0.981190	-2.067449
83	1	0	6.313604	-1.995444	-2.119812
84	1	0	6.295491	-0.413736	-2.881722
85	1	0	8.178746	-0.104335	0.048766
86	1	0	6.815864	0.392227	1.192608
87	1	0	0.098919	-4.148151	-0.790614
88	1	0	1.222117	-5.496431	-0.549567
89	1	0	-0.505406	-5.745607	-0.336490
90	1	0	0.854082	-5.636775	3.221543
91	1	0	1.721596	-6.363448	1.853474

92	1	0	0.005544	-6.687293	2.069710

Conformer 15a-9

Center	Atomic	Atomic	Coordinates (Angstroms)		
Number	Number	Type	X	Y	Z
1	6	0	1.573556	0.385560	-0.780731
2	6	0	-0.664996	1.543628	-0.110017
3	6	0	-1.349030	0.245483	-0.508261
4	6	0	-0.718587	-0.879761	-0.972812
5	6	0	0.765322	-0.929353	-0.992401
6	6	0	-3.503648	-0.731740	-0.715228
7	6	0	-2.967958	-1.913530	-1.218777
8	6	0	-1.509824	-2.064854	-1.402381
9	6	0	-4.875717	-0.549030	-0.529205
10	6	0	-5.730760	-1.590993	-0.859330
11	6	0	-5.205847	-2.804074	-1.374328
12	6	0	-3.847682	-2.959739	-1.551954
13	8	0	-1.036236	-3.085450	-1.887058
14	8	0	-7.067249	-1.449816	-0.691313
15	6	0	0.640446	1.576378	-0.959555
16	8	0	0.895559	2.512802	-1.690293
17	6	0	-1.519531	2.780436	-0.570337
18	6	0	-4.345409	4.781543	1.267968
19	6	0	-3.040228	4.486824	0.564755
20	6	0	-2.727940	3.227098	0.212895

21	6	0	-2.194911	5.709996	0.300931
22	6	0	2.037521	0.478823	0.726819
23	6	0	-0.223812	1.548996	1.435103
24	6	0	0.860637	0.442802	1.743458
25	6	0	0.335415	-1.002935	2.065039
26	6	0	0.635804	-1.425281	3.482802
27	6	0	0.392620	2.936607	1.746783
28	6	0	-1.422794	1.376163	2.394070
29	8	0	-2.688807	0.327544	-0.379785
30	8	0	-6.159951	-3.750827	-1.661059
31	8	0	1.360926	-1.982201	-1.151368
32	6	0	2.717182	0.395268	-1.848076
33	6	0	4.126273	1.020026	-1.620908
34	6	0	5.060605	0.200887	-0.652288
35	6	0	4.580073	0.078880	0.818434
36	6	0	3.180688	-0.492340	1.093970
37	6	0	4.789984	1.027366	-3.021901
38	6	0	4.075705	2.497340	-1.169922
39	6	0	5.563904	-1.151386	-1.183145
40	6	0	7.059590	-1.208640	-1.408800
41	6	0	4.816638	-2.242419	-1.397504
42	6	0	1.192399	-2.568700	3.913844
43	6	0	1.648905	-3.700348	3.025182
44	6	0	1.395903	-2.815109	5.390832

45	1	0	-5.271942	0.381861	-0.140697
46	1	0	-3.415961	-3.875197	-1.948180
47	1	0	-7.490371	-2.277515	-0.981357
48	1	0	-1.841224	2.553304	-1.596950
49	1	0	-0.817367	3.604530	-0.684194
50	1	0	-4.171806	5.263667	2.240404
51	1	0	-4.934218	3.874606	1.438764
52	1	0	-4.960568	5.479549	0.682665
53	1	0	-3.456706	2.456625	0.448233
54	1	0	-2.007364	6.259578	1.233704
55	1	0	-2.725509	6.405385	-0.364458
56	1	0	-1.229556	5.487382	-0.158695
57	1	0	2.457770	1.483463	0.820817
58	1	0	1.312047	0.792276	2.681286
59	1	0	0.756906	-1.726130	1.367349
60	1	0	-0.747570	-1.056851	1.914834
61	1	0	0.341751	-0.699060	4.244026
62	1	0	1.218180	3.206298	1.081301
63	1	0	0.774263	2.945739	2.774121
64	1	0	-0.362947	3.722188	1.671091
65	1	0	-1.048696	1.247579	3.417059
66	1	0	-2.063909	2.258997	2.385350
67	1	0	-2.048777	0.511668	2.161738
68	1	0	-5.736698	-4.546779	-2.017489

69	1	0	2.294444	0.900226	-2.723191
70	1	0	2.882480	-0.639861	-2.144657
71	1	0	5.962754	0.826575	-0.585197
72	1	0	5.313840	-0.545604	1.344417
73	1	0	4.642457	1.068485	1.292676
74	1	0	3.049453	-1.460236	0.603768
75	1	0	3.116584	-0.690080	2.171339
76	1	0	4.238297	1.684084	-3.704746
77	1	0	4.817946	0.025670	-3.461468
78	1	0	5.819314	1.402139	-2.963119
79	1	0	3.714257	2.636144	-0.147509
80	1	0	5.079615	2.938400	-1.217273
81	1	0	3.418632	3.073264	-1.827635
82	1	0	7.383969	-2.197676	-1.747875
83	1	0	7.377184	-0.469907	-2.157315
84	1	0	7.607074	-0.969051	-0.485801
85	1	0	3.743367	-2.284412	-1.254984
86	1	0	5.283104	-3.162228	-1.745618
87	1	0	2.705538	-3.936355	3.212340
88	1	0	1.083743	-4.615870	3.249833
89	1	0	1.534935	-3.495312	1.958673
90	1	0	2.456649	-2.989514	5.620331
91	1	0	0.856828	-3.714821	5.719927
92	1	0	1.050913	-1.972879	5.999228

Conformer 15b-1					
Center	Atomic	Atomic	Coordinates (Angstroms)		
Number	Number	Type	X	Y	Z
1	6	0	-1.541066	-0.696843	-0.367949
2	6	0	0.473699	-1.223198	1.172100
3	6	0	1.338073	-0.501888	0.155465
4	6	0	0.901579	0.013301	-1.036223
5	6	0	-0.555745	0.065897	-1.311860
6	6	0	3.576309	0.175421	-0.226060
7	6	0	3.253058	0.682765	-1.481989
8	6	0	1.869008	0.609224	-1.995091
9	6	0	4.868997	0.242906	0.298897
10	6	0	5.863322	0.835158	-0.466937
11	6	0	5.556539	1.352774	-1.751282
12	6	0	4.273101	1.275708	-2.248456
13	8	0	1.588391	1.004502	-3.120391
14	8	0	7.128040	0.919214	0.011545
15	6	0	-0.770456	-1.744335	0.420925
16	8	0	-1.128897	-2.902073	0.524426
17	6	0	1.255874	-2.432605	1.777227
18	6	0	4.257553	-3.367348	1.598591
19	6	0	3.138720	-3.780253	0.673688
20	6	0	1.865764	-3.362059	0.755919
21	6	0	3.553304	-4.759901	-0.399717

22	6	0	-2.086880	0.297188	0.736105
23	6	0	-0.076064	-0.210945	2.298491
24	6	0	-0.983837	0.907092	1.653515
25	6	0	-0.250836	2.152857	1.036465
26	6	0	-0.538513	3.423991	1.797125
27	6	0	-0.934205	-1.010299	3.314836
28	6	0	1.068562	0.426232	3.115599
29	8	0	2.620752	-0.425978	0.562270
30	8	0	6.631058	1.909509	-2.403497
31	8	0	-1.004374	0.726348	-2.233614
32	6	0	-2.629104	-1.350091	-1.278654
33	6	0	-4.138730	-1.519228	-0.899566
34	6	0	-4.869800	-0.123202	-0.998492
35	6	0	-4.518141	0.900886	0.105902
36	6	0	-3.053940	1.360001	0.173320
37	6	0	-4.340537	-2.268058	0.436286
38	6	0	-4.700159	-2.433389	-2.017001
39	6	0	-6.392637	-0.176013	-1.160407
40	6	0	-7.266722	-0.591836	0.002977
41	6	0	-6.955016	0.211949	-2.311697
42	6	0	-0.960562	4.607249	1.322923
43	6	0	-1.178175	5.775110	2.256874
44	6	0	-1.244283	4.909691	-0.128569
45	1	0	5.097366	-0.157186	1.279570

46	1	0	4.006875	1.662505	-3.228803
47	1	0	7.670429	1.361750	-0.665298
48	1	0	2.013976	-2.060755	2.466410
49	1	0	0.531932	-3.007225	2.362467
50	1	0	3.955097	-2.633059	2.348736
51	1	0	5.093047	-2.941112	1.026604
52	1	0	4.660544	-4.241187	2.129497
53	1	0	1.158575	-3.767975	0.035604
54	1	0	4.346147	-4.338811	-1.034049
55	1	0	2.714453	-5.038858	-1.044724
56	1	0	3.964314	-5.679512	0.040431
57	1	0	-2.682442	-0.336484	1.398638
58	1	0	-1.546115	1.306527	2.507740
59	1	0	0.832890	1.996753	1.031896
60	1	0	-0.524784	2.273433	-0.012135
61	1	0	-0.362776	3.355437	2.873162
62	1	0	-1.404398	-0.314622	4.019079
63	1	0	-1.725593	-1.604460	2.850805
64	1	0	-0.316263	-1.694932	3.902479
65	1	0	1.803084	0.956527	2.508065
66	1	0	1.606796	-0.326969	3.699222
67	1	0	0.644733	1.143008	3.828990
68	1	0	6.352298	2.243084	-3.269903
69	1	0	-2.252675	-2.353022	-1.505051

70	1	0	-2.615579	-0.791649	-2.216982
71	1	0	-4.499709	0.310235	-1.937667
72	1	0	-5.138445	1.788615	-0.074525
73	1	0	-4.818483	0.524588	1.093321
74	1	0	-2.723410	1.699173	-0.814236
75	1	0	-3.012356	2.236539	0.831627
76	1	0	-4.179504	-1.642294	1.318965
77	1	0	-3.642658	-3.108200	0.499050
78	1	0	-5.354442	-2.672890	0.513346
79	1	0	-4.195870	-3.406805	-2.004250
80	1	0	-5.772903	-2.606409	-1.891836
81	1	0	-4.551860	-1.990278	-3.009138
82	1	0	-7.013939	-0.046551	0.920256
83	1	0	-7.162924	-1.658067	0.235494
84	1	0	-8.322139	-0.404430	-0.218656
85	1	0	-8.034696	0.229484	-2.444041
86	1	0	-6.359335	0.524142	-3.166149
87	1	0	-0.957880	5.515436	3.297305
88	1	0	-0.543585	6.628197	1.978041
89	1	0	-2.216267	6.133120	2.207479
90	1	0	-1.125552	4.047850	-0.788641
91	1	0	-2.268128	5.289333	-0.250734
92	1	0	-0.575927	5.702380	-0.493029

Conformer 15b-2

Center	Atomic	Atomic	Coordinates (Angstroms)		
Number	Number	Type	X	Y	Z
1	6	0	1.774274	-0.538904	0.312999
2	6	0	-0.217547	-0.879599	-1.304632
3	6	0	-1.122562	-0.521974	-0.141486
4	6	0	-0.706904	-0.258976	1.137281
5	6	0	0.744813	-0.106823	1.408199
6	6	0	-3.407471	-0.216726	0.403405
7	6	0	-3.101688	0.006400	1.743907
8	6	0	-1.701679	-0.027371	2.217144
9	6	0	-4.716710	-0.179757	-0.082422
10	6	0	-5.745664	0.085041	0.810237
11	6	0	-5.456560	0.308871	2.180716
12	6	0	-4.156455	0.269037	2.637370
13	8	0	-1.429437	0.112565	3.403492
14	8	0	-7.027487	0.128877	0.373560
15	6	0	1.084118	-1.444645	-0.694423
16	8	0	1.537846	-2.514253	-1.053880
17	6	0	-0.889498	-1.978827	-2.189540
18	6	0	-3.774356	-3.225771	-2.330532
19	6	0	-2.625427	-3.732768	-1.493340
20	6	0	-1.404233	-3.178832	-1.432071
21	6	0	-2.940436	-4.979714	-0.700024
22	6	0	2.205489	0.721000	-0.534352

23	6	0	0.197253	0.427215	-2.155999
24	6	0	1.034672	1.434920	-1.277804
25	6	0	0.217400	2.447235	-0.401974
26	6	0	0.132817	3.819395	-1.025759
27	6	0	-1.034192	1.137198	-2.757348
28	6	0	1.072059	-0.022334	-3.355944
29	8	0	-2.417683	-0.493743	-0.512373
30	8	0	-6.565210	0.556790	2.954847
31	8	0	1.149465	0.378849	2.450356
32	6	0	2.936671	-1.272740	1.050143
33	6	0	4.435514	-1.260647	0.602558
34	6	0	5.100177	0.125188	0.961523
35	6	0	4.608943	1.350355	0.157367
36	6	0	3.114105	1.692400	0.247670
37	6	0	5.093995	-2.350912	1.486572
38	6	0	4.653440	-1.695045	-0.860306
39	6	0	6.635282	0.107342	0.958759
40	6	0	7.295819	0.138419	2.317617
41	6	0	7.374642	0.127099	-0.156986
42	6	0	-0.933255	4.625326	-1.151437
43	6	0	-0.796990	5.987946	-1.790195
44	6	0	-2.329410	4.296630	-0.681619
45	1	0	-4.930378	-0.357647	-1.129601
46	1	0	-3.903182	0.435711	3.681233

47	1	0	-7.594104	0.322500	1.141417
48	1	0	-1.681311	-1.524587	-2.785012
49	1	0	-0.118007	-2.329448	-2.881348
50	1	0	-4.660273	-3.051712	-1.704579
51	1	0	-3.550449	-2.297721	-2.861569
52	1	0	-4.067375	-3.976120	-3.078288
53	1	0	-0.662173	-3.669993	-0.806119
54	1	0	-2.080687	-5.319479	-0.114465
55	1	0	-3.778142	-4.808181	-0.009306
56	1	0	-3.247653	-5.802355	-1.361315
57	1	0	2.825966	0.308964	-1.334803
58	1	0	1.549322	2.060925	-2.019001
59	1	0	-0.780490	2.073221	-0.178367
60	1	0	0.708995	2.546030	0.573798
61	1	0	1.086318	4.201176	-1.398220
62	1	0	-0.707037	2.038628	-3.286404
63	1	0	-1.546280	0.497511	-3.482486
64	1	0	-1.767697	1.444507	-2.010618
65	1	0	1.928576	-0.637524	-3.066951
66	1	0	1.452453	0.861924	-3.879470
67	1	0	0.487149	-0.598086	-4.078618
68	1	0	-6.298154	0.693663	3.876585
69	1	0	2.632962	-2.324113	1.095375
70	1	0	2.926320	-0.900815	2.077496

71	1	0	4.800133	0.309547	2.003594
72	1	0	4.889123	1.243584	-0.899081
73	1	0	5.175478	2.216861	0.521730
74	1	0	2.802135	1.756516	1.295758
75	1	0	2.985523	2.697855	-0.174556
76	1	0	6.167794	-2.424047	1.289435
77	1	0	4.649348	-3.330786	1.277894
78	1	0	4.952400	-2.143360	2.554540
79	1	0	4.091026	-2.610541	-1.064063
80	1	0	5.713846	-1.895121	-1.043925
81	1	0	4.333568	-0.947396	-1.592496
82	1	0	6.994473	-0.718936	2.932465
83	1	0	6.995778	1.038122	2.873357
84	1	0	8.387966	0.136697	2.240034
85	1	0	8.461302	0.136554	-0.112726
86	1	0	6.938231	0.142526	-1.151031
87	1	0	0.229210	6.187264	-2.114685
88	1	0	-1.092595	6.784033	-1.092285
89	1	0	-1.455667	6.083431	-2.665209
90	1	0	-2.661044	5.022359	0.074046
91	1	0	-3.046214	4.365816	-1.511843
92	1	0	-2.419132	3.301439	-0.239983

Conformer 15c-1

Center	Atomic	Atomic	Coordinates (Angstroms)		

Number	Number	Type	X	Y	Z
1	6	0	1.408505	0.131188	-0.681235
2	6	0	-0.740429	1.492464	-0.096229
3	6	0	-1.513706	0.214324	-0.374838
4	6	0	-0.961356	-0.991011	-0.715921
5	6	0	0.516622	-1.135860	-0.706376
6	6	0	-3.728411	-0.627530	-0.526938
7	6	0	-3.271403	-1.888773	-0.899658
8	6	0	-1.825588	-2.159061	-1.031487
9	6	0	-5.086992	-0.333042	-0.391291
10	6	0	-6.008981	-1.340458	-0.637854
11	6	0	-5.564384	-2.632450	-1.019299
12	6	0	-4.218231	-2.899244	-1.148586
13	8	0	-1.415558	-3.257657	-1.388468
14	8	0	-7.334455	-1.090211	-0.515610
15	6	0	0.565811	1.380400	-0.932304
16	8	0	0.914711	2.278716	-1.673249
17	6	0	-1.521005	2.735987	-0.665253
18	6	0	-4.245741	5.034834	0.967949
19	6	0	-2.948594	4.612583	0.316867
20	6	0	-2.712517	3.314458	0.056448
21	6	0	-2.019436	5.759124	-0.001876
22	6	0	1.955146	0.316189	0.786453
23	6	0	-0.287494	1.611521	1.444058

24	6	0	0.822413	0.548348	1.843374
25	6	0	0.323066	-0.805701	2.445744
26	6	0	1.416863	-1.512994	3.207829
27	6	0	-1.500284	1.499058	2.392111
28	6	0	0.305395	3.028232	1.654707
29	8	0	-2.846000	0.400627	-0.278511
30	8	0	-6.579216	-3.534003	-1.234077
31	8	0	1.046597	-2.232285	-0.708737
32	6	0	2.489714	-0.008362	-1.808437
33	6	0	3.954181	-0.530725	-1.621848
34	6	0	4.838966	0.565016	-0.912246
35	6	0	4.493949	0.834007	0.567516
36	6	0	3.084978	1.374339	0.850567
37	6	0	4.054413	-1.912615	-0.950474
38	6	0	4.472581	-0.703139	-3.072815
39	6	0	6.352026	0.352060	-1.056142
40	6	0	7.055752	1.261447	-2.037277
41	6	0	7.049957	-0.526183	-0.325677
42	6	0	1.787284	-2.802887	3.152950
43	6	0	2.879893	-3.320922	4.059981
44	6	0	1.175700	-3.850628	2.254637
45	1	0	-5.422461	0.656787	-0.104649
46	1	0	-3.846352	-3.877591	-1.441801
47	1	0	-7.811208	-1.911366	-0.731586

48	1	0	-0.772114	3.506613	-0.836819
49	1	0	-1.845378	2.440818	-1.673821
50	1	0	-4.802411	5.731592	0.325422
51	1	0	-4.895174	4.179890	1.181255
52	1	0	-4.060427	5.566185	1.912200
53	1	0	-3.497615	2.611693	0.320353
54	1	0	-1.801853	6.344081	0.902490
55	1	0	-2.496319	6.451872	-0.709255
56	1	0	-1.067928	5.446987	-0.437379
57	1	0	2.405115	-0.644003	1.054976
58	1	0	1.323936	1.025019	2.695937
59	1	0	-0.481492	-0.588247	3.157501
60	1	0	-0.112012	-1.465065	1.695046
61	1	0	1.941940	-0.876452	3.924919
62	1	0	-1.156542	1.544280	3.432602
63	1	0	-2.061366	0.569380	2.267613
64	1	0	-2.188901	2.331494	2.238092
65	1	0	-0.462151	3.794806	1.528155
66	1	0	0.689163	3.114628	2.677296
67	1	0	1.126286	3.255624	0.970871
68	1	0	-6.208217	-4.390901	-1.494478
69	1	0	2.574889	0.982540	-2.263210
70	1	0	2.034396	-0.643493	-2.576727
71	1	0	4.613126	1.498572	-1.450028

72	1	0	5.215471	1.574359	0.935955
73	1	0	4.663646	-0.067153	1.170213
74	1	0	2.881964	2.215551	0.175566
75	1	0	3.097578	1.796834	1.862486
76	1	0	3.416148	-2.632183	-1.468255
77	1	0	3.738126	-1.911411	0.094987
78	1	0	5.086832	-2.274469	-0.993417
79	1	0	3.870916	-1.447349	-3.607074
80	1	0	4.417853	0.236204	-3.637412
81	1	0	5.510675	-1.049040	-3.080056
82	1	0	6.924548	2.314792	-1.751225
83	1	0	8.130049	1.055288	-2.084077
84	1	0	6.643640	1.162005	-3.049022
85	1	0	8.126503	-0.627279	-0.443383
86	1	0	6.589071	-1.170321	0.416775
87	1	0	3.285696	-2.536204	4.706690
88	1	0	2.507657	-4.131604	4.702473
89	1	0	3.708194	-3.745929	3.476175
90	1	0	0.510977	-3.447587	1.489442
91	1	0	1.961401	-4.412253	1.733005
92	1	0	0.613517	-4.583444	2.851589

Conformer 15c-2

Center	Atomic	Atomic	Coordinates (Angstroms)		
Number	Number	Type	X	Y	Z

1	6	0	1.377997	-0.052923	-0.789122
2	6	0	-0.735533	1.385121	-0.267080
3	6	0	-1.533536	0.101582	-0.446027
4	6	0	-1.005956	-1.125816	-0.740413
5	6	0	0.467645	-1.300371	-0.674570
6	6	0	-3.754131	-0.731962	-0.492789
7	6	0	-3.327651	-2.012736	-0.836044
8	6	0	-1.885929	-2.289988	-1.023005
9	6	0	-5.106374	-0.427510	-0.310253
10	6	0	-6.036119	-1.442311	-0.477309
11	6	0	-5.632294	-2.754848	-0.826781
12	6	0	-4.287727	-3.025057	-1.005123
13	8	0	-1.485897	-3.381965	-1.403730
14	8	0	-7.388376	-1.282812	-0.327833
15	6	0	0.547011	1.184411	-1.124471
16	8	0	0.888861	2.012821	-1.945771
17	6	0	-1.509894	2.592481	-0.915886
18	6	0	-1.972840	5.664673	-0.502526
19	6	0	-2.899996	4.557953	-0.061378
20	6	0	-2.679175	3.240593	-0.217423
21	6	0	-4.175768	5.045484	0.586263
22	6	0	1.963353	0.249666	0.639319
23	6	0	-0.239322	1.623087	1.246502
24	6	0	0.856113	0.568930	1.701502

25	6	0	0.291198	-0.715567	2.404542
26	6	0	1.347841	-1.696054	2.858113
27	6	0	-1.427389	1.611092	2.232243
28	6	0	0.380938	3.041255	1.325409
29	8	0	-2.862811	0.305888	-0.327177
30	8	0	-6.563394	-3.733340	-0.986389
31	8	0	0.970023	-2.396213	-0.504319
32	6	0	2.421271	-0.308283	-1.931384
33	6	0	3.885691	-0.825792	-1.737812
34	6	0	4.797382	0.315594	-1.144042
35	6	0	4.493892	0.718724	0.314896
36	6	0	3.099245	1.300804	0.587560
37	6	0	3.986204	-2.149708	-0.958021
38	6	0	4.366037	-1.122875	-3.181632
39	6	0	6.304178	0.069675	-1.302873
40	6	0	6.997446	0.890010	-2.366459
41	6	0	7.006204	-0.759349	-0.520739
42	6	0	1.963235	-1.737311	4.051349
43	6	0	1.709309	-0.769846	5.182235
44	6	0	2.990571	-2.802010	4.354882
45	1	0	-5.407017	0.582701	-0.047712
46	1	0	-3.955369	-4.020482	-1.277997
47	1	0	-7.595345	-0.363922	-0.099235
48	1	0	-0.754742	3.337174	-1.159946

49	1	0	-1.857833	2.226047	-1.892685
50	1	0	-2.463073	6.295755	-1.256949
51	1	0	-1.034928	5.306575	-0.931711
52	1	0	-1.728998	6.325695	0.340672
53	1	0	-3.460810	2.568887	0.126316
54	1	0	-3.961502	5.654105	1.476119
55	1	0	-4.823340	4.217014	0.891525
56	1	0	-4.746792	5.689142	-0.097819
57	1	0	2.425830	-0.682426	0.969896
58	1	0	1.391006	1.076469	2.514511
59	1	0	-0.287676	-0.385156	3.270240
60	1	0	-0.413836	-1.249889	1.763433
61	1	0	1.617660	-2.457290	2.127191
62	1	0	-2.114981	2.430645	2.017208
63	1	0	-1.994349	0.677025	2.212590
64	1	0	-1.055812	1.754657	3.254068
65	1	0	-0.381184	3.808280	1.169587
66	1	0	1.177855	3.204304	0.596780
67	1	0	0.804779	3.198785	2.323356
68	1	0	-7.439315	-3.343852	-0.822994
69	1	0	1.938677	-1.007914	-2.623983
70	1	0	2.498761	0.635214	-2.478905
71	1	0	4.569910	1.201145	-1.757073
72	1	0	4.669873	-0.128522	0.989939

73	1	0	5.234109	1.478032	0.597736
74	1	0	3.142629	1.804599	1.560666
75	1	0	2.887375	2.085714	-0.149452
76	1	0	3.310584	-2.893843	-1.386772
77	1	0	3.716800	-2.055556	0.096513
78	1	0	5.007212	-2.540882	-1.012839
79	1	0	5.401623	-1.475984	-3.185846
80	1	0	3.747067	-1.904472	-3.636932
81	1	0	4.302441	-0.233583	-3.821390
82	1	0	6.883868	1.964144	-2.161581
83	1	0	8.068217	0.667407	-2.418386
84	1	0	6.563143	0.716915	-3.358877
85	1	0	6.553326	-1.336394	0.279641
86	1	0	8.078382	-0.885632	-0.652942
87	1	0	1.401277	-1.305803	6.090941
88	1	0	0.938681	-0.029502	4.952456
89	1	0	2.628423	-0.226174	5.442338
90	1	0	3.141222	-3.475593	3.505823
91	1	0	3.961512	-2.355638	4.613180
92	1	0	2.688670	-3.407868	5.221148

Conformer 15d-1

Center	Atomic	Atomic	Coordinates (Angstroms)		
Number	Number	Type	X	Y	Z
1	6	0	-1.406397	-0.545502	-0.382901

2	6	0	0.564704	-0.880696	1.243899
3	6	0	1.461002	-0.275794	0.175799
4	6	0	1.037701	0.129705	-1.035101
5	6	0	-0.422399	0.261901	-1.263301
6	6	0	3.682101	0.320613	-0.251801
7	6	0	3.388899	0.703712	-1.537901
8	6	0	2.011400	0.586808	-2.053301
9	6	0	4.964700	0.429817	0.275799
10	6	0	5.963199	0.931120	-0.520501
11	6	0	5.684997	1.322919	-1.844701
12	6	0	4.421598	1.208715	-2.341401
13	8	0	1.742399	0.821807	-3.195101
14	8	0	7.204398	1.046124	-0.033301
15	6	0	-0.640194	-1.490800	0.515299
16	8	0	-0.980690	-2.615901	0.710499
17	6	0	1.338508	-2.013694	1.981599
18	6	0	4.308311	-3.043285	2.058499
19	6	0	3.225412	-3.494188	1.108599
20	6	0	1.980911	-3.039292	1.074999
21	6	0	3.669516	-4.579387	0.155399
22	6	0	-2.089000	0.477295	0.592599
23	6	0	-0.002699	0.239202	2.236399
24	6	0	-1.062502	1.193899	1.539299
25	6	0	-0.482506	2.511100	0.935099

26	6	0	-1.535009	3.443097	0.375599
27	6	0	1.144698	1.067005	2.848499
28	6	0	-0.686797	-0.464500	3.433799
29	8	0	2.726502	-0.190190	0.567499
30	8	0	6.756696	1.801423	-2.533201
31	8	0	-0.853702	1.027399	-2.068501
32	6	0	-2.309394	-1.305105	-1.412101
33	6	0	-3.785493	-1.772710	-1.213201
34	6	0	-4.655697	-0.539112	-0.805901
35	6	0	-4.607298	-0.172212	0.689899
36	6	0	-3.240898	-0.172108	1.384999
37	6	0	-3.927289	-2.994210	-0.288201
38	6	0	-4.194192	-2.240711	-2.628601
39	6	0	-6.118897	-0.551917	-1.259401
40	6	0	-7.090394	-1.575120	-0.709301
41	6	0	-6.550500	0.371882	-2.102801
42	6	0	-2.186712	4.402495	1.017999
43	6	0	-2.013513	4.742696	2.478999
44	6	0	-3.196815	5.267592	0.302099
45	1	0	5.174801	0.128718	1.282999
46	1	0	4.184497	1.500215	-3.348301
47	1	0	7.779497	1.405426	-0.699001
48	1	0	2.069606	-1.572892	2.638699
49	1	0	0.620409	-2.533796	2.598899

50	1	0	4.609513	-3.867384	2.701199
51	1	0	4.012608	-2.220086	2.693299
52	1	0	5.192510	-2.738782	1.504599
53	1	0	1.312912	-3.485194	0.360099
54	1	0	2.867917	-4.891989	-0.502601
55	1	0	4.019318	-5.453186	0.700599
56	1	0	4.499615	-4.238184	-0.458901
57	1	0	-2.521402	1.245994	-0.030101
58	1	0	-1.664203	1.560797	2.361499
59	1	0	0.241094	2.325403	0.154899
60	1	0	0.055792	3.023702	1.718399
61	1	0	-1.752809	3.307396	-0.670301
62	1	0	0.735796	1.784004	3.553399
63	1	0	1.828300	0.434307	3.400099
64	1	0	1.722597	1.615207	2.119099
65	1	0	-1.410495	-1.211602	3.141399
66	1	0	0.043404	-0.950298	4.068099
67	1	0	-1.197399	0.272098	4.043799
68	1	0	6.516095	2.049522	-3.415501
69	1	0	-1.737992	-2.177203	-1.713301
70	1	0	-2.348396	-0.655805	-2.272401
71	1	0	-4.216699	0.294489	-1.343701
72	1	0	-5.043401	0.819086	0.781999
73	1	0	-5.251196	-0.834614	1.258799

74	1	0	-3.372100	0.367192	2.316799
75	1	0	-2.979095	-1.179807	1.668299
76	1	0	-3.286887	-3.795608	-0.639301
77	1	0	-3.652190	-2.803909	0.736399
78	1	0	-4.945788	-3.364513	-0.294101
79	1	0	-3.501689	-2.994909	-2.990201
80	1	0	-5.184290	-2.679414	-2.636801
81	1	0	-4.194094	-1.420211	-3.337601
82	1	0	-7.072793	-1.616720	0.374299
83	1	0	-8.102494	-1.335423	-1.013201
84	1	0	-6.869791	-2.574319	-1.068301
85	1	0	-7.579500	0.419279	-2.415101
86	1	0	-5.894302	1.116584	-2.518601
87	1	0	-1.707216	5.779897	2.593599
88	1	0	-2.958313	4.635393	3.006799
89	1	0	-1.282611	4.122798	2.980599
90	1	0	-3.292414	4.994792	-0.741801
91	1	0	-2.912418	6.316293	0.353199
92	1	0	-4.177514	5.184589	0.765599

3. Experimental and Computed NMR Chemical Shifts

3.1 ^{13}C- and ^{1}H-NMR chemical shifts

The TMS-corrected computed ^{13}C- and ^{1}H-NMR chemical shifts of compounds **3**,**7** and **15** were fitted to the experimental values by Ordinary Least Squares (OLS) Linear Regression method in order to remove systematic error that results from the conformational search and random error from experimental conditions (**Tables S3-S5**).

Table S3. Experimental and computed chemical shifts of **3**, **3a** and **3b**.

Position	Exp.**3**	**3a**	Residue	**3b**	Residue
1	51.4	49.93325049	1.466749506	51.7544542	-0.354454204
2	170.9	172.787458	-1.887458042	172.2314711	-1.331471132
3	126.3	126.4860548	-0.186054751	124.4001061	1.89989385
4	194.3	194.1673167	0.132683309	194.0809346	0.219065435
5	69	71.09751873	-2.097518735	71.51798118	-2.51798118
6	46.7	50.87619094	-4.176190938	51.37724583	-4.677245832
7	47	46.80988427	0.190115732	47.50987066	-0.509870655
8	39.9	39.22019688	0.679803118	37.70942869	2.190571314
9	206.9	211.6255352	-4.725535213	211.8345758	-4.934575768
10	192.3	191.3436401	0.956359872	192.1271509	0.172849106
11	131.3	126.6103518	4.689648173	127.2813062	4.018693842
12	115.9	110.5544809	5.345519073	111.1279699	4.772030078
13	146	141.6284721	4.371527924	142.1892954	3.810704557
14	151.3	149.1082658	2.191734233	149.6409181	1.659081945
15	115.6	111.7749119	3.825088084	112.4292199	3.170780079
16	123.6	125.8072753	-2.207275316	126.2187064	-2.618706422
17	26.4	26.70479885	-0.304798848	27.44720217	-1.047202172
18	121.6	120.8038464	0.796153581	121.424207	0.175793023
19	134.2	138.6536804	-4.453680394	139.1498829	-4.949882943
20	26.4	26.06136698	0.338633023	26.87487991	-0.474879906
21	18.3	17.27081654	1.029183458	18.08241797	0.217582032
22	27	25.21802956	1.781970441	25.92564594	1.074354056
23	22.8	20.84068558	1.95931442	21.59354946	1.206450538
24	29.8	29.72551807	0.074481935	30.41061499	-0.610614989
25	126.4	125.6046813	0.795318731	126.3611954	0.038804577
26	133.3	137.8617195	-4.561719471	138.0185018	-4.718501826
27	26.2	25.9723377	0.227662299	26.72321698	-0.523216983
28	18.6	17.57259121	1.027408786	18.52689599	0.073104005
29	29.3	33.6370252	-4.3370252	29.59559208	-0.295592083
30	38.9	37.04921206	1.850787939	37.49113365	1.408866352
31	86	84.43637523	1.563624773	85.00051204	0.99948796
32	22.2	27.12074176	-4.920741763	20.66952102	1.530478985
33	29	25.04719791	3.952802094	27.00813488	1.991865118
34	39.7	45.25666173	-5.556661733	41.88798025	-2.187980254

35	199.8	199.4692444	0.330755569	196.9963136	2.803686379
36	145.5	142.300516	3.199484004	141.4766371	4.023362904
37	125.5	127.2756554	-1.775655367	128.402119	-2.902119019
38	17.9	19.48649431	-1.586494305	20.70321077	-2.803210767

Table S4. Experimental and computed chemical shifts of **7**, **7a** and **7b**.

Position	Exp.**7**	**7a**	Residue	**7b**	Residue
1	53	52.51929109	0.480708911	56.39563554	-3.395635541
2	174	175.5288602	-1.528860224	175.7706018	-1.770601774
3	127.1	128.1285239	-1.02852393	122.9261794	4.173820557
4	196.8	196.9528939	-0.152893885	193.2330898	3.566910193
5	69.6	71.8402837	-2.240283696	74.9403582	-5.340358197
6	47.7	52.98843816	-5.288438162	54.27774252	-6.577742521
7	43.5	44.20971037	-0.709710369	40.47934573	3.020654266
8	40.3	42.00599777	-1.705997767	41.31762317	-1.017623165
9	208.2	212.7336373	-4.533637252	211.7052974	-3.505297442
10	194.8	194.1532241	0.646775922	196.5267486	-1.726748574
11	131	128.7279917	2.272008336	128.4429064	2.557093588
12	116.7	112.3510836	4.348916448	112.549242	4.150757951
13	146.8	143.7307659	3.069234125	143.4882586	3.311741372
14	153.1	151.0723289	2.027671121	151.2337338	1.866266175
15	116	113.515104	2.48489602	113.7887188	2.211281225
16	124.4	127.556822	-3.156821963	129.2936799	-4.893679904
17	22.8	24.03751701	-1.237517008	24.60480413	-1.804804131
18	39.8	37.18177355	2.618226451	39.41576053	0.384239465
19	71.9	72.29629813	-0.396298132	73.56333013	-1.663330132
20	29.2	33.28850284	-4.088502835	29.48195997	-0.281959973
21	29.2	27.45875778	1.741242216	29.67990894	-0.479908941
22	27.2	26.78716196	0.412838039	23.32632455	3.873675452
23	23.1	22.93151059	0.168489409	17.18006117	5.919938832
24	33.5	33.0391485	0.460851497	37.08912649	-3.589126494
25	78.6	75.13783923	3.462160775	76.24979393	2.350206068
26	74.4	76.34191177	-1.941911768	75.18725007	-0.787250071
27	24.6	26.80327308	-2.203273085	22.06715706	2.532842941
28	25.9	23.18271441	2.717285591	32.079058	-6.179058002
29	29.2	27.40843058	1.791569423	27.76668625	1.433313745
30	45	42.12925307	2.870746925	37.55640345	7.443596555
31	89.2	86.19531257	3.004687431	89.25118816	-0.051188156
32	21.7	26.83843167	-5.13843167	23.54699363	-1.846993626
33	29.1	28.90791707	0.192082932	28.3549478	0.745052203
34	26.6	25.61966725	0.980332755	30.29126926	-3.691269263
35	42.8	40.89195999	1.908040006	37.51579125	5.284208753

36	71.4	72.14425218	-0.74425218	74.64698476	-3.246984763
37	29.3	32.94685954	-3.646859543	27.74055813	1.559441873
38	29.7	27.61655086	2.083449136	34.23548054	-4.535480544

Table S5. Experimental and computed chemical shifts of **15**,**15a**,**15b**,**15c** and **15d**.

Position	Exp.**15**	**15a**	Residue	**15b**	Residue
1	66.3	69.92226967	-3.622269673	69.18662347	-2.886623466
2	193.1	192.6162254	0.483774602	193.8880441	-0.788044118
3	119.2	119.0265441	0.173455859	118.4007772	0.79922283
4	177	179.0127405	-2.012740513	177.9438854	-0.943885428
5	63.6	64.5443827	-0.944382704	64.31483687	-0.714836873
6	48.8	53.79284257	-4.992842565	52.39651737	-3.596517365
7	47.3	53.58634864	-6.286348641	53.78533416	-6.485334157
8	38.2	45.54597943	-7.345979431	49.91195212	-11.71195212
9	207.7	209.223302	-1.523302011	211.0956156	-3.395615569
10	172.8	167.7578594	5.042140594	166.9384418	5.861558212
11	118	116.9557353	1.044264688	116.4129118	1.587088216
12	109.6	107.1806632	2.419336819	106.5227071	3.0772929
13	146.9	141.5214881	5.378511941	140.8202364	6.079763586
14	155.9	149.4567821	6.443217925	148.7654739	7.134526129
15	104.5	101.5758831	2.924116925	101.3501213	3.149878715
16	150.6	148.8200764	1.779923563	148.2259471	2.374052934
17	27.4	27.33669856	0.063301439	26.69457753	0.705422465
18	119.9	118.9958491	0.904150902	120.1348239	-0.234823926
19	135.3	140.4819942	-5.181994179	137.5682764	-2.268276448
20	26.2	25.52444014	0.675559861	24.38258931	1.817410688
21	18.8	17.84423251	0.955767486	16.52436616	2.275633835
22	26.9	25.86439496	1.035605045	23.36099367	3.539006331
23	22.4	21.8317014	0.568298601	21.9887358	0.411264203
24	30.1	27.83849997	2.261500034	25.18034844	4.919651563
25	124.6	128.6909602	-4.090960161	128.4807213	-3.880721269
26	135.3	133.6209304	1.679069561	132.8629588	2.437041247
27	26.3	25.03957393	1.26042607	24.20424489	2.095755108
28	18.2	16.32141199	1.878588012	15.37344837	2.826551628
29	40	37.06442912	2.935570879	41.92382308	-1.923823084
30	41.5	40.28288615	1.217113851	40.16918539	1.330814607
31	49.4	51.31356835	-1.913568355	57.59211236	-8.192112359
32	29.4	26.04019384	3.359806163	33.46663284	-4.066632835
33	31.8	28.71508881	3.084911194	27.43211988	4.367880123
34	146.9	152.6232788	-5.723278843	154.1943866	-7.294386636
35	109.6	111.95533	-2.355330014	109.4496453	0.150354739
36	24.3	20.61535019	3.684649812	24.11624758	0.183752423

37	24.7	27.72139693	-3.021396934	18.12736289	6.572637108
38	25.7	27.9386678	-2.238667802	31.01297394	-5.312973936

Position	Exp.**15**	**15c**	Residue	**15d**	Residue
1	66.3	66.25195895	0.048041048	68.40447626	-2.104476256
2	193.1	196.9531897	-3.853189714	195.8336543	-2.733654287
3	119.2	115.8534034	3.346596569	119.7645066	-0.564506623
4	177	178.7443943	-1.74439432	174.7269301	2.273069887
5	63.6	61.97812752	1.621872479	63.62311811	-0.023118111
6	48.8	51.73222249	-2.932222494	51.82805076	-3.028050759
7	47.3	55.59567262	-8.295672615	56.79575826	-9.495758262
8	38.2	49.55735236	-11.35735236	44.68935353	-6.48935353
9	207.7	210.0054131	-2.305413131	210.6498072	-2.949807197
10	172.8	167.1273791	5.672620941	165.6566804	7.143319584
11	118	115.9626214	2.03737857	116.7109183	1.289081729
12	109.6	105.7873074	3.81269257	105.9438257	3.656174317
13	146.9	140.5834121	6.316587897	141.11185	5.788150026
14	155.9	148.4945151	7.405484865	148.8138305	7.086169509
15	104.5	100.0353286	4.464671429	101.4741989	3.025801109
16	150.6	148.1464396	2.45356042	148.6542713	1.945728684
17	27.4	29.32194173	-1.92194173	26.28449807	1.115501932
18	119.9	123.6375159	-3.73751591	120.4389889	-0.538988868
19	135.3	136.0850876	-0.785087565	138.2668705	-2.96687047
20	26.2	23.41798208	2.782017919	24.22194949	1.978050514
21	18.8	13.76387633	5.036123673	15.74512203	3.054877968
22	26.9	22.25723924	4.642760763	27.18295412	-0.282954119
23	22.4	24.90407446	-2.504074458	21.96497509	0.435024907
24	30.1	28.95619253	1.143807474	28.07983428	2.020165723
25	124.6	124.665977	-0.065977049	123.1662689	1.433731144
26	135.3	137.7778436	-2.477843563	138.4558791	-3.155879148
27	26.3	23.35207743	2.947922567	23.95473711	2.345262887
28	18.2	14.86143544	3.338564565	14.75536267	3.444637325
29	40	40.19201715	-0.192017147	47.29568372	-7.295683723
30	41.5	39.25795434	2.242045661	39.18978211	2.310217894
31	49.4	55.48506956	-6.08506956	52.19257453	-2.79257453
32	29.4	32.6322785	-3.232278498	28.91860338	0.481396623
33	31.8	36.35831254	-4.558312542	33.83795066	-2.037950661
34	146.9	154.0700823	-7.170082264	154.0611605	-7.161160526
35	109.6	108.4724228	1.127577206	112.1694898	-2.56948985
36	24.3	25.33857308	-1.038573079	18.09625604	6.203743963
37	24.7	18.03349879	6.666501208	21.53652916	3.163470842
38	25.7	28.54980982	-2.849809819	29.70329965	-4.003299645

Table S6. Statistics of Ordinary Least Squares Linear Regression (OLS-LR) of

experimental and computed ^{13}C-NMR chemical shifts.

Compound	CMAD[a]	CLAD[b]	R^2	RMSD	*F*	*P* value
**3a**	2.25	5.56	0.9978	2.9252	16494.21	< 0.01
**3b**	1.97	4.95	0.9983	2.594	20984.62	< 0.01
**7a**	2.09	5.29	0.998	2.5883	17675.72	< 0.01
**7b**	2.97	7.44	0.9961	3.5969	9135.65	< 0.01
**15a**	2.7	7.35	0.9968	3.3928	11126.96	< 0.01
**15b**	3.35	11.71	0.9947	4.3519	6748.84	< 0.01
**15c**	3.53	11.36	0.9945	4.4132	6561.74	< 0.01
**15d**	3.17	9.5	0.9955	4.0164	7929.88	< 0.01

[a]CMAD = corrected mean absolute deviation, computed as $(1/n)\sum_{i}^{n}|\delta_{\text{calc}}-\delta_{\text{exp}}|$, where δ_{calc} and δ_{exp} refer to the calculated and experimental chemical shifts. [b]CLAD = corrected largest absolute deviation, computed as $\max(|\delta_{\text{calc}}-\delta_{\text{exp}}|)$.

3.2 DP4+ analysis

3.2.1 DP4$^+$ analysis of compound **3** with two possible structures of **3a** and **3b**

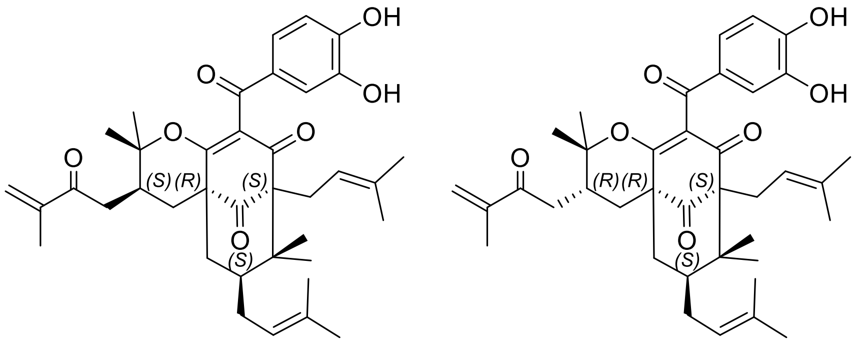

3a **3b**

(DP4$^+$： 95.25%**)-3a** **(DP4$^+$：** 4.75%**)-3b**

Figure S60. DP4+ probability for compounds **3a** and **3b** based on its ^{1}H and ^{13}C-NMR date.

A	B	C	D	E	F	G	H
Functional		Solvent?		Basis Set		Type of Data	
mPW1PW91		PCM		6-311G(d, p)		Unscaled Shifts	
		Isomer 1	Isomer 2	Isomer 3	Isomer 4	Isomer 5	Isomer 6
sDP4+ (H data)		99.69%	0.31%	–	–	–	–
sDP4+ (C data)		0.01%	99.99%	–	–	–	–
sDP4+ (all data)		3.59%	96.41%	–	–	–	–
uDP4+ (H data)		99.98%	0.02%	–	–	–	–
uDP4+ (C data)		9.47%	90.53%	–	–	–	–
uDP4+ (all data)		99.81%	0.19%	–	–	–	–
DP4+ (H data)		100.00%	0.00%	–	–	–	–
DP4+ (C data)		0.00%	100.00%	–	–	–	–
DP4+ (all data)		95.25%	4.75%	–	–	–	–

Functional		Solvent?		Basis Set		Type of Data	
mPW1PW91		PCM		6-311G(d,p)		Unscaled Shifts	
		DP4+	95.25%	4.75%	-	-	-
Nuclei	sp2?	xperimenta	Isomer 1	Isomer 2	Isomer 3	Isomer 4	Isomer 5
C		51.4	53.13227772	54.2532464			
C	x	170.9	181.8959056	180.773855			
C	x	126.3	133.3673547	130.543085			
C	x	194.3	204.3041585	203.719372			
C		69	75.31457016	75.0081882			
C		46.7	54.12057462	53.8571158			
C		47	49.8586742	49.7957382			
C		39.9	41.90391464	39.5036682			
C	x	206.9	222.6021363	222.363605			
C	x	192.3	201.3446601	201.667579			
C	x	131.3	133.4976306	133.568817			
C	x	115.9	116.6694549	116.605167			
C	x	146	149.2381387	149.224648			
C	x	151.3	157.0777185	157.050073			
C	x	115.6	117.94859	117.971693			
C	x	123.6	132.6559252	132.452913			
C		26.4	28.78651242	28.7266488			
C	x	121.6	127.411826	127.417903			
C	x	134.2	146.1202563	146.032767			
C		26.4	28.11213078	28.1256166			
C		18.3	18.89874536	18.8920909			
C		27	27.22822792	27.1287654			
C		22.8	22.64032896	22.5793543			
C		29.8	31.9525315	31.8387178			
C	x	126.4	132.4435862	132.60255			
C	x	133.3	145.2902012	144.844632			
C		26.2	28.0188191	27.9663457			
C		18.6	19.21503572	19.3588656			
C		29.3	36.05218636	30.9828102			
C		38.9	39.6285031	39.2744226			
C		86	89.29504008	89.1670548			
C		22.2	29.22246264	21.608973			
C		29	27.04917908	28.2655562			
C		39.7	48.23073998	43.891832			
C	x	199.8	209.8611147	206.780998			
C	x	145.5	149.9425086	148.47624			
C	x	125.5	134.1949359	134.745854			
C		17.9	21.22099962	21.6443528			
H		1.52	1.50218832	1.44208576			
H		2.33	2.61573684	2.46849708			
H		2.02	1.88938328	1.6015906			
H	x	7.39	7.92662055	7.91751916			
H	x	6.84	7.00962437	7.02074368			
H	x	7.14	7.38992694	7.42779128			
H		2.63	2.36939502	2.34917588			
H		2.43	2.85364029	2.82296492			
H	x	5.01	5.71623703	5.7056932			
H		1.57	1.42632556	1.41180423			
H		1.56	1.69446735	1.69378995			
H		0.97	0.98871364	0.92632631			
H		1.14	1.09651568	1.08343903			
H		2.75	2.90488696	2.82684624			
H		1.29	1.98916887	1.96596612			
H	x	5.01	5.20499498	5.16529228			
H		1.68	1.74586395	1.72549997			
H		1.69	1.93372005	1.88318365			
H		3.01	3.10043546	2.09296576			
H		1.19	1.57676804	1.5753682			
H		2.1	2.11692666	2.13610844			
H		1.3	0.91074536	1.3634688			
H		0.91	1.107545	0.8289926			
H		2.8	2.76057616	3.12451192			
H		2.73	2.70644186	1.76007532			
H	x	6.09	6.44078463	6.61966796			
H	x	5.88	5.78419309	5.81782316			
H		1.89	2.09628222	2.14276564			

3.2.2 DP4+ analysis of compound **7** with two possible structures of **7a** and **7b**.

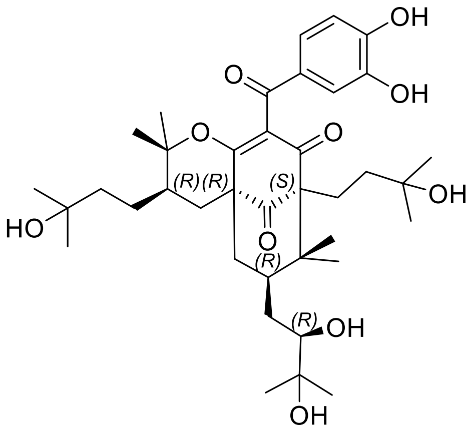

7a

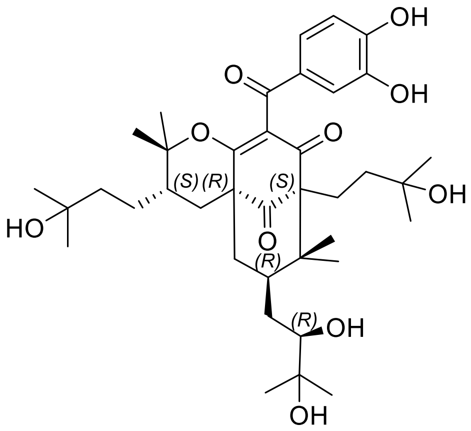

7b

(DP4^{+}: 100.00%**)-7a** **(DP4^{+}:** 0.00%**)-7b**

Figure S61. DP4+ probability for compounds **7a** and **7b** based on its ^{1}H and ^{13}C-NMR date.

Functional	Solvent?	Basis Set	Type of Data
mPW1PW91	PCM	6-311G(d,p)	Unscaled Shifts

	Isomer 1	Isomer 2	Isomer 3	Isomer 4	Isomer 5	Isomer 6
sDP4+ (H data)	100.00%	0.00%	-	-	-	-
sDP4+ (C data)	100.00%	0.00%	-	-	-	-
sDP4+ (all data)	100.00%	0.00%	-	-	-	-
uDP4+ (H data)	100.00%	0.00%	-	-	-	-
uDP4+ (C data)	100.00%	0.00%	-	-	-	-
uDP4+ (all data)	100.00%	0.00%	-	-	-	-
DP4+ (H data)	100.00%	0.00%	-	-	-	-
DP4+ (C data)	100.00%	0.00%	-	-	-	-
DP4+ (all data)	100.00%	0.00%	-	-	-	-

Functional		Solvent?		Basis Set		Type of Data	
mPW1PW91		PCM		6-311G(d,p)		Unscaled Shifts	
		DP4+	100.00%	0.00%	-	-	-
Nuclei	sp2?	xperimenta	Isomer 1	Isomer 2	Isomer 3	Isomer 4	Isomer 5
C		53	53.9690859	57.5198			
C	x	174	183.047284	183.6195			
C	x	127.1	133.308471	127.7982			
C	x	196.8	205.528264	202.0657			
C		69.6	74.2432721	77.1092			
C		47.7	54.4613782	55.2826			
C		43.5	45.2495553	40.7069			
C		40.3	42.9371234	41.5924			
C	x	208.2	222.087544	221.5785			
C	x	194.8	202.590474	205.5449			
C	x	131	133.937513	133.6257			
C	x	116.7	116.752656	116.8367			
C	x	146.8	149.680443	149.5186			
C	x	153.1	157.384199	157.7004			
C	x	116	117.974103	118.146			
C	x	124.4	132.708564	134.5244			
C		22.8	24.0821747	23.9381			
C		39.8	37.874898	39.5834			
C		71.9	74.7217839	75.6546			
C		29.2	33.7895543	29.09			
C		29.2	27.672201	29.2991			
C		27.2	26.9674723	22.5876			
C		23.1	22.9216039	16.0951			
C		33.5	33.5278981	37.1257			
C		78.6	77.7035113	78.4924			
C		74.4	78.9669863	77.37			
C		24.6	26.9843783	21.2575			
C		25.9	23.1852007	31.8334			
C		29.2	27.619391	27.2781			
C		45	43.0664595	37.6193			
C		89.2	89.3065007	92.2262			
C		21.7	27.0212714	22.8207			
C		29.1	29.192854	27.8995			
C		26.6	25.7423797	29.9449			
C		42.8	41.768125	37.5764			
C		71.4	74.5622368	76.7993			
C		29.3	33.4310561	27.2505			
C		29.7	27.8377788	34.1113			
H		1.82	1.98661068	2.8068			
H		2.28	2.31049742	2.9715			
H		2.2	2.59843845	1.3269			
H	x	7.22	7.94850965	7.9473			
H	x	6.76	7.09596129	7.1079			
H	x	7.1	7.51289634	7.6052			
H		1.89	1.98931041	1.71865			
H		1.43	1.71355126	1.3712			
H		1.08	0.8729254	1.1985			
H		1.16	1.15366845	1.0847			
H		1.17	1.05657923	1.22923333			
H		1.02	0.98618192	0.67986667			
H		1.14	1.08750249	0.9738			
H		2.16	2.23149484	1.6747			
H		1.44	2.15120515	1.3456			
H		3.18	3.58199748	3.849			
H		1.21	1.43437822	1.20943333			
H		1.22	1.16905403	1.23756667			
H		3.13	2.88301274	1.9804			
H		1.09	1.60966175	1.6423			
H		1.27	1.78920423	3.0408			
H		1.29	1.08564139	0.94443333			
H		0.95	1.20709479	1.3801			
H		1.52	1.70446732	1.747			
H		1.09	1.68051331	1.1997			
H		1.8	2.04278535	1.9276			
H		1.42	1.33627864	1.449			
H		1.22	1.23273651	1.235			
H		1.22	1.22473469	1.1873			

3.2.3 DP4+ analysis of compound**15** with two possible structures of **15a**,**15b**,**15c** and **15d**.

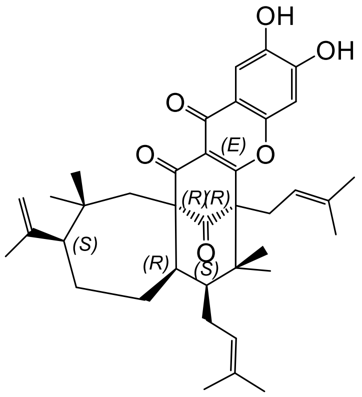

15a

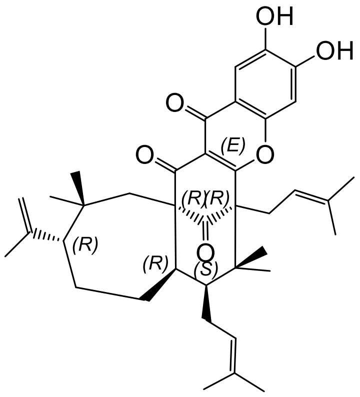

15b

(DP4$^+$： 100.00%)-15a **(DP4$^+$： 0.00%)-15b**

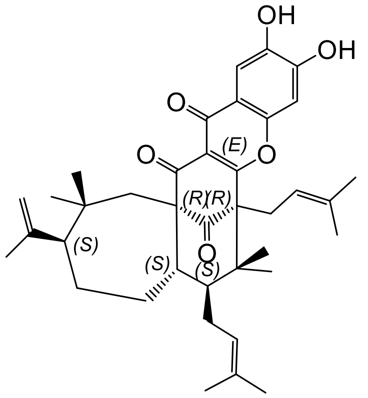

15c

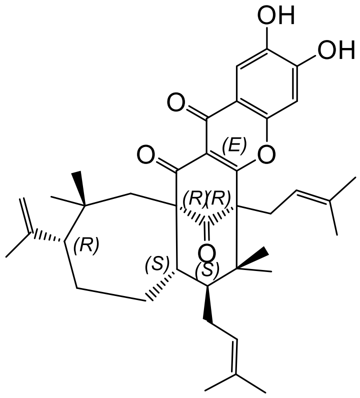

15d

(DP4$^+$： 0.00%)-15c **(DP4$^+$： 0.00%)-15d**

Figure S62. DP4+ probability for compounds **15a**,**15b**,**15c** and **15d** based on its ^{1}H and ^{13}C-NMR date.

A	B	C	D	E	F	G	H
Functional		Solvent?		Basis Set		Type of Data	
mPW1PW91		PCM		6-311G(d,p)		Unscaled Shifts	
		Isomer 1	Isomer 2	Isomer 3	Isomer 4	Isomer 5	Isomer 6
sDP4+ (H data)		0.10%	99.90%	0.00%	0.00%	-	-
sDP4+ (C data)		100.00%	0.00%	0.00%	0.00%	-	-
sDP4+ (all data)		100.00%	0.00%	0.00%	0.00%	-	-
uDP4+ (H data)		0.00%	100.00%	0.00%	0.00%	-	-
uDP4+ (C data)		100.00%	0.00%	0.00%	0.00%	-	-
uDP4+ (all data)		99.50%	0.50%	0.00%	0.00%	-	-
DP4+ (H data)		0.00%	100.00%	0.00%	0.00%	-	-
DP4+ (C data)		100.00%	0.00%	0.00%	0.00%	-	-
DP4+ (all data)		100.00%	0.00%	0.00%	0.00%	-	-

Functional		Solvent?		Basis Set		Type of Data	
mPW1PW91		PCM		6-311G(d,p)		Unscaled Shifts	
		DP4+	100.00%	0.00%	0.00%	0.00%	-
Nuclei	sp2?	xperimenta	Isomer 1	Isomer 2	Isomer 3	Isomer 4	Isomer 5
C		66.3	74.1842	74.1427526	72.290804	70.9106	
C	x	193.1	201.6946	203.445775	206.50087	200.2891	
C	x	119.2	125.2161	125.172956	123.22386	123.0563	
C	x	177	187.5571	186.913262	187.803237	178.8595	
C		63.6	68.5952	69.0911925	67.9022363	66.0561	
C		48.8	57.4216	56.7330756	57.3812674	54.0806	
C		47.3	57.207	58.173141	61.3484365	59.1243	
C		38.2	48.851	54.1568275	55.1480102	46.8327	
C	x	207.7	218.9536	221.288322	219.903496	215.3319	
C	x	172.8	175.8604	175.501707	175.874349	169.6505	
C	x	118	123.064	123.111736	123.33601	119.956	
C	x	109.6	112.9052	112.856573	112.887527	109.0242	
C	x	146.9	148.5941	148.419714	148.617776	144.7302	
C	x	155.9	156.8409	156.658139	156.741262	152.55	
C	x	104.5	107.0804	107.493114	106.98113	104.4862	
C	x	150.6	156.1792	156.098703	156.383842	152.388	
C		27.4	29.9269	30.0827096	34.3693551	28.1463	
C	x	119.9	125.1842	126.97099	131.216947	123.7411	
C	x	135.3	147.5138	145.047754	143.998689	141.8417	
C		26.2	28.0435	27.6854068	28.3068964	26.0522	
C		18.8	20.0618	19.5372077	18.3936144	17.4457	
C		26.9	28.3968	26.6261133	27.114992	29.0585	
C		22.4	24.2058	25.2032178	29.8328848	23.7607	
C		30.1	30.4484	28.512604	33.9937869	29.9691	
C	x	124.6	135.2599	135.624859	132.273018	126.5101	
C	x	135.3	140.3834	140.168806	145.736889	142.0336	
C		26.3	27.5396	27.5004813	28.2392225	25.7809	
C		18.2	18.4792	18.3438199	19.5206388	16.4408	
C		40	40.0365	45.8739288	45.531251	49.4789	
C		41.5	43.3813	44.0545433	44.5721122	41.249	
C		49.4	54.845	62.120393	61.2348642	54.4507	
C		29.4	28.5795	37.1046602	37.7685619	30.8207	
C		31.8	31.3594	30.8474679	41.5946259	35.8153	
C	x	146.9	160.1317	162.287384	162.466513	157.8776	
C	x	109.6	117.8673	115.891518	115.644728	115.3451	
C		24.3	22.9417	27.4092368	30.279048	19.8328	
C		24.7	30.3267	21.1993565	22.7778601	23.3257	
C		25.7	30.5525	34.5604589	33.5764944	31.6174	
H		1.56	1.3865	1.4936471	1.35975196	1.1223	
H		2.11	2.7779	2.7603247	2.94723184	2.6314	
H	x	8.1	7.8417	7.78837504	7.83292473	7.2827	
H	x	7.45	7.2789	7.23967738	7.34704698	6.809	
H		3.02	2.97415	2.99215273	2.98722277	2.49185	
H	x	5.04	5.1035	5.34012692	6.57849535	5.0441	
H		1.37	1.50406667	1.45840152	1.81849331	1.44383333	
H		1.78	1.86833333	1.83123481	1.91972629	1.12186667	
H		1.15	1.23986667	1.22151641	1.18723009	0.75453333	
H		1.27	1.3869	1.19481852	1.06628281	0.84513333	
H		2.09	1.6926	1.89373614	2.0123687	1.2039	
H		1.97	1.9176	1.7625388	2.16768009	1.8646	
H	x	5.01	5.0699	5.12975576	5.11609445	5.2355	
H		1.64	1.42673333	1.55699063	1.72628203	1.18406667	
H		1.51	1.56236667	1.23601206	1.57521188	1.35773333	
H		2.39	2.5059	2.2746848	2.79595125	1.5548	
H		2.13	1.7607	2.1372109	1.22589032	1.3453	
H		2.48	2.279	2.15483918	1.81138351	2.3058	
H		2.23	2.0688	1.737862	1.6693914	1.4435	
H		1.91	1.4441	1.58370682	1.54885735	1.0725	
H		2.29	1.7983	2.1987156	1.57725916	1.686	
H		2.21	1.2364	1.28893054	1.37061187	0.8684	
H	x	4.8	5.0516	5.13115612	5.20386464	4.6839	
H	x	4.9	4.8931	4.92063532	4.88295148	4.8121	
H		1.65	1.78716667	1.84834153	1.87067646	1.42676667	
H		1.01	1.0911	1.01722958	1.24528838	0.8582	
H		1.2	0.8571	1.05279026	0.84042198	0.49056667	

4.Experimental and calculated ECD spectra

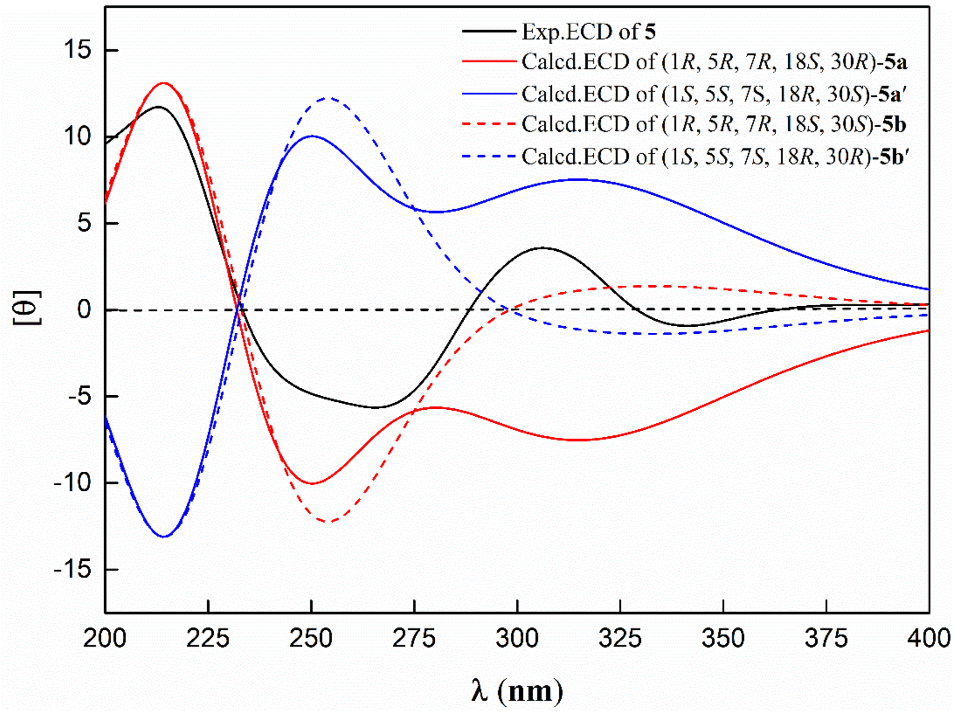

Figure S63. Calculated ECD spectra of compound **5** was compared with the experimental.

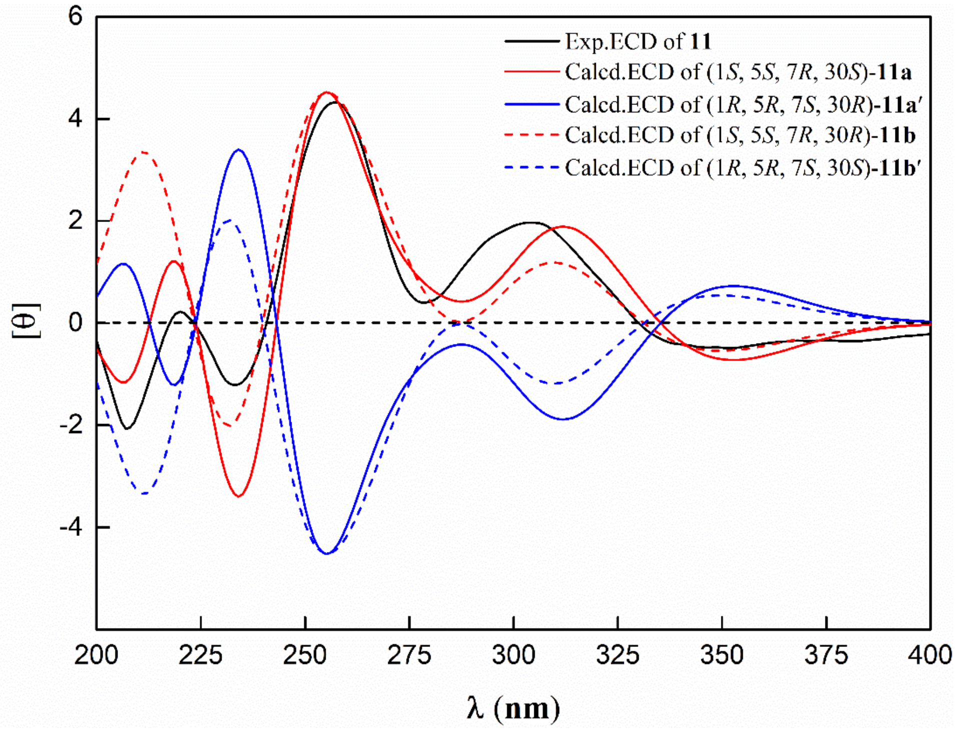

Figure S64. Calculated ECD spectra of compound **11** was compared with the experimental.

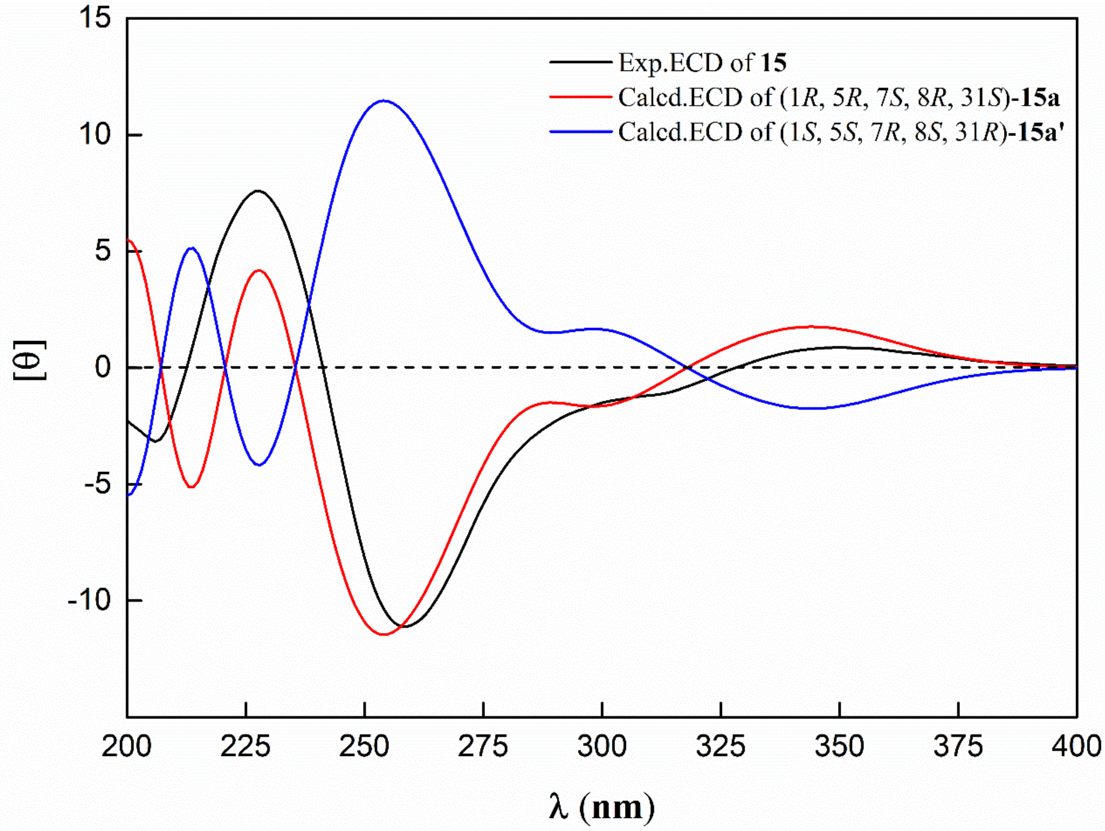

Figure S65. Calculated ECD spectra of compound **15** was compared with the experimental.

Table S7. ^{1}H-NMR and ^{13}C-NMR of compounds **1-2** (in pyridine-d_5)

No.	^{13}C-NMR		^{1}H-NMR	
	**1**	**2**	**1**	**2**
1	53.8	54.0		
2	170.8	170.8		
3	129.1	129.2		
4	195.0	195.0		
5	71.3	71.3		
6	46.7	46.7		
7	43.7	42.6	2.43 m	2.41 m
8	42.9	42.7	2.43 m, 1.75 m	2.41 m, 1.73 m
9	207.3	207.2		
10	193.2	193.1		
11	130.8	130.8		
12	116.8	116.7	8.14 s	8.14 s
13	147.9	147.9		
14	153.7	153.7		
15	116.4	116.3	7.29 d (8.0)	7.26 d (8.5)
16	123.4	124.3	7.76 d (8.0)	7.68 m
17	25.9	25.8	2.77 dd (14.0, 6.0)	2.78 dd (13.5, 6.0)
			2.97 dd (14.0, 7.5)	2.96 dd (13.5, 7.5)
18	121.9	121.9	5.39 br s	5.40 br s
19	134.2	134.2		
20	18.7	18.3	1.72 s 3H	1.76 s 3H
21	26.5	26.6	1.55 s 3H	1.60 s 3H
22	16.5	16.5	0.82 s 3H	0.87 s 3H
23	22.7	22.7	1.26 s 3H	1.26 s 3H
24	28.3	28.4	2.24 m, 1.82 m	2.24 m, 1.83 m
25	122.8	123.7	5.11 br s	5.10 s
26	133.5	134.2		
27	18.3	18.7	1.61 s 3H	1.62 s 3H
28	26.1	26.1	1.61 s 3H	1.62 s 3H
29	28.5	27.9	3.25 dd (14.5, 3.5)	3.20 dd (14.0, 3.0)
			1.17 t (14.0)	1.10 m
30	43.7	42.7	1.65 m	1.53 m
31	87.5	87.8		
32	21.6	21.5	1.12 s 3H	1.09 s 3H
33	29.1	28.9	1.08 s 3H	1.03 s 3H
34	30.2	29.1	1.94 m, 1.80 m	1.38 m, 1.10 m
35	122.8	35.9	5.11 br s	2.24 m, 1.95 m
36	133.7	145.6		
37	26.2	115.5	1.68 s 3H	4.87 d (7.0)
				4.86 d (7.0)
38	18.2	22.7	1.55 s 3H	1.67 s 3H

Table S8. ^{1}H-NMR and ^{13}C-NMR of compound **4** (in pyridine-d_5)

No	^{13}C-NMR	^{1}H-NMR	No	^{13}C-NMR	^{1}H-NMR
1	59.9		20	26.5	1.67 (3H, s)
2	191.5		21	19.0	1.65 (3H, s)
3	119.3		22	27.6	1.12 (3H, s)
4	190.3		23	23.6	1.39 (3H, s)
5	68.1		24	30.4	2.18 (2H, m)
6	48.9		25	125.0	4.89 (1H, m)
7	47.6	1.46 (1H, m)	26	132.6	
8	43.0	2.26 (2H, br d, *J* = 13.8Hz)	27	26.3	1.66 (3H, s)
9	211.1		28	19.1	1.65 (3H, s)
10	196.2		29	37.7	2.27 (2H, m)
11	130.7		30	41.0	2.47 (1H, m)
12	117.7	7.90 (1H, d, *J* = 2.0 Hz)	31	149.8	
13	147.0		32	113.3	4.32 (2H, br s)
14	153.3		33	18.8	1.63 (3H, s)
15	116.2	7.23 (1H, d, *J* = 8.0 Hz)	34	40.5	1.62 (2H, m)
16	126.3	7.74 (1H, dd, *J* = 8.0, 2.0 Hz)	35	73.3	3.69 (1H, t, *J* = 7.1 Hz)
17	26.8	2.73 (2H, dd, *J* = 13.2, 9.0 Hz)	36	149.2	
18	122.3	5.05 (1H, m)	37	109.7	4.89 (2H, br s)
19	133.9		38	18.5	1.93 (3H, s)

Table S9. ^{1}H-NMR and ^{13}C-NMR of compound **6** (in pyridine-d_5)

No	^{13}C-NMR	^{1}H-NMR	No	^{13}C-NMR	^{1}H-NMR
1	61.4		20	26.2	1.28 (3H, s)
2	195.1		21	26.3	1.29 (3H, s)
3	118.6		22	27.5	1.18 (3H, s)
4	175.6		23	24.0	1.21 (3H, s)
5	68.4		24	30.7	3.10 (1H, m); 2.63 (1H, m)
6	48.0		25	126.3	5.06 (1H, t, *J* = 7.0Hz)
7	46.3	1.71 (1H, m)	26	133.3	
8	42.2	2.40 (1H, m); 2.21 (1H, m)	27	18.4	1.81 (3H, s)
9	208.6		28	26.4	1.67 (3H, s)
10	191.8		29	37.5	2.25 (1H, m); 2.08 (1H, m)
11	131.3		30	44.2	2.84 (1H, m)
12	116.9	8.15 (1H, d, *J* = 2.0 Hz)	31	148.5	
13	147.7		32	113.7	4.86 (1H, s); 4.80 (1H, s)
14	153.9		33	18.4	1.67 (3H, s)
15	116.1	7.24 (1H, d, *J* = 8.5 Hz)	34	32.5	1.68 (1H, m); 1.63 (1H, m)
16	125.6	7.81 (1H, dd, *J* = 8.5, 2.0 Hz)	35	36.3	1.96 (1H, m); 1.90 (1H, m)
17	27.4	3.20 (1H, dd, *J* = 14, 9.5 Hz)	36	146.7	
		2.46 (1H, dd, *J* = 14, 6.5 Hz)			
18	93.7	4.78 (1H, br s)	37	23.3	1.69 (3H, s)
19	71.0		38	110.4	4.83 (1H, s); 4.76 (1H, s)

Table S10. ^{1}H-NMR and ^{13}C-NMR of compounds **9** and **10** (in pyridine-d_5)

No	9		10	
	^{13}C-NMR	^{1}H-NMR	^{13}C-NMR	^{1}H-NMR
1	52.2		52.6	
2	171.1		171.1	
3	127.1		127.1	
4	195.0		195.0	
5	69.4		69.4	
6	46.8		48.0	
7	43.1	2.29 (1H, m)	43.7	2.29 (1H, m)
8	40.2	2.28 (1H, m)	45.4	2.28 (1H, m)
		2.58 (1H, br d, *J* = 13.3Hz)		2.58 (1H, br d, *J* = 13.3Hz)
9	207.9		207.9	
10	193.5		193.5	
11	130.7		130.7	
12	116.5	8.07 (1H, d, *J* = 2.0Hz)	116.5	8.09 (1H, d, *J* = 2.0Hz)
13	147.7		147.7	
14	153.8		153.8	
15	116.6	7.27 (1H, d, *J* = 8.5Hz)	116.6	7.28 (1H, d, *J* = 8.0Hz)
16	125.4	7.71 (1H, dd, *J* = 2.0, 8.5Hz)	125.4	7.71 (1H, dd, *J* = 2.0, 8.0Hz)
17	26.6	2.78 (1H, m)	26.6	2.78 (1H, m)
		2.95 (1H, dd, *J* = 13.5, 7.4Hz)		2.95 (1H, dd, *J* = 13.5, 7.4Hz)
18	121.7	5.41 (1H, br t, *J* = 6.1Hz)	121.7	5.42 (1H, br t, *J* = 6.1Hz)
19	134.2		134.2	
20	26.5	1.57 (3H, s)	26.5	1.57 (3H, s)
21	18.7	1.71 (3H, s)	18.7	1.71 (3H, s)
22	27.2	1.08 (3H, s)	27.2	1.08 (3H, s)
23	23.1	1.43 (3H, s)	23.1	1.43 (3H, s)
24	33.6	2.80 (1H, m)	33.6	2.80 (1H, m)
		2.05 (1H, dd, *J* = 13.9, 11.4Hz)		2.05 (1H, dd, *J* = 13.9, 11.4Hz)
25	78.5	3.80 (1H, br d, *J* = 10.4Hz)	83.0	3.68 (1H, br d, *J* = 10.4Hz)
26	73.2		73.2	
27	27.1	1.74 (3H, s)	27.1	1.74 (3H, s)
28	25.0	1.66 (3H, s)	25.0	1.66 (3H, s)
29	29.3	1.14 (1H, m)	29.3	1.14 (1H, m)
		3.30 (1H, dd, *J* = 14.3, 3.3Hz)		3.25 (1H, dd, *J* = 14.3, 3.3Hz)
30	43.8	1.67 (1H, m)	43.8	1.67 (1H, m)
31	87.1		87.1	
32	21.4	1.22 (3H, s)	21.4	1.22 (3H, s)
33	29.3	1.06 (3H, s)	29.3	1.06 (3H, s)
34	30.3	1.78 (1H, m), 1.98 (1H, m)	30.3	1.78 (1H, m), 1.98 (1H, m)
35	122.7	5.06 (1H, br d, *J* = 6.1Hz)	122.7	5.04 (1H, br d, *J* = 6.1Hz)
36	133.6		133.6	
37	26.1	1.67 (3H, s)	26.1	1.67 (3H, s)
38	18.2	1.54 (3H, s)	18.2	1.54 (3H, s)

Table S11. ^{1}H-NMR and ^{13}C-NMR of compound **12** (in pyridine-d_5)

No	^{13}C-NMR	^{1}H-NMR	No	^{13}C-NMR	^{1}H-NMR
1	56.1		20	18.8	1.83 (3H, s)
2	177.3		21	26.4	1.54 (3H, s)
3	120.1		22	27.3	1.10 (3H, s)
4	192.7		23	22.7	1.27 (3H, s)
5	72.1		24	30.5	2.13 (1H, m); 2.08 (1H, m)
6	50.2		25	125.0	4.83 (1H, br s)
7	46.6	1.59 (1H, m)	26	132.9	
8	42.2	2.52 (1H, d, *J* = 14 Hz)	27	26.1	1.54 (3H, s)
		2.21 (1H, dd, *J* = 14.5, 7.0 Hz)			
9	208.8		28	17.9	1.10 (3H, s)
10	172.4		29	37.1	2.31 (1H, m)
					2.05 (1H, m)
11	119.0		30	44.3	2.61 (1H, m)
12	110.1	8.19 (1H, s)	31	147.6	
13	148.3		32	112.9	4.41 (1H, s); 4.36 (1H, s)
14	155.1		33	18.0	1.66 (3H, s)
15	104.6	7.45 (1H, s)	34	32.7	1.76 (1H, m); 1.58 (1H, m)
16	147.6		35	35.9	1.94 (2H, m)
17	26.9	3.01 (1H, dd, *J* = 17, 7.0 Hz)	36	146.1	
		2.86 (1H, dd, *J* = 13.5, 6.5 Hz)			
18	121.4	5.23 (1H, br s)	37	22.9	1.71 (3H, s)
19	134.3		38	110.1	4.77 (2H, s)

Table S12. ^{1}H-NMR and ^{13}C-NMR of compound **13** (in pyridine-d_5)

No	^{13}C-NMR	^{1}H-NMR	No	^{13}C-NMR	^{1}H-NMR
1	67.0		18	119.7	5.00 (1H, br s)
2	192.3		19	135.3	
3	118.5		20	18.7	1.75 (3H, s)
4	177.2		21	26.1	1.31 (3H, s)
5	63.4		22	26.4	1.12 (3H, s)
6	48.5		23	21.5	1.21 (3H, s)
7	47.2	1.52 (1H, m)	24	29.9	2.07 (1H, m); 1.88 (1H, m)
8	38.9	2.10 (1H, m)	25	123.7	4.93 (1H, t, *J* = 6.5 Hz)
		2.39 (1H, dd, *J* = 14.5, 9.5 Hz)			
9	207.1		26	133.5	
10	173.0		27	18.2	1.51 (3H, s)
11	118.1		28	26.4	1.65 (3H, s)
12	109.9	8.12 (1H, s)	29	29.8	2.90 (2H, m)
13	148.1		30	120.9	5.62 (1H, t, *J* = 7.0 Hz)
14	155.7		31	134.7	
15	104.6	7.46 (1H, s)	32	18.3	1.65 (3H, s)
16	150.8		33	26.5	1.65 (3H, s)
17	27.8	2.04 (2H, m)			

Table S13. ^{1}H-NMR and ^{13}C-NMR of compound **14** (in pyridine-d_5)

No	^{13}C-NMR	^{1}H-NMR	No	^{13}C-NMR	^{1}H-NMR
1	55.7		20	26.2	1.48 (3H, s)
2	77.5		21	20.4	1.82 (3H, s)
3	123.3		22	18.2	0.85 (3H, s)
4	164.1		23	25.5	1.23 (3H, s)
5	61.9		24	29.5	1.67 (1H, m); 2.12 (1H, m)
6	49.6		25	123.5	5.05 (1H, br t, *J* = 6.5 Hz)
7	44.0	2.14 (1H, m)	26	133.4	
8	40.2	1.22 (1H, m); 3.06 (1H, m)	27	26.2	1.56 (3H, s)
9	211.4		28	18.2	1.52 (3H, s)
10	178.7		29	37.6	1.52 (1H, m)
					2.35 (1H, dd, *J* = 13.8, 4.9Hz)
11	116.7		30	39.4	2.91 (1H, m)
12	108.7	8.09 (1H, s)	31	147.4	
13	148.2		32	48.5	2.20 (1H, br d, *J* = 13.3 Hz)
					3.36 (1H, br d, *J* = 13.3 Hz)
14	156.1		33	109.7	4.93 (1H, br s); 4.96 (1H, br s)
15	104.2	7.45 (1H, s)	34	32.0	2.18 (1H, m); 2.44(1H, m)
16	152.1		35	124.4	5.42 (1H, br t, *J* = 6.5 Hz)
17	28.0	3.02 (1H, m); 3.04 (1H, m)	36	132.3	
18	120.4	5.00(1H, br t, *J* = 6.5 Hz)	37	26.2	1.67 (3H, s)
19	134.9		38	18.3	1.62 (3H, s)
